# Chinese patent medicine tongxinluo capsule as a supplement to treat chronic coronary syndromes: a GRADE-assessed systematic review and meta-analysis of randomized controlled trials

**DOI:** 10.3389/fcvm.2024.1499585

**Published:** 2025-01-07

**Authors:** Shi-Bing Liang, Yi-Fei Wang, Zhen-Chao Niu, Yu-Fei Li, Hui-Min Zheng, Jia-Ming Huan, Jie Yuan, Nicola Robinson, Jian-Ping Liu, Yun-Lun Li

**Affiliations:** ^1^ Clinical Study Center, Affiliated Hospital of Shandong University of Traditional Chinese Medicine, Jinan, China; ^2^ Centre for Evidence-Based Chinese Medicine, Shandong University of Traditional Chinese Medicine, Jinan, China; ^3^ Postdoctoral Research Station, Shandong University of Traditional Chinese Medicine, Jinan, China; ^4^ Cardiovascular Disease Center, Affiliated Hospital of Shandong University of Traditional Chinese Medicine, Jinan, China; ^5^ Centre for Evidence-Based Chinese Medicine, Beijing University of Chinese Medicine, Beijing, China; ^6^ The First School of Clinical Medicine, Shandong University of Traditional Chinese Medicine, Jinan, China; ^7^ Institute of Health and Social Care, London South Bank University, London, United Kingdom; ^8^ Shandong Engineering Laboratory of Traditional Chinese Medicine Precision Therapy for Cardiovascular Diseases, Shandong University of Traditional Chinese Medicine, Jinan, China

**Keywords:** chronic coronary syndromes, cardiovascular disease, Chinese herbal medicine, meta-analysis, systematic review, tongxinluo, clinical evidence

## Abstract

**Background:**

Chronic coronary syndromes (CCS) is a common clinical condition that increases the risk of cardiovascular events at any time. Tongxinluo capsules (TXL) are widely used in China for treating CCS.

**Objectives:**

To systematically evaluate the therapeutic effects and safety of adding TXL to Western medical treatment (WM) for CCS.

**Methods:**

We searched PubMed, Cochrane Library, CNKI, VIP, and Wanfang databases up to August 2024 for randomized controlled trials (RCTs) investigating the therapeutic effects and safety of combining TXL with WM compared to WM alone for CCS. Data analyses were conducted using RevMan 5.4 software.

**Results:**

Twenty studies involving 2091 participants were identified. Evidence supports the use of TXL plus WM for reducing angina frequency [SMD −2.50, 95% CI (−3.53, −1.48)], improving seattle angina questionnaire scores (*P* < 0.05), decreasing nitroglycerin dose [SMD −1.63, 95% CI (−2.26, −1.00)], and shortening angina duration [MD −1.50 min/once, 95% CI (−1.98, −1.02)]. Adding TXL to WM showed a non-significant trend toward reducing myocardial infarction [RR 0.34, 95% CI (0.05, 2.12); NNT = 41] and sudden cardiac death [RR 0.34, 95% CI (0.01, 8.28); NNT = 65]. No increase in adverse events was observed when TXL was added to WM [RR 1.02, 95% CI (0.70, 1.49); NNT = 149].

**Conclusions:**

Our review suggests that TXL may offer additional therapeutic benefits for CCS patients and appears to be safe when combined with WM. Further investigations are warranted to confirm the potential impact of adding TXL to WM for CCS.

**Systematic Review Registration:**

https://www.crd.york.ac.uk/prospero/display_record.php?ID=CRD42024499031, PROSPERO (CRD42024499031).

## 1 Background

Coronary artery disease (CAD) is characterized by the accumulation of atherosclerotic plaques in epicardial arteries (1). Globally, its prevalence ranges from 5% to 8% (2), with high morbidity and mortality, making it the major cause of death worldwide (3). In recent years, the incidence of CAD has risen notably in developing countries. The 2*019 ESC Guidelines for the diagnosis and management of chronic coronary syndromes* (Hereafter referred to as the “2019 ESC Guidelines”) categorize CAD as either acute coronary syndromes (ACS) or chronic coronary syndromes (CCS) (1). CCS refers to a chronic process that excludes acute coronary artery thrombosis-dominant conditions (i.e., ACS). It can be managed through lifestyle modifications, pharmacological therapies (such as anticoagulants, nitrates, vasodilators, antiplatelet agents, and lipid-lowering drugs), and invasive interventions aimed at stabilization or remission (1, 4). Data from the latest CLARIFY International Registry study indicate that cardiovascular events occurred in 8.0% of CCS patients over a five-year follow-up period, underscoring the need for intensive interventions (5).

Tongxinluo means “opening the network (luo) of the heart (xin) (6). Tongxinluo capsule (TXL) is a Chinese patent medicine based on traditional Chinese herbal theory, which has been clinically studied since 1995. Approved by China's State Food and Drug Administration in 1996 for treating angina pectoris and ischemic stroke, TXL is now included in China's national essential drug list (7). It is currently widely used for the treatment of arteriosclerotic cardiovascular disease in China and is composed of 12 Chinese herbs: *Panax ginseng* C.A. Mey (Ren Shen), *Paeonia lactiflora* Pall (Chi Shao), *Dryobalanops aromatica* C.F. Gaertn. (Bing Pian), *Cryptotympana pustulata* Fabricius (Chan Tui), *Hirudo nipponica* Whitman (Shui Zhi), *Ziziphus jujuba* Mill (Suan Zao Ren), *Dalbergia odorifera* T.C. Chen (Jiang Xiang), *Santalum album* L. (Tan Xiang), *Boswellia carterii* Birdw. (Ru Xiang), *Buthus martensii* Karsch (Quan Xie), *Scolopendra subspinipes mutilans* L. Koch (Wu Gong) and *Eupolyphaga sinensis* Walker (Tu Bie Chong) (8). TXL has demonstrated efficacy in reducing myocardial no-reflow and ischemic/reperfusion injury (9). Its mechanisms involve immune activation via antigen-antibody binding, inflammatory factors, cytokines, and signaling pathways, providing myocardial protection, vascular protection, stabilization of atherosclerotic plaques, vasodilation, hemodynamic improvement, and cardiac repair promotion (7, 10–12).

A randomized, double-blind, placebo-controlled clinical trial among patients with acute ST-segment elevation myocardial infarction (STEMI) within 24 h of symptom onset was conducted across 124 hospitals in China and published in JAMA in 2023 (6) The results showed that adding TXL to guideline-directed Western medicine treatments reduced 30-day major adverse cardiac and cerebrovascular events, cardiac death, myocardial reinfarction, and severe complications. A systematic review including 144 clinical trials involving 6,254 patients using TXL found that TXL was relatively safe, with no significant difference in adverse event occurrence compared to chemical drugs and other Chinese medicines (13). However, these studies focused primarily on patients with acute myocardial infarction (MI), nonvalvular atrial fibrillation, and ischemic stroke.

Given the incomplete evidence regarding the benefits and potential adverse effects of TXL for patients with CCS, there is a critical need for a systematic review and meta-analysis to provide new evidence on outcomes from TXL treatment in this patient population. This will help determine whether TXL can improve clinical outcomes for patients with CCS and contribute valuable insights into its therapeutic potential.

## 2 Methods

This systematic review and meta-analysis was conducted and reported in accordance with the Preferred Reporting Items for Systematic Review and Meta-Analysis (PRISMA) guidelines. The study protocol was registered on the International Prospective Register of Systematic Reviews PROSPERO (CRD 42024499031).

### 2.1 Study inclusion/exclusion criteria

#### 2.1.1 Types of studies

Randomized controlled trials (RCTs) were included. Non-RCTs, animal studies, trial protocols, conference articles or abstracts, conference proceedings, publications with only one author, dissertations and RCTs with misdescribed design elements (e.g., randomization methods, random concealment, and blinding) were excluded.

#### 2.1.2 Types of participants

Male or female adults of any age or ethnic origin with CCS, regardless of whether they were recruited through a hospital or a community health center, were included. All patients in the included RCTs were eligible for CCS, as described in the *2019 ESC Guidelines* (1). Participants who had undergone recent revascularization (within 3 months) were excluded.

#### 2.1.3 Types of interventions

The intervention was TXL combined with Western medical treatment (WM). There were no restrictions on TXL dosage or treatment duration. WM alone or a placebo for TXL combined with WM served as the control group. Both groups received identical WM. Studies with unclear or inconsistent treatment durations were excluded.

#### 2.1.4 Types of outcomes

Studies had to evaluate at least one of the following outcomes: cardiovascular endpoints (the use of revascularisation, including PCI and coronary artery bypass graft surgery, the occurrence of MI and sudden cardiac death), angina occurrence [frequency and duration, Seattle Angina Questionnaire (SAQ) scores, nitroglycerin dosage], quality of life, cost-effectiveness and adverse events. Cardiovascular endpoints were considered primary outcomes, while the others were secondary. All treatment and follow-up durations were eligible. For outcomes reported at multiple time points, we used data from the end of treatment and the longest follow-up period.

Six authors (SBL, YLL, YFW, ZCN, JMH and JY) collaborated to develop the inclusion/exclusion criteria for this review.

### 2.2 Search strategies for the identification of studies

A comprehensive search strategy was formulated to identify all relevant studies. The PubMed, Cochrane Library, Chinese National Knowledge Infrastructure (CNKI), VIP and Wanfang databases were searched from their inception to August 3rd, 2024.

The keywords used for searching were “Tongxinluo” OR “Tong Xin Luo” OR “Tong-Xin-Luo” OR “TXL” combined with “Chronic Coronary Syndromes” OR “CCS” OR “Coronary Artery Disease” OR “CAD” OR “Myocardial Ischemia” OR “MI” OR “Angina Pectoris” OR “AP” OR “Myocardial Infarction”, adjusted for use in different databases. Supplementary Material S1 displays the search strategies for all the databases. No language restrictions were applied.

Three authors (SBL, YFW and JMH) develop a search strategy for each database. To avoid missing potentially relevant RCTs, we checked the reference lists of included publications.

### 2.3 Study selection and data extraction

One author (SBL) scanned the titles and abstracts of every record retrieved for eligibility assessment. Full texts were reviewed when necessary for clarification or potential eligibility. Two authors (SBL, ZCN) independently selected studies by reviewing full texts, cross-checking selections, and resolving disagreements through discussion with a third author (YLL).

Three authors (SBL, HMZ and YFL) were responsible for the data extraction on the basis of a predesigned form for this review, including study titles, author information, characteristics of participants (age, sex, disease duration, number of participants randomized, number of patients analysed, etc.), details of interventions and comparators (dosage, treatment duration, etc.), outcomes (primary and secondary outcomes specified and collected, time points reported), and information relevant to the study design (e.g., randomization, allocation concealment, blinding, random concealment, and blinding). In this process, one author (SBL) independently completed the data extraction for all included studies, which was then verified separately by the other two authors (HMZ, YFL). Disagreements were resolved through discussion.

### 2.4 Assessment of risk of bias in included studies

The risk of bias for the primary outcome was assessed using the Cochrane risk of bias tool 2.0 (ROB 2.0) (14), covering five domains: (1) the randomization process, (2) deviations from the intended interventions, (3) missing outcome data, (4) measurement of the outcome, and (5) selection of the reported result. Each domain was assessed as (a) low risk, (b) some concerns or (c) high risk. Finally, overall risk bias was developed on the basis of the results of the above five domains in each trial. When four or more domains were rated “some concerns” or when at least one domain was rated “high risk”, the risk of overall bias was considered “high risk”; when all domains were rated “low risk”, the overall bias was considered “low risk”; otherwise, it was considered “some concerns”. Two authors (SBL, YFL) conducted the assessments, resolving inconsistencies through discussion.

### 2.5 Data synthesis

Data analysis was performed using Review Manager 5.4 (RevMan 5.4) software (15). The risk ratio (RR) with 95% confidence interval (CI) and the number needed to treat (NNT) (16) were used for dichotomous outcomes (cardiovascular endpoint events, adverse events). The NNT offers a measurement of the impact of a medicine or therapy by estimating the number of patients who need to be treated to have an impact on one person. For continuous outcomes {angina occurrence [frequency of angina occurrence, angina duration, Seattle angina questionnaire (SAQ) scores, nitroglycerin dosage], quality of life, and cost-effectiveness}, the mean difference (MD) with 95% CI was used when the outcome was measured by the same scale or with the same unit between different studies, and the standardized mean difference (SMD) with 95% CI was employed when different measurement scales or units were used. For outcome-related pretreatment continuous data, we performed a pooled analysis via the same methodology to confirm between-group comparability. Data that were unsuitable for meta-analysis, e.g., nonquantitative data, were presented descriptively.

We performed data synthesis (meta-analysis) when at least two studies were available. All meta-analyses were performed with a random effects model via the Mantel‒Haenszel (M-H) method, as potential sources of clinical heterogeneity exist between studies. We employed *I*^2^ statistics to quantify heterogeneity among studies: the value ranges between 0% and 100%, with higher values indicating greater statistical heterogeneity (17). When *I*^2^ > 50%, the data accuracy was checked first. If the data were accurate and appropriate, sensitivity analysis by excluding studies was conducted to explore the source of heterogeneity. Otherwise, the results would be carefully interpreted.

Sensitivity analysis was used to assess the robustness of the results between fixed effects and random effects analyses. Although we also planned to use sensitivity analyses to assess the impact of missing data on outcomes, the plan could not be implemented because most studies did not report whether data were missing.

Subgroup analysis was conducted according to the dose of TXL and the treatment duration to explore the effects of treatment duration and TXL dosage on the therapeutic effects.

### 2.6 Assessment of publication bias

A funnel plot was used to explore the possibility of publication bias if ten or more trials were included in a single meta-analysis (18).

### 2.7 Certainty assessment of the evidence developed in our review

The certainty of evidence was assessed using the GRADE (Grading of Recommendations Assessment, Development and Evaluation criteria) approach (19) via the GRADEpro Guideline Development Tool (GDT) online (https://gradepro.org/). Two authors (SBL, YFL) conducted the assessments, resolving inconsistencies through discussion.

## 3 Results

### 3.1 Results of the search

**Figure 1:**
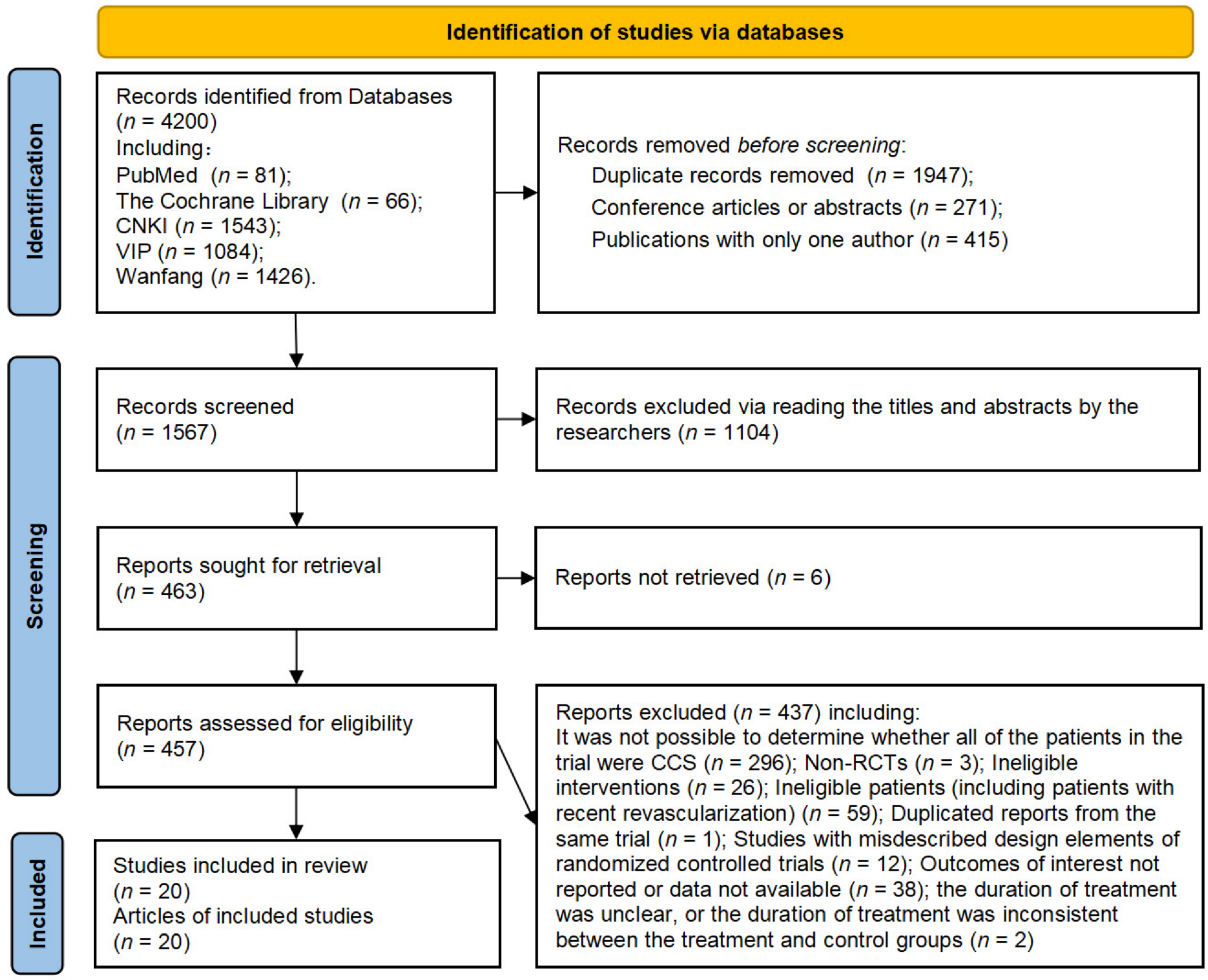
Flow diagram of the study retrieval and selection.

As illustrated in the flow diagram (Figure 1), our initial search of electronic bibliographic databases identified 4,200 records. After preliminary screening, 457 records were classified as potentially relevant and subjected to full-text assessment. No additional eligible studies were identified from the reference lists of included publications. Ultimately, 437 reports were excluded for various reasons, as detailed in Supplementary Material S2. This process resulted in the inclusion of 20 studies, reported in 20 published articles (20–39), that met our predefined inclusion criteria.

### 3.2 Description of studies

For a detailed description of the studies, the characteristics of the included studies are presented in Table 1.

**Table 1:** Characteristics of included studies.

Study	Location	Sample size (M/F)		Age (years old)		Course of disease (years)		Intervention	Comparator	Treatment duration	Outcomes
		I	C	I	C	I	C				
Cui et al. (20)	Hospital	32/33	38/32	66.37 ± 3.33 (62–78)	67.54 ± 3.40 (63–80)	7.00 ± 1.25 (5–11)	7.42 ± 1.00 (5.7–12)	TXL (4 capsules tid) + WM	WM: Anticoagulant drugs (Aspirin; Low-molecular heparin sodium); Nitrate drugs (Nitroglycerine, sublingually during angina attacks); β- receptor blocker; Vasodilator drugs (ACEI, used depending on the patient's condition); Antiplatelet aggregating agents (Clopidogrel; Tirofiban); Lipid-lowering drugs (Statins); etc.	12 weeks (3 months)	MI occurrence; Sudden cardiac death; AEs
Zhao et al. (21)	Hospital	29/27^a^	32/23^a^	60.23 ± 5.64	58.79 ± 5.21	5.02 ± 1.43	4.85 ± 1.27	TXL (4 capsules tid) + WM	WM: Anticoagulant drugs (Aspirin 100 mg qd); Nitrate drugs (Isosorbide Mononitrate 20 mg bid; Nitroglycerine, sublingually during angina attacks); β- receptor blocker (Metoprolol 25 mg bid); Lipid-lowering drugs (Statins 10 mg qd); Hypoglycaemic drugs (used depending on the patient's condition); etc.	12 weeks (3 months)	MI occurrence; Seattle angina questionnaire; AEs
Cheng and Li (22)	Hospital	23/17	25/15	52.18 ± 2.64 (35–70)	52.33 ± 2.81 (36–73)	8.59 ± 2.14 (1–19)	8.73 ± 2.21 (1.5–20)	TXL (4 capsules tid) + WM	WM: Anticoagulant drugs (Aspirin, Low-molecular heparin calcium); Nitrate drugs (Nitroglycerine, used depending on the patient's condition);β- receptor blocker (used depending on the patient's condition); Vasodilator drugs (ACEI, used depending on the patient's condition); Antiplatelet aggregating agents (Clopidogrel, starting dose of 300 mg qd, followed by dose of 75 mg qd); Lipid-lowering drugs (used depending on the patient's condition); Hypoglycaemic drugs (used depending on the patient's condition); etc.	12 weeks (3 months)	Angina frequency; angina duration; nitroglycerin dosage
Wang and Xing (23)	Hospital	37/23	39/21	58.47 ± 4.43 (48–70)	58.41 ± 4.38 (49–69)	4.34 ± 0.95 (2–7)	4.38 ± 0.97 (2–8)	TXL (4 capsules tid) + WM	WM:β- receptor blocker (Metoprolol 12.5 mg bid [Weeks 1 and 2], 25 mg bid [after Weeks 1 and 2]); Antiplatelet aggregating agents; etc.	12 weeks (3 months)	Angina frequency; angina duration; AEs
Wang et al. (24)	Hospital	30/12	28/14	60.38 ± 10.77 (49–72)	59.34 ± 10.86 (48–70)	6.05 ± 2.74 (3–9)	5.31 ± 2.23 (3–8)	TXL (4 capsules tid) + WM	WM:β- receptor blocker (Metoprolol 12.5 mg bid [Weeks 1 and 2], 25 mg bid [after Weeks 1 and 2]); Vasodilator drugs; Antiplatelet aggregating agents; Lipid-lowering drugs; etc.	12 weeks (3 months)	Angina frequency; angina duration; AEs
Wang and Kang (25)	Hospital	25/23	26/22	55.45 ± 4.85 (42–77)	55.48 ± 4.45 (42–78)	4.30 ± 1.67 (2–10)	4.30 ± 1.67 (2–10)	TXL (4 capsules tid) + WM	WM: Anticoagulant drugs; β- receptor blocker (Metoprolol 50 mg qd); Antiplatelet aggregating agents; Lipid-lowering drugs; Calcium Channel Blockers.	12 weeks (3 months)	AEs
Yang and Chen (26)	Hospital	31/25	30/26	60.8 ± 3.3 (46–80)	60.7 ± 3.5 (46–82)	5.44 ± 1.01 (3–7)	5.23 ± 1.03 (3–7)	TXL (2–4 capsules bid/tid) + WM	WM: Anticoagulant drugs (Aspirin; Low-molecular heparin, used depending on the patient's condition); Nitrate drugs (Isosorbide Mononitrate 20 mg bid; etc.);β- receptor blocker (Metoprolol; etc.); Antiplatelet aggregating agents (Clopidogrel 50 mg qd); Lipid-lowering drugs (Statins); etc.	4 weeks (1 month)	Angina frequency; angina duration; AEs
Zhu et al. (27)	Hospital	29/25	28/26	56.38 ± 6.17 (43–77)	55.93 ± 6.13 (42–77)	9.37 ± 1.28 (3–20)	9.33 ± 1.26 (3–21)	TXL (3 capsules tid) + WM	WM: Anticoagulant drugs (Aspirin 100 mg qd);β- receptor blocker (Metoprolol 12.5–25 mg bid); Lipid-lowering drugs (Statins 10 mg qd, used depending on the patient's condition); Calcium channel blockers (Amlodipine besylate tablets 5–10 mg qd, used depending on the patient's condition).	8 weeks (2 months)	Angina frequency; angina duration
Zhang et al. (28)	Hospital	35/25	38/22	64.81 ± 8.62 (52–78)	63.51 ± 8.22 (49–79)	NR	NR	TXL (3 capsules tid) + WM	WM: Anticoagulant drugs (Aspirin); Nitrate drugs (Nitroglycerine, sublingually during angina attacks); β- receptor blocker; Vasodilator drugs (ACEI; Trimetazidine dihydrochloride 20 mg tid).	4 weeks (1 month)	Angina frequency; angina duration; nitroglycerin dosage
Liu and Hua (29)	Hospital	47/43	45/45	66.4 ± 7.8	67.8 ± 6.7	8.3 ± 2.8	8.4 ± 2.9	TXL (4 capsules tid) + WM	WM: Anticoagulant drugs (Aspirin); Nitrate drugs; β- receptor blocker; etc.	4 weeks (1 month)	AEs
Xing and Wang (30)	Hospital; community health centre	28/14	27/15	62.0 ± 1.0 (41–72)	61.0 ± 1.5 (45–75)	6.21 ± 2.05 (1.6–9.7)	6.13 ± 2.10 (1.3–10.1)	TXL (4 capsules tid) + WM	WM: Nitrate drugs (Isosorbide Mononitrate 20 mg bid; Intravenous nitroglycerine 20–80 μg/min);β- receptor blocker; etc.	2 weeks (0.5 months)	AEs
Wei et al. (31)	Hospital	34/16	33/15	54 ± 5 (34–76)	55 ± 6 (36–75)	NR	NR	TXL (4 capsules tid) + WM	WM: Anticoagulant drugs (Aspirin); Nitrate drugs;β- receptor blocker; Vasodilator drugs (ACEI); Antiplatelet aggregating agents (Clopidogrel); Lipid-lowering drugs (Statins); Calcium channel blockers; etc.	12 weeks (3 months)	AEs
Li et al. (32)	Hospital	21/18	21/16	67 ± 15	65 ± 18	0.42–22	TXL (4 capsules tid) + WM	WM: Anticoagulant drugs (Aspirin); Nitrate drugs (Nitroglycerine, sublingually during angina attacks; etc.); Lipid-lowering drugs; etc.	8 weeks (2 months)	AEs	
Ye and Xia (33)	Hospital	31/19	32/18	66.7 ± 5.6 (60–76)	67.1 ± 6.2 (60–78)	NR	NR	TXL (3 capsules tid) + WM	WM: Anticoagulant drugs (Aspirin); Nitrate drugs (Isosorbide dinitrate 5 mg tid; Nitroglycerine, sublingually during angina attacks);β- receptor blocker; Lipid-lowering drugs; Calcium channel blockers; etc.	12 weeks (3 months)	Nitroglycerin dosage; AEs
Zhao and Xu (34)	Hospital	35/25	33/23	45–76	46–75	6.2	6.1	TXL (4 capsules tid) + WM	WM: Anticoagulant drugs (Aspirin); Nitrate drugs;β- receptor blocker; Lipid-lowering drugs (Statins); etc.	12 weeks (3 months)	AEs
Chen et al. (35)	Hospital	25/15	26/14	65.3 ± 11.2	64.1 ± 10.3	NR	NR	TXL (3 capsules tid) + WM	WM: Anticoagulant drugs (Aspirin); Nitrate drugs (Nitroglycerine, sublingually during angina attacks; etc.);β- receptor blocker; Vasodilator drugs (ACEI); Lipid-lowering drugs (Statins); Calcium channel blockers; Hypoglycaemic drugs (used depending on the patient's condition).	4 weeks (1 month)	AEs
Liu et al. (36)	Hospital	29/15	30/14	62.9 ± 8.4	61.2 ± 7.2	0.66 ± 0.37	0.72 ± 0.27	TXL (4 capsules tid) + WM	WM: Nitrate drugs (Isosorbide Dinitrate 10 mg tid);β- receptor blocker (Metoprolol 25–50 mg bid); Calcium channel blockers (Nifedipine tablets 20 mg qd)	4 weeks (1 month)	AEs
Liang et al. (37)	Hospital	22/18	25/15	67.56 ± 4.87 (38–74)	65.43 ± 4.21 (40–72)	9.18 ± 4.29 (1–20)	10.67 ± 5.12 (1–19)	TXL (2 capsules tid) + WM	WM	4 weeks (1 month)	Angina frequency; angina duration; nitroglycerin dosage; AEs
Wang and Ren (38)	Hospital	56/24	52/28	50 ± 14	51 ± 12	NR	NR	TXL (3 capsules tid) + WM	WM: Nitrate drugs (Isosorbide Dinitrate 10 mg tid [7am, 12 N, 5pm]; Nitroglycerine, sublingually during angina attacks)	8 weeks (2 months)	AEs
Jin and He (39)	Hospital	21/9	17/11	67–85	66–87	6–14	7–15	TXL (3 capsules tid) + WM	WM: Nitrate drugs (Isosorbide Dinitrate 10 mg tid)	4 weeks (1 month)	AEs

We checked through the website of the National Medical Products Administration (https://www.nmpa.gov.cn/) and found only one specification for TXL (0.26 g per capsule).

^a^ Per-Protocol Set. ACEI, angiotensin converting enzyme inhibitors; AEs, adverse events; ARBs, angiotensin II receptor antagonists; C, control group; f, female; I, intervention group; M, male; MI, myocardial infarction; NR, not reported; TXL, Tongxinluo capsule; WM, Western medical treatment.

#### 3.2.1 Design of the included studies

All included studies were parallel-design RCTs with a control group and were conducted in China; none employed a randomized, double-blind, placebo-controlled design. Nineteen studies were conducted in hospital settings, while one study (30) recruited participants through both hospital and community health centers. Five studies (20, 21, 24, 27, 33) reported receiving government funding, whereas the remaining studies did not provide information on funding sources.

#### 3.2.2 Participants in the included studies

A total of 2091 participants were randomly assigned to two groups (1,047 participants in the TXL group and 1,044 participants in the control group) in the 20 studies, and the number of participants ranged from 58 patients (39) to 180 patients (29) in the different studies. Two studies (20, 21) reported the presence of patient loss to follow-up. Participant ages ranged from 34 to 87 years, and disease duration varied from 5 months to 22 years.

#### 3.2.3 Interventions in the included studies

All studies compared TXL plus WM with the same WM alone. TXL was administered as 2–4 capsules bid/tid (0.26 g per capsule) immediately upon enrollment in all studies. WM used in the control group consisted of anticoagulant drugs (aspirin, low-molecular-weight heparin), nitrate drugs (nitroglycerine, isosorbide mononitrate, etc.), β-receptor blockers (metoprolol, etc.), vasodilator drugs (angiotensin converting enzyme inhibitors, angiotensin II receptor antagonists, etc.), antiplatelet aggregating agents (clopidogrel, tirofiban, etc.), lipid-lowering drugs (statins, etc.) and calcium channel blockers (amlodipine besylate tablets, nifedipine tablets, etc.), etc. Treatment durations ranged from 2 to 12 weeks (3 months). Detailed information on the WM used in each study is summarized in Table 1, with a comprehensive overview provided in Supplementary Material S3.

#### 3.2.4 Outcomes reported in the included studies

Regarding the primary outcome of cardiovascular endpoint events, 2 studies (20, 21) reported MI occurrence, 1 study (20) mentioned the occurrence of sudden cardiac death, and no studies reported the use of revascularization. Regarding the outcome of angina, 7 studies (22–24, 26–28, 37) reported the frequency of angina occurrence, 7 studies (22–24, 26–28, 37) measured the angina duration, 1 study (21) assessed angina by using the SAQ, and 4 studies (22, 28, 33, 37) reported data available for nitroglycerin dosage. Seventeen studies (20, 21, 23–26, 29–39) reported adverse events. No studies reported quality of life or cost-effectiveness.

#### 3.2.5 Risk of bias in the included studies

##### 3.2.5.1 Risk of bias for each domain in the included studies

**Figure 2:**
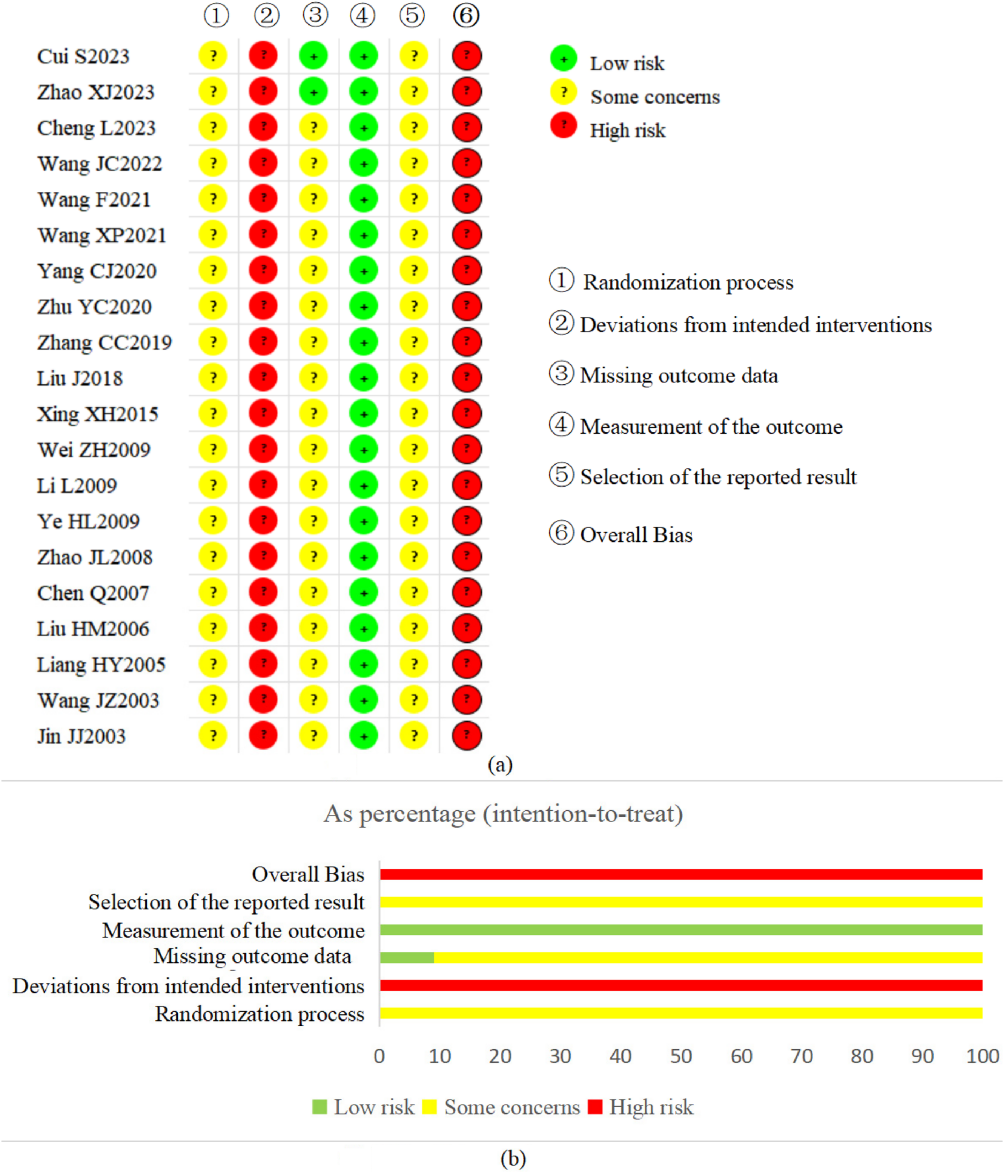
Risk of bias assessment in included studies. **(a)** Risk of bias summary. **(b)** Risk of bias graph.

The risk of bias assessment for each domain in the included studies is summarized in Figure 2.

Domain 1: Randomization process.

Nine studies (20, 22, 31, 33–36, 38, 39) mentioned only “random” without further clarification, and the other 11 studies (21, 23–30, 32, 37) reported detailed methods of random sequence generation, including the random number table method, the touchball method, the lottery method and Excel software. The baseline data were comparable between the groups in each study. No study reported information on allocation concealment. If allocation concealment was not implemented, there is reason to suspect that the enrolling investigator or the participant knew the forthcoming allocation (14, 40). Considering the above, all studies were judged as “some concerns” in this domain.

Domain 2: Deviations from intended interventions

There was no available information for any of these studies on either participant or clinician blinding. However, in light of the interventions from the studies, all the studies failed to carry out blinding for participants and clinicians who delivered the interventions. Therefore, participants were aware of their assigned intervention during the study, and clinicians delivering the interventions were aware of participants’ assigned intervention during the trial. These studies also did not report whether deviations arose because of the study context, and the information to assess if the analysis was appropriate was insufficient. Therefore, all studies were judged as “high risk of bias” in this domain.

Domain 3: Missing outcome data

When complete outcome data were available or data from few participants (<10%) were missing, 2 studies (20, 21) were judged as having a “low risk of bias” in this domain. The other 18 studies were considered “some concerns” because there was no information about the integrity of the outcome data. Specifically, these studies did not adequately report how they managed missing data or ensured the completeness and accuracy of their results, which raises uncertainties about the reliability of the findings.

Domain 4: Measurement of the outcome

Although no study carried out blinding for clinicians delivering the interventions or reported whether the outcome assessor was independent of the clinician, all studies were assessed as “low risk of bias” in this domain considering that all the evaluated outcomes were objective.

Domain 5: Selection of the reported result

No study reported information on their protocol, so we could not judge whether they selectively reported their outcomes. Therefore, all studies were judged as “some concerns” in this domain. We did attempt to search clinical trial registries and contact trial authors for protocol information, but unfortunately, these efforts did not yield the necessary details.

##### 3.2.5.2 Overall bias in the included studies

Considering the assessments across all domains, all studies were determined to have an overall “high risk of bias”. The results are presented in Figure 2.

### 3.3 Effects of interventions

#### 3.3.1 Occurrence of cardiovascular endpoint events

##### 3.3.1.1 Occurrence of MI

**Figure 3:**
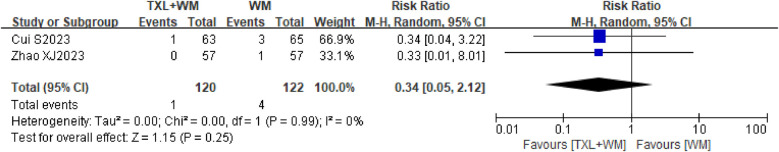
Forest plot of myocardial infarction (MI) occurrence. TXL, tongxinluo capsule; WM, western medical treatment.

A pooled analysis of two studies (20, 21) involving 242 participants revealed a nonsignificant effect in favour of adding TXL (4 capsules tid, 0.26 g per capsule) to WM (1/120 vs. 4/122, M-H, RR = 0.34, 95% CI 0.05–2.12, *P* = 0.25, random effects, Figure 3; NNT = 41) at the end of 3 months (12 weeks) of treatment. The sensitivity analysis revealed that there was no difference between the random effects and fixed effects (RR = 0.34, 95% CI 0.05–2.12; *P* = 0.25, fixed effects; Supplementary Figure S1) in the meta-analyses of effect sizes. Planned subgroup analyses were not carried out due to the limited number of studies included.

##### 3.3.1.2 Sudden cardiac death

In one study (20), one case of sudden cardiac death occurred in the control group, while no cases were reported in the TXL (4 capsules tid, 0.26 g per capsule) group. The calculated NNT is 65. There was no statistically significant difference between the groups (0/63 vs. 1/65, M-H, RR = 0.34, 95% CI 0.01–8.28, *P* = 0.51, 12 weeks of treatment). Due to the inclusion of only one study, prespecified sensitivity and subgroup analyses could not be conducted.

#### 3.3.2 Angina occurrence

##### 3.3.2.1 Angina frequency

**Figure 4:**
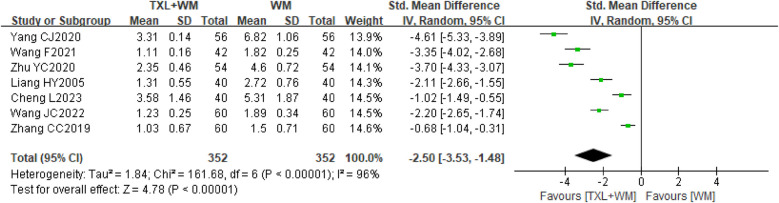
Forest plot of angina frequency. TXL, tongxinluo capsule; WM, western medical treatment.

Seven RCTs (22–24, 26–28, 37) involving 704 participants had data available for the angina frequency investigated at the end of treatment (1st or 3rd month) and compared TXL (2–4 capsules bid/tid, 0.26 g per capsule) plus WM with WM alone. The pooled analysis of baseline angina frequency data revealed no significant difference between the TXL and control groups before treatment (SMD = −0.01, 95% CI −0.16 to 0.13, *P* = 0.85; random effects; Supplementary Figure S2). After treatment, the meta-analysis indicated that TXL plus WM was significantly better at reducing the angina frequency (SMD = −2.50, 95% CI −3.53 to −1.48, *P* < 0.00001, *I*^2^ = 96%, random effects; Figure 4).

The sensitivity analysis demonstrated that there was only a slight difference between random effects and fixed effects (SMD = −1.98, 95% CI −2.17 to −1.78, *P* < 0.00001, *I*^2^ = 96%, fixed effects; Supplementary Figure S3) meta-analyses on effect sizes, and both were in favour of the addition of TXL. Although we tried to explore the sources of heterogeneity by excluding studies that might introduce heterogeneity (with the goal of decreasing *I*^2^ to less than 50% after excluding relevant studies), we did not obtain meaningful findings after several attempts. However, after qualitative comparative analyses of the characteristics of the included studies, we considered that the heterogeneity may be caused by clinical factors (e.g., differences in treatment periods, TXL dosages, controls and outcome measures).

A predefined subgroup analysis according to the currently available information was conducted for angina frequency. Two studies (27, 37) were not included in the subgroup analysis because they used different measurement units of angina frequency (times/day; times/month) than most studies did (times/week). Subgroup analysis according to the TXL dosage and treatment duration revealed an important effect difference between the subgroups [MD = −1.99 times/week, 95% CI −4.97 to 0.99, 4 weeks, 2–4 capsules bid/tid, 2 trials; MD = −0.74 times/week, 95% CI −0.92 to −0.56, 12 weeks (3 months), 4 capsules tid, 3 trials; Supplementary Figure S4], but the subgroup difference was not statistically significant (*P* = 0.41, Supplementary Figure S4).

##### 3.3.2.2 Angina duration

**Figure 5:**
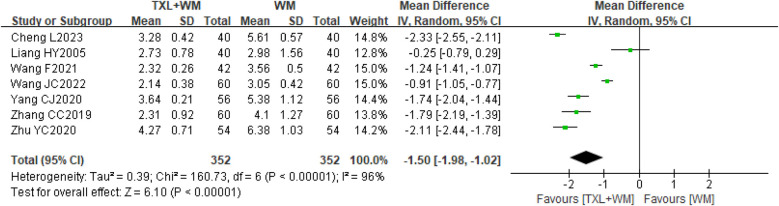
Forest plot of angina duration. TXL, tongxinluo capsule; WM, western medical treatment.

Seven studies (22–24, 26–28, 37) involving 704 participants reported the outcome of angina duration evaluated at the end of treatment (1st or 3rd). The pooled analysis of baseline angina duration data revealed no significant difference between the groups (MD = 0.10 min/time, 95% CI −0.09 to 0.28, *P* = 0.32; random effects; Supplementary Figure S5). However, the pooled analysis of these seven studies after treatment revealed that the addition of TXL (2–4 capsules bid/tid, 0.26 g per capsule) to WM shortened the duration of angina compared with WM alone (MD = −1.50 min/once, 95% CI −1.98 to −1.02 min/once, *P* < 0.00001, *I*^2^ = 96%, random effects; Figure 5).

Sensitivity analysis indicated a minor discrepancy between the random-effects and fixed-effects models regarding effect sizes (MD = −1.39 min/time, 95% CI −1.48 to −1.30, *P* < 0.00001, *I*^2^ = 96%, fixed-effect; Supplementary Figure S6). Despite multiple efforts to pinpoint the source of heterogeneity by omitting various studies, the most successful adjustment only reduced *I*^2^ to 75% (Supplementary Figure S7), offering limited insight into the underlying causes. However, qualitative comparative analyses suggested that clinical factors—such as variations in treatment duration, TXL dosages, and control conditions—might be contributing to the observed heterogeneity.

A pre-specified subgroup analysis was performed based on available data concerning angina duration. The analysis based on the TXL dose and treatment duration revealed small differences in effects between subgroups but statistically significant differences between the 2 group comparisons within each subgroup (MD = −1.29 min/once, 95% CI −2.11 to −0.47, 4 weeks, 2–4 capsules bid/tid, 3 trials; MD = −2.11 min/once, 95% CI −2.44 to −1.78, 8 weeks, 3 capsules tid, 1 trial; MD = −1.49 min/once, 95% CI −2.25 to −0.73, 12 weeks (3 months), 4 capsules tid, 3 trials; Supplementary Figure S8), and the subgroup differences were statistically significant (*P* = 0.09, Supplementary Figure S8).

##### 3.3.2.3 SAQ scores

The SAQ is a 19-item self-administered, disease-specific patient-reported outcome with five domains: physical limitations, anginal stability, anginal frequency, treatment satisfaction, and disease perception/quality of life (41). All domain scores and summary scores range from 0 to 100, with higher scores indicating improvement (42).

Meta-analysis was not performed because of the availability of data from only one study (21). The study reported no statistically significant difference in SAQ dimension scores between the two groups before treatment (*P* > 0.05). However, after treatment, the SAQ scores significantly increased compared to the pre-treatment period (*P* < 0.05). Moreover, when TXL (4 capsules tid, 0.26 g per capsule, 12-week treatment) was added to the WM, it resulted in statistically significant improvements across different dimensions of the SAQ compared to the WM alone (*P* < 0.05).

##### 3.3.2.4 Nitroglycerin dosage

**Figure 6:**
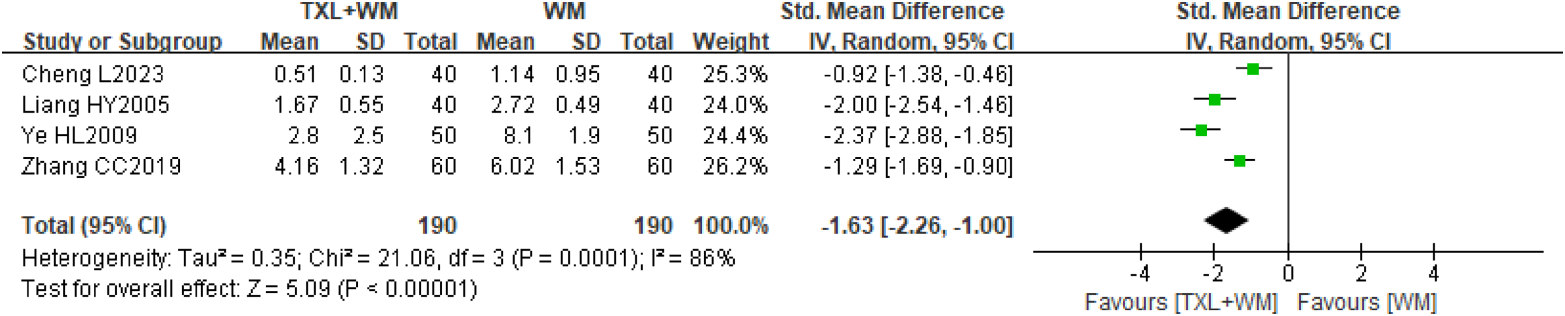
Forest plot of nitroglycerin dosage. TXL, tongxinluo capsule; WM, western medical treatment.

Four studies (22, 28, 33, 37) consisting of 380 participants assessed nitroglycerin dosage at the end of treatment (either 1st or 3rd month). Baseline data revealed no significant difference in nitroglycerin dosage between the groups (SMD = 0.20, 95% CI −0.01 to 0.40, *P* = 0.06, random effects; Supplementary Figure S9). Nonetheless, all studies showed that adding TXL (2–4 capsules tid, 0.26 g per capsule) to WM treatment led to a reduction in nitroglycerin usage compared to WM alone. The pooled analysis confirmed this trend with a statistically significant effect favoring the addition of TXL (SMD = −1.63, 95% CI −2.26 to −1.00, *P* < 0.00001, *I*^2^ = 86%, random effects; Figure 6).

A sensitivity analysis comparing random effects and fixed effects models revealed minor discrepancies (SMD = −1.55, 95% CI −1.78 to −1.32, *P* < 0.00001, *I*^2^ = 86%, fixed effects; Supplementary Figure S10). Attempts to reduce heterogeneity succeeded in lowering *I*^2^ to 0% and 31% (Supplementary Figures S11, 12), but these efforts did not elucidate the source of heterogeneity. Clinical factors such as variations in treatment duration, TXL dosages, controls, and outcome measures were identified as potential sources through qualitative comparative analyses of the included studies. Given the limited number of studies, planned subgroup analyses for this outcome were not performed.

#### 3.3.3 Occurrence of adverse events

**Figure 7:**
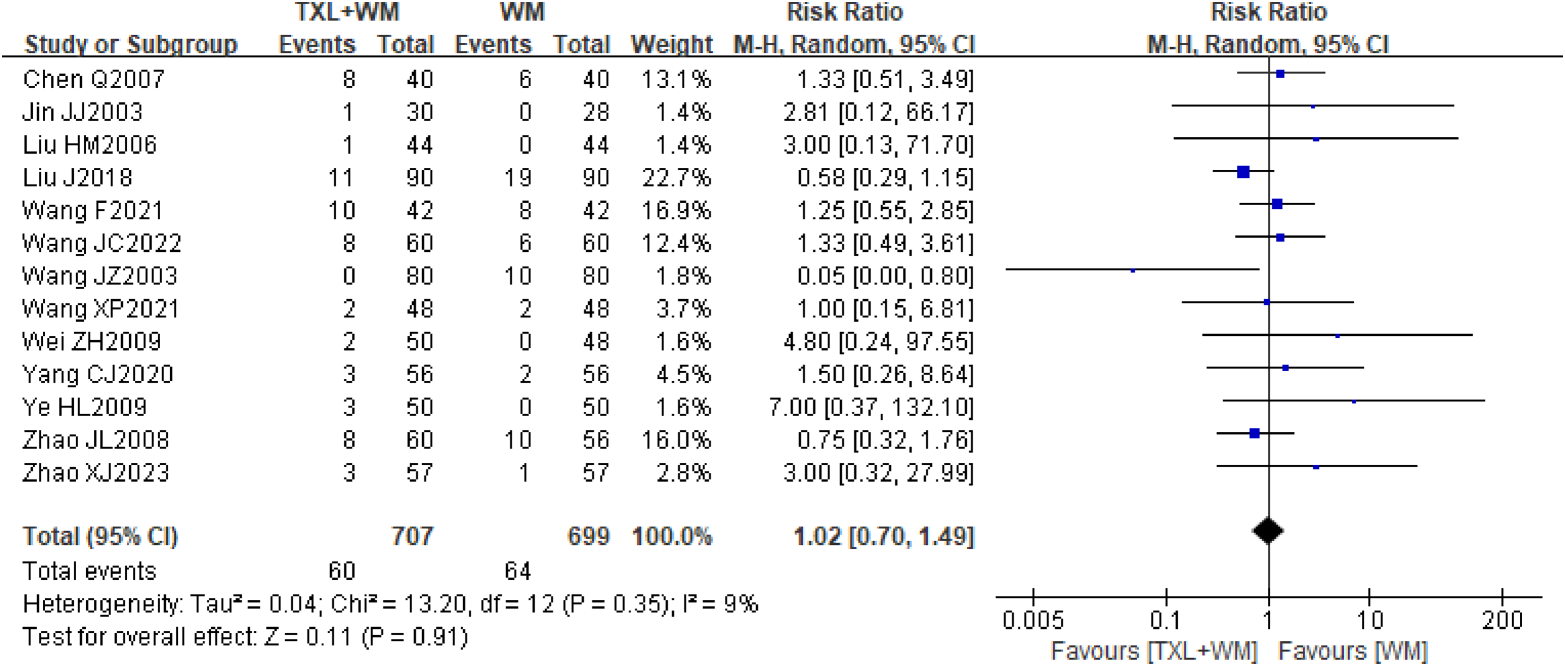
Forest plot of adverse events. TXL, tongxinluo capsule; WM, western medical treatment.

Seventeen studies involving 1,783 participants reported on the occurrence of adverse events. Among these, four studies (20, 30, 32, 37) reported no adverse events in either the intervention or control groups during treatment periods ranging from 14 days to 3 months, Consequently, the analysis focused on data pooled from the remaining 13 studies (21, 23–26, 29, 31, 33–36, 38, 39), which covered treatment durations of 1–3 months. There was no evidence of a difference between the add-on TXL (2–4 capsules bid/tid, 0.26 g per capsule) group and the control group (60/707 vs. 64/699, M-H, RR = 1.02, 95% CI 0.70–1.49, *P* = 0.91, random effects, Figure 7; NNT = 149). The adverse events that occurred in both groups included mainly abdominal discomfort and gastrointestinal symptoms such as nausea and vomiting.

Sensitivity analysis comparing the results of random effects and fixed effects models showed virtually no discrepancy (RR = 0.93, 95% CI 0.67–1.28, *P* = 0.66; Supplementary Figure S13), indicating robustness of the findings.

### 3.4 Publication bias

**Figure 8:**
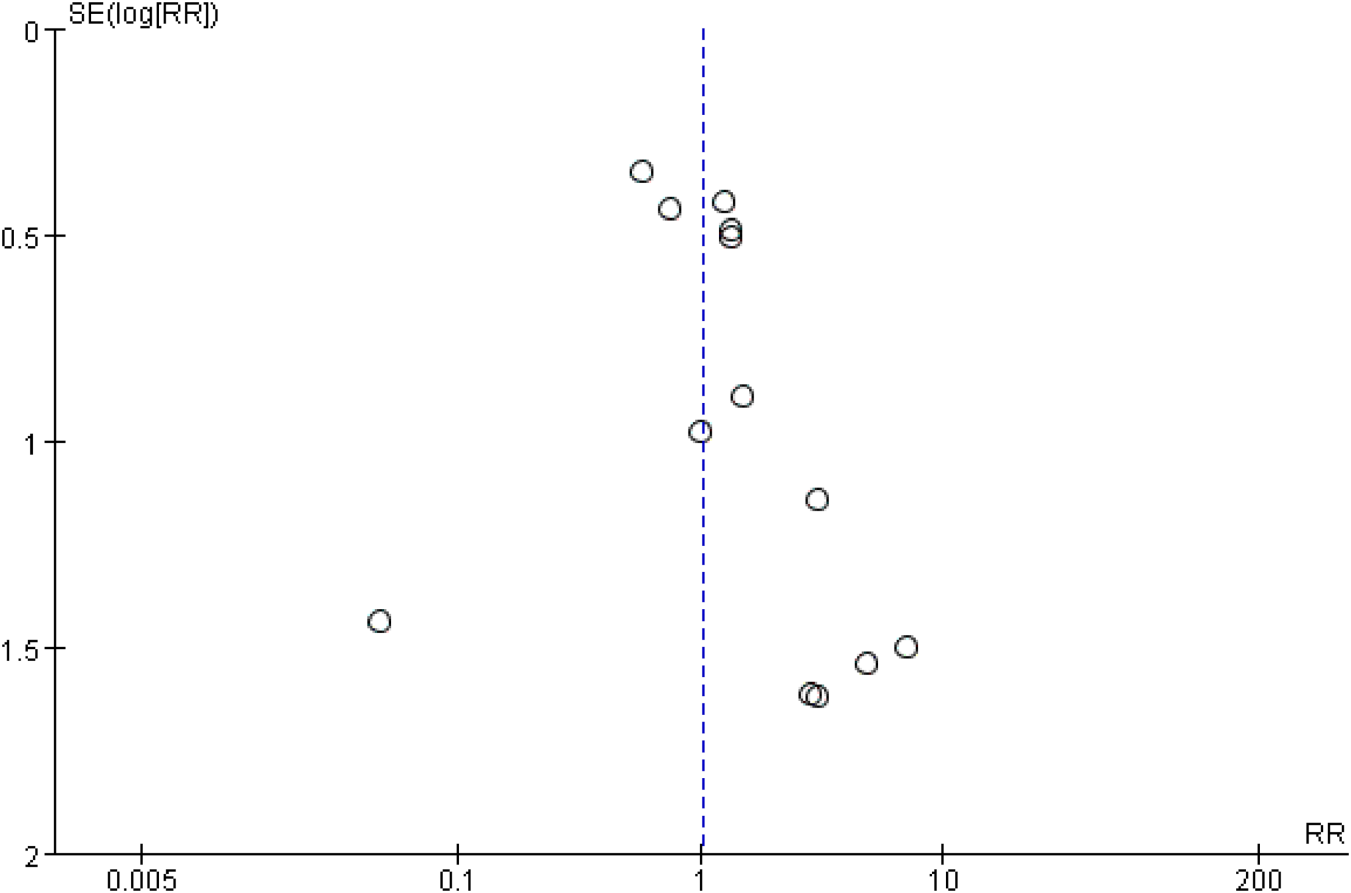
Funnel plot for the outcome of adverse events.

A funnel plot was utilized to assess potential publication bias for adverse event outcomes, given that more than ten studies were included in the meta-analysis. The asymmetrical funnel plot suggested a high risk of publication bias (Figure 8).

### 3.5 GRADE certainty of evidence

The certainty of evidence for outcomes was evaluated as low. They were downgraded mainly due to the high risk of performance bias (studies included failed to carry out blinding for participants and clinicians delivering the interventions), imprecision (small number (<300) of total events, RR with a relatively wide confidence interval), and large heterogeneity between the included studies and/or suspected publication bias. Table 2 presents details of the evaluation of the certainty of the evidence for the outcomes.

**Table 2:** Summary of evidence certainty for outcomes.

Patient or population: Chronic coronary syndromes Setting: Hospital or community health center Intervention: Tongxinluo capsule plus Western medical treatment Comparison: Western medical treatment						
Outcomes	Anticipated absolute effects^a^ (95% CI)		Relative effect (95% CI)	No. of participants (studies)	Certainty of evidence (GRADE)	Comments
	Risk with comparison	Risk with intervention				
Occurrence of MI	33 per 1,000	11 per 1,000 (2–70)	RR 0.34 (0.05–2.12)	242 (2 RCTs)	⊕⊕◯◯ Low	Certainty of evidence downgraded two levels due to a and b.
Sudden cardiac death	NA	NA	NA	NA	NA	Evidence certainty of this outcome was not evaluated as only 1 study was included.
Frequency of angina occurrence	–	SMD 2.34 lower (3.24 lower to 1.44 lower)	–	785 (8 RCTs)	⊕⊕◯◯ Low	Certainty of evidence downgraded two levels due to a and c.
Angina duration	–	MD 1.50 lower (1.98 lower to 1.02 lower)	–	704 (7 RCTs)	⊕⊕◯◯ Low	Certainty of evidence downgraded two levels due to a and c.
SAQ scores	NA	NA	NA	NA	NA	Evidence certainty of this outcome was not evaluated as only 1 study was included.
Nitroglycerin dosage	–	SMD 1.57 lower (2.06 lower to 1.07 lower)	–	461 (5 RCTs)	⊕⊕◯◯ Low	Certainty of evidence downgraded two levels due to a and c.
Adverse events	92 per 1,000	93 per 1,000 (64–136)	RR 1.02 (0.70–1.49)	1,406 (13 RCTs)	⊕⊕◯◯ Low	Certainty of evidence downgraded two levels due to a and d.

Reasons for the downgrade of evidence certainty: a. high risk of performance bias (studies included failed to carry out blinding for participants and clinicians delivering the interventions); b. imprecision (small number (< 300) of total events, RR with a relatively wide confidence interval); c. a large heterogeneity existed between the included studies; d. suspected of publication bias.

CI, confidence interval; NA, not applicable; RR, risk ratio; RCT, randomized controlled trial; SAQ, Seattle angina questionnaire; SMD, standardized mean difference.

GRADE Working Group grades of evidence.

High certainty: we are very confident that the true effect lies close to that of the estimate of the effect.

Moderate certainty: we are moderately confident in the effect estimate: the true effect is likely to be close to the estimate of the effect, but there is a possibility that it is substantially different.

Low certainty: our confidence in the effect estimate is limited: the true effect may be substantially different from the estimate of the effect.

Very low certainty: we have very little confidence in the effect estimate: the true effect is likely to be substantially different from the estimate of effect.

^a^ The risk in the intervention group (and its 95% confidence interval) is based on the assumed risk in the comparison group and the relative effect of the intervention (and its 95% CI).

## 4 Discussion

### 4.1 Summary of the main results

To our knowledge, this systematic review and meta-analysis is the first to compare patients with CCS treated with TXL and WM with those treated with only WM. Twenty RCTs involving 2,091 CCS participants that evaluated the therapeutic effects and safety of TXL as a supplement were included. The analysis results demonstrated that TXL in combination with WM was more effective for improving clinical outcomes in people with CCS. Two trials reported the occurrence of cardiovascular endpoint events (MI and sudden cardiac death), our primary outcome of interest, and suggested that the addition of TXL may reduce the risk of cardiovascular endpoint events, possibly resulting in a positive effect, although the estimates failed to achieve statistical significance. The results of the NNT analysis suggest that treating an average of 45 people with TXL would result in one person experiencing MI, and treating an average of 60 people with TXL would result in one person experiencing sudden cardiac death. There was also some evidence from this analysis that suggested that the addition of TXL to WM reduced the risk of angina occurrence, judging from the reduction in angina frequency, the shortening of angina duration, the increase in SAQ scores and the reduction in nitroglycerin dosage. Seventeen studies reported the occurrence of adverse events and demonstrated that the addition of TXL did not increase the risk of adverse events.

Notably, while these studies provide data on efficacy and safety, they do not specifically address potential pharmacodynamic interactions between TXL and WM. This means that the specific mechanisms by which these two treatments might interact remain unclear. However, the absence of increased adverse events suggests that TXL likely does not significantly interfere with the pharmacodynamics of WM; otherwise, it might have led to a higher incidence of side effects or adverse reactions.

However, our confidence in the effect estimate is limited, and the study findings should be interpreted with caution because all studies were at high risk of bias and all meta-analysis evidence had low-GRADE certainty.

### 4.2 Implications for future practice and trials

Currently, in clinical practice in China, TXL is often used for the treatment of CCS together with WM. This review has important implications for future practice, as the strength of the evidence available has been systematically identified, appropriately analysed and appraised. These findings suggest that adding TXL to WM may further enhance therapeutic benefits for patients with CCS without increasing the risk of adverse events. Although there was no significant difference between add-on TXL and WM alone in reducing the risk of cardiovascular endpoint events, TXL as a supplement appeared to have greater potential for improving angina occurrence. In summary, TXL can potentially improve certain outcomes for patients with CCS treated with WM. On the basis of the characteristics of the included studies and the results of the subgroup analyses and safety profile, we suggest that clinicians use TXL (4 capsules tid) with a treatment duration of no less than 12 weeks (3 months), in addition to WM (antithrombotic therapy, lipid-lowering drugs, etc.) recommended by the 2019 ESC Guidelines (1)^,^ to treat CCS patients. However, it remains to be determined if extending treatment beyond 12 weeks offers additional benefits. During treatment, if adverse events such as abdominal discomfort and gastrointestinal symptoms occur in patients, an appropriate reduction in the TXL dosage to 2–3 capsules per time and/or twice daily can be attempted, but the therapeutic effects may be reduced. However, this needs to be further confirmed in future studies.

Our research also has important implications for future trials. First, due to the high risk of bias in the existing studies, further high-quality research is needed, particularly multicenter, placebo-controlled, and blinded RCTs. Improvements in methodological quality, including a valid method of randomization, allocation concealment, blinding, should be achieved. Second, the aims of the pharmacological management of CCS patients described in the 2019 ESC Guidelines (1) are to reduce angina symptoms and exercise-induced ischaemia and to prevent cardiovascular events. In future studies on TXL for CCS, more attention should therefore be given to the evaluation of these endpoints. Third, as an additional and complementary treatment to WM, TXL inevitably increases healthcare costs, necessitating a comparative evaluation of its cost-effectiveness. However, no included studies reported this information. Therefore, we recommend including cost-effectiveness outcomes in future studies to better inform medical decision-making. Furthermore, since quality of life improvements are critical for all patients, and none of the included studies reported on this outcome, we recommend that future studies also assess quality of life. Additionally, to ensure a more rigorous and comprehensive study design, as well as better dissemination and application of results, we recommend that future studies adhere to the Standard Protocol Items: Recommendations for Interventional Trials (SPIRIT) guidelines (43). Study protocols should be developed and registered in advance on WHO-recognized platforms, such as ClinicalTrials.gov. Researchers should report their studies in accordance with the Consolidated Standards of Reporting Trials (CONSORT) statement (44).

### 4.3 Strengths and limitations

Our findings may provide new insights and evidence for the use of TXL in the treatment of CCS, leading to more options for CCS patients in clinical practice. We strictly followed the process and specifications for producing systematic reviews as presented in the Cochrane Handbook (17). Efforts were made to minimize bias throughout the review process. The research protocol was developed by researchers in evidence-based medicine (SBL), along with clinicians (YLL, YFW, ZCN, JMH and JY) and cardiovascular disease researchers (YLL and YFW), ensuring that our research was conducted correctly and maximizing the value of the evidence for clinical practice. The review was conducted in strict accordance with the protocol registered on PROSPERO (CRD 42024499031). A comprehensive search without language restrictions was performed to identify all relevant studies on the topic. Study selection, risk of bias assessment, and data extraction were carried out independently or cross-checked by two review authors. Additionally, we assessed the certainty of evidence from all meta-analyses using the GRADE method. Thus, our research provides additional dimensions of reference that will add value to future clinical practice and research.

Potential limitations of this study should be acknowledged. Firstly, all the trials were conducted in China, which may limit the generalizability of the findings to patients from other countries or regions with different healthcare settings and patient populations. Secondly, the sample sizes of most included studies were relatively small, indicating that studies with larger sample sizes are necessary to provide stronger support for our findings. Moreover, despite standardizing the results to SMD, heterogeneity persisted in some meta-analyses of continuous outcomes. We attempted to explore possible sources of heterogeneity using various methods, including sensitivity analyses and subgroup analyses, but specific sources remained unidentified due to the limited number of included studies and the scarcity of detailed information available from these studies. However, qualitative comparative analyses of the study characteristics led us to conclude that clinical factors (such as variations in treatment duration, TXL dosages, control conditions, and outcome measures) were likely contributors to the observed heterogeneity.

## 5 Conclusions

The available evidence from RCTs suggests possible benefits of adding TXL to WM in patients with CCS, including reducing the frequency and duration of angina, improving SAQ scores, and decreasing nitroglycerin dosage. The use of TXL appears to be safe. However, more robust evidence on the role of TXL in the management of CCS is needed before a firm recommendation for clinical practice can be made. Therefore, further investigations are warranted.

## Data Availability

The original contributions presented in the study are included in the article/Supplementary Material, further inquiries can be directed to the corresponding author.
