# Supplementary material for: Chinese patent medicine tongxinluo capsule as a supplement to treat chronic coronary syndromes: a GRADE-assessed systematic review and meta-analysis of randomized controlled trials
Source: Front Cardiovasc Med. 2025 Jan 7;11:1499585. doi: 10.3389/fcvm.2024.1499585 (PMC11753206; doi:10.3389/fcvm.2024.1499585)
Supplement: Supplementary file 1 [file Datasheet1.pdf]

Supplementary Material 1 Search Strategies and results

(1) PubMed: 2024/08/03 (81 studies)

(((((Tongxinluo[Title/Abstract]) OR (Tong Xin Luo[Title/Abstract])) OR (Tong-Xin-Luo[Title/Abstract])) OR (TXL[Title/Abstract])) AND ((((((((((Chronic Coronary Syndromes[Title/Abstract]) OR (CCS[Title/Abstract])) OR (Coronary Artery Disease[Title/Abstract])) OR (CAD[Title/Abstract])) OR (Coronary Heart Disease[Title/Abstract])) OR (Myocardial Ischemia[Title/Abstract])) OR (MI[Title/Abstract])) OR (Angina Pectoris[Title/Abstract])) OR (AP[Title/Abstract])) OR (Myocardial Infarction[Title/Abstract])))

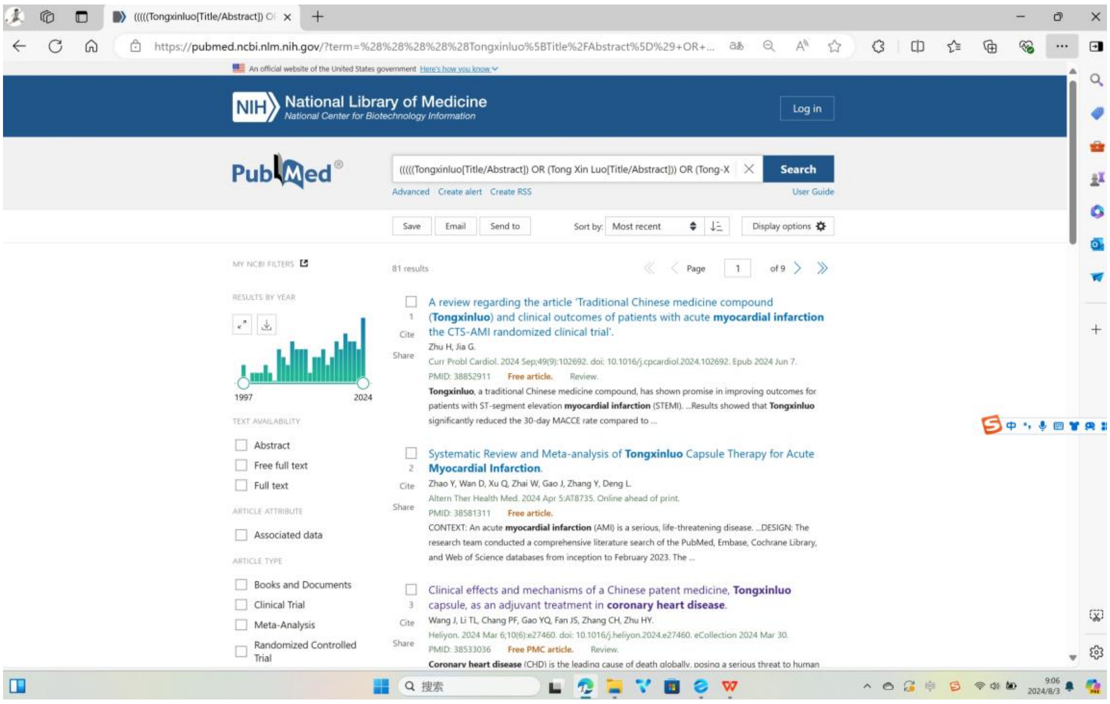

(2) the Cochrane Library: 2024/08/03 (66 studies)

(((((Tongxinluo) OR (Tong Xin Luo)) OR (Tong-Xin-Luo)) OR (TXL)) AND ((((((((((Chronic Coronary Syndromes) OR (CCS)) OR (Coronary Artery Disease)) OR (CAD)) OR (Coronary Heart Disease)) OR (Myocardial Ischemia)) OR (MI)) OR (Angina Pectoris)) OR (AP)) OR (Myocardial Infarction))) in Title Abstract Keyword

(3) CNKI: 2024/08/03 (1543 studies)

(((TI = '通心络') AND (TI = '冠心病' OR TI = '冠脉综合征' OR TI = '冠状动脉综合征' OR TI = '心绞痛' OR TI = '心肌缺血' OR TI = '心肌梗死' OR TI = '胸痹')) AND (FT = '随机'))

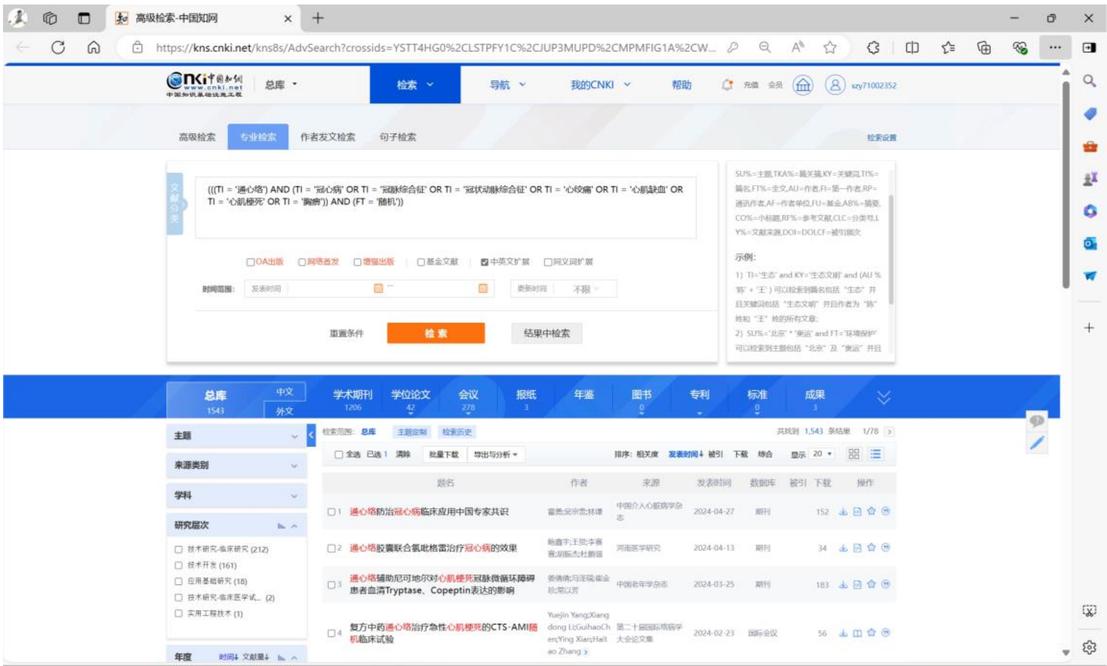

(4) VIP: 2024/08/03 (1084 studies)

(((T = 通心络) AND (T = 冠心病 OR T = 冠脉综合征 OR T = 冠状动脉综合征

OR T = 心绞痛 OR T = 心肌缺血 OR T = 心肌梗死 OR T = 胸痹)) AND (U = 随机))

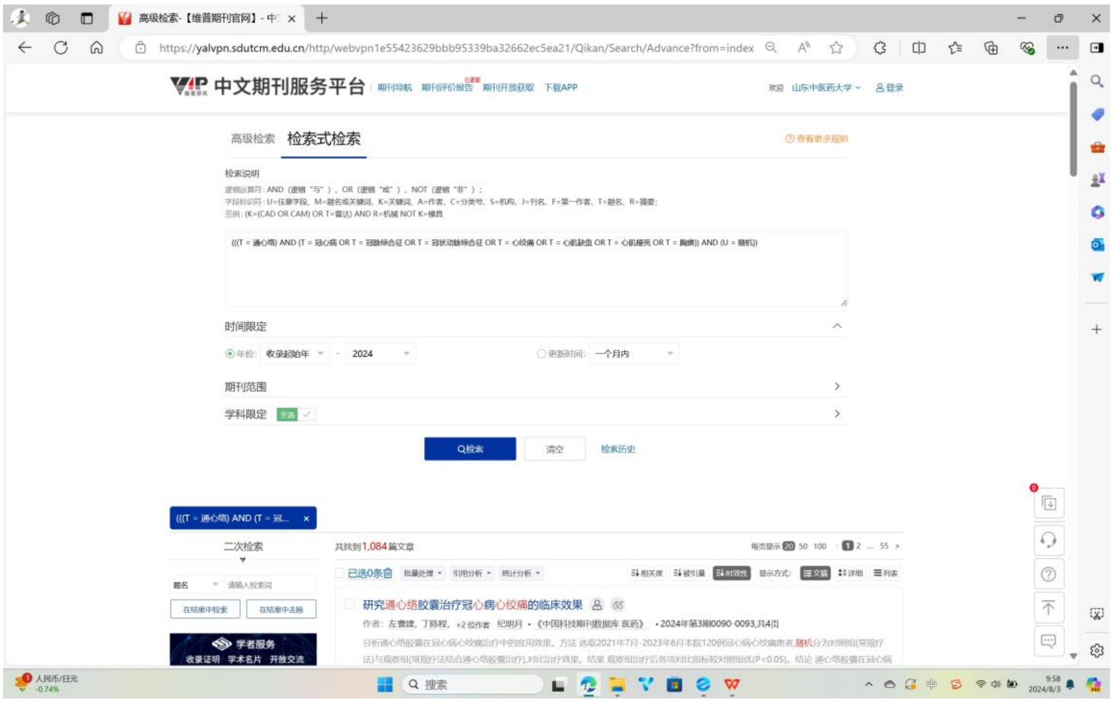

(5) Wanfang: 2024/08/03 **(1426 studies)**

(((题名:(通心络)) and (题名:(冠心病) or 题名:(冠脉综合征) or 题名:(冠状动脉综合征) or 题名:(心绞痛) or 题名:(心肌缺血) or 题名:(心肌梗死) or 题名:(胸痹))) and (全部:(随机)))

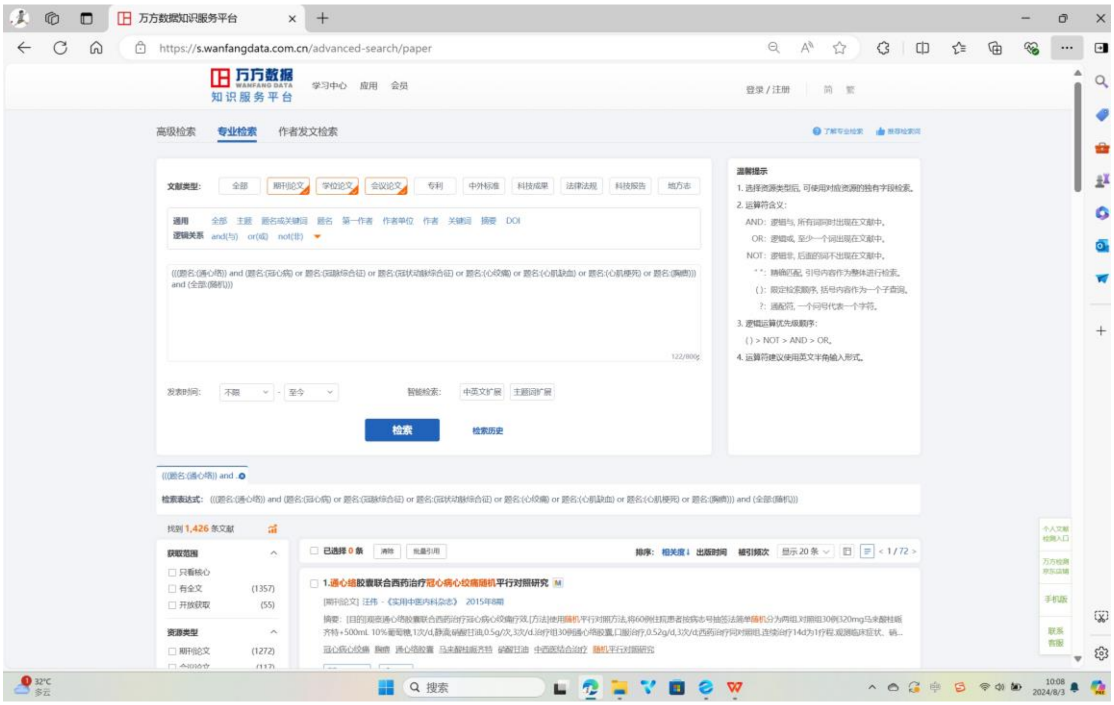
万方数据知识服务平台
https://s.wanfangdata.com.cn/advanced-search/paper
高级检索 专业检索 作者发文检索
文献类型: 全部 期刊论文 学位论文 会议论文 专利 中外标准 科技成果 法律法规 科技报告 地方志
(((题名:(通心络)) and (题名:(冠心病) or 题名:(冠脉综合征) or 题名:(冠状动脉综合征) or 题名:(心绞痛) or 题名:(心肌缺血) or 题名:(心肌梗死) or 题名:(胸痹))) and (全部:(随机)))
检索
检索历史
找到 1,426 条文献
1.通心络胶囊联合西药治疗冠心病心绞痛随机平行对照研究
