# Supplementary material for: Chinese patent medicine tongxinluo capsule as a supplement to treat chronic coronary syndromes: a GRADE-assessed systematic review and meta-analysis of randomized controlled trials
Source: Front Cardiovasc Med. 2025 Jan 7;11:1499585. doi: 10.3389/fcvm.2024.1499585 (PMC11753206; doi:10.3389/fcvm.2024.1499585)
Supplement: Supplementary file 2 [file Datasheet2.pdf]

Supplementary Material 2 Reports excluded by reading full texts

No.	Excluded studies with different reasons
1	It was not possible to determine whether all of the patients in the trial were CCS (n = 296)

[1] Zuo CJ, Ding CC, Wang XW, et al. Study on the clinical effect of Tongxinluo capsule in the treatment of angina pectoris in coronary heart disease (研究通心络胶囊治疗冠心病心绞痛的临床效果). Medicine of China Science and Technology Journal Database, 2024(3):90-93.

[2] Wang DF, Liu CH, Li XF, et al. Clinical effect of Tongxinluo capsule combined with nicorandil tablets in the treatment of angina pectoris in coronary heart disease (通心络胶囊联合尼可地尔片治疗冠心病心绞痛的临床效果). Chinese Journal of Clinical Rational Drug Use, 2024,17(7):1-4.

[3] Xian LJ, Zhang XG, Han BW, et al. Effect of Tongxinluo treatment on serum Hs-CRP and Hcy levels in patients with coronary heart disease angina pectoris (通心络治疗对冠心病心绞痛患者血清 Hs-CRP 和 Hcy 水平的影响). Experimental and Laboratory Medicine, 2023,41(05):621-623.

[4] Zhao WB, Guo XT. Clinical efficacy of clopidogrel combined with Tongxinluo capsule in the treatment of angina pectoris in coronary heart disease (氯吡格雷联合通心络胶囊治疗冠心病心绞痛的临床疗效). Special Health, 2023(13): 161-162.

[5] Chen ZJ, Huang XX, Xiao SW. Effects of Tongxinluo capsule combined with atorvastatin treatment on blood lipid levels and cardiac function in elderly patients with coronary heart disease (通心络胶囊联合阿托伐他汀治疗对老年冠心病患者血脂水平及心功能的影响). Chinese Journal of Gerontology, 2023,43(13): 3086-3088.

[6] Zhang JQ, Zhang TM. Effect of Tongxinluo Capsule Combined with Atorvastatin Calcium on Patients with Coronary Heart Disease and Carotid Atherosclerotic Plaque (通心络胶囊联合阿托伐他汀钙对冠心病并颈动脉粥样硬化斑块患者的疗效探讨). China Foreign Medical Treatment, 2023, 42(13): 98-101.

[7] Hu XW, Zeng QH, Li K, et al. The effect of Tongxinluo capsule in the treatment of angina pectoris in coronary heart disease and its effect on patients' cardiac function (通心络胶囊治疗冠心病心绞痛的效果及其对患者心功能的影响). Journal of clinical rational drug use, 2022, 15(19): 1-3.

[8] Liang HH, Lv Z. Effect of Tongxinluo capsule combined with felodipine sustained release tablets in the treatment of angina pectoris and its influences on NT-proBNP level and platelet activity (通心络胶囊联合非洛地平缓释片治疗心绞痛的效果及对 NT-proBNP 水平、血小板活性的影响). Clinical Research and Practice, 2022, 7(34): 159-163.

[9] Liu YJ, Liu YH, Zhu J, et al. Effect of Tongxinluo combined with trimetazidine on ventricular remodeling and left ventricular function in patients with coronary heart disease and heart failure (通心络联合曲美他嗪对冠心病心力衰竭患者心室重塑及左心功能的影响). Modern Journal of Integrated Traditional Chinese and Western Medicine, 2022, 31(08): 1120-1123.

[10] Sun XY, Zhang YL, Wang LP, et al. Effect of Tongxinluo Capsules Combined with Metoprolol on QT Dispersion, Vascular Endothelial Function and Heart Rate Turbulence in Patients with Coronary Heart Disease Complicated with Ventricular Premature Beats (通心络胶囊联合美托洛尔对冠心病合并室性早搏患者 QT 离散度、血管内皮功能及心率震荡的影响). Journal of New Chinese Medicine, 2022, 54(7): 55-59.

[11] Gao Q, Xu L, Chen WH. Effect of Tongxinluo Capsule Combined with Atorvastatin Calcium Tablets on Coronary Heart Disease Complicated with Hyperlipidemia (通心络胶囊联合阿托伐他汀钙片治疗冠心病合并高脂血症的效果研究). China Foreign Medical Treatment, 2022, 41(35): 176-180.

[12] Zhu XL, Zhang L. Effect of Tongxinluo Capsule Combined with Metoprolol Tartrate in the Treatment of Coronary Heart Disease in the Elderly (通心络胶囊联合酒石酸美托洛尔治疗老年人冠心病的疗效). Shenzhen Journal of Integrated Traditional Chinese and Western Medicine, 2022, 32(03): 38-41.

[13] Chen LF, Chen YJ. Observation of the efficacy of Tongxinluo combined with nicorandil in the treatment of unstable angina (通心络联合尼可地尔治疗不稳定型心绞痛疗效观察). Medicine and health, 2022(9): 67-70.

[14] Zhang BX, Li JL, Chu YH. Effects of Tongxinluo combined with enhanced external counterpulsation on vascular endothelial function, myocardial perfusion level and cardiac function in patients with coronary heart disease and angina pectoris (通心络联合增强型体外反搏对冠心病心绞痛患者血管内皮功能、心肌灌注水平和心功能的影响). Clinical Research and Practice, 2022, 7(30): 44-47.
[15] Liu Y, Qi WJ. Clinical effect of Metoprolol combined with Tongxinluo in the treatment of coronary heart disease and angina pectoris (美托洛尔联合通心络治疗冠心病心绞痛的临床疗效). Doctor, 2021, 6(8): 5-7.
[16] Xin PC, Niu SQ, Xu L, et al. Effect of Tongxinluo capsule treatment on the symptoms and quality of life of patients with coronary heart disease and angina (冠心病心绞痛患者采用通心络胶囊治疗对病症与生活质量的干预效果). Heilongjiang Journal of Traditional Chinese Medicine, 2021, 50(01): 152-153.
[17] Cao Y, Mei DH. Observation on the Clinical Effect of Tongxinluo Capsule Combined with Rosuvastatin Calcium in the Treatment of Angina Pectoris of Coronary Heart Disease (通心络胶囊联合瑞舒伐他汀钙治疗冠心病心绞痛的临床效果观察). Clinical Medicine & Engineering, 2021, 28(10): 1361-1362.
[18] Wang QY, Zhang JZ, Liu RJ. The efficacy of Tongxinluo capsule combined with rosuvastatin in the treatment of patients with coronary heart disease and heart failure and its effect on vascular endothelial function (通心络胶囊联合瑞舒伐他汀治疗冠心病合并心力衰竭患者的疗效及其对血管内皮功能的影响). Journal of Internal Medicine Department, 2021, 16(2): 156-159.
[19] Yu Y, Qi WM. Effects of Tongxinluo capsules combined with metoprolol on cardiac function and blood lipid levels in patients with coronary heart disease and angina pectoris (通心络胶囊联合美托洛尔对冠心病心绞痛患者心功能与血脂水平的影响). Modern Medicine and Health Research Electronic Journa, 2021, 5(5): 30-33.
[20] Wang MQ, Lin YH, Huang QF, et al. Analysis of the clinical efficacy of clopidogrel combined with Tongxinluo capsule in the treatment of coronary heart disease angina (氯吡格雷联合通心络胶囊治疗对冠心病心绞痛的临床疗效分析). Knowledge of Cardiovascular Disease Prevention and Control: Academic Edition, 2021, 11(6): 40-42.
[21] Fang PJ, Li J. Study on the clinical efficacy of betalux combined with Tongxinluo in the treatment of angina pectoris of coronary heart disease (倍他乐克联合通心络治疗冠心病心绞痛的临床疗效研究). Journal of Dietary Health, 2021(17):74.
[22] Yang F, Liu XS, Xia Q, et al. Clinical Study of Tongxinluo Capsule Combined with Bisoprolol in the Treatment of Angina Pectoris of Coronary Heart Disease (通心络胶囊联合比索洛尔治疗冠心病心绞痛的临床研究). China Foreign Medical Treatment, 2021, 40(1): 100-102.
[23] Zhang T, Zhang C. The clinical efficacy of Tongxinluo capsule in patients with coronary heart disease and diabetes mellitus (通心络胶囊对冠心病合并糖尿病患者临床疗效). Continuing Medical Education, 2020, 34(05): 167-168.
[24] Zhao GF, Song XX. Discussion on the clinical effect of betalux combined with Tongxinluo therapy in patients with coronary heart disease and angina (冠心病心绞痛患者倍他乐克联合通心络治疗的临床效果探讨). Health Essentials, 2020(34): 59.
[25] Fan HX, Shi WQ. Efficacy of Tongxinluo Capsule combined with rosuvastatin in the treatment of elderly coronary heart disease complicated with heart failure (通心络胶囊联合瑞舒伐他汀治疗老年冠心病合并心力衰竭的疗效). Healthy Friends, 2020(15): 115.
[26] Qiao WW, Chen YJ, Si DN. Effect of Tongxinluo Capsule on angina pectoris of coronary heart disease (通心络胶囊治疗冠心病心绞痛的效果). clinical medicine, 2020, 40(7): 107-109.
[27] Gong Y, Zhang W. Observation of the clinical effect of Tongxinluo capsule combined with bisoprolol fumarate in the treatment of angina pectoris of coronary heart disease (通心络胶囊联合富马酸比索洛尔治疗冠心病心绞痛临床效果观察). Chronic Pathematology Journal, 2020, 0(1): 94-95.
[28] Chen L, Liu HX, Chen Y. Obzevatian, Bengt Clini, Effic Teben, Correspondence, Roccapsul, Combine, Nes, Bissau, Prol, Fumarat, Inte Trite, Mente, Ben Angela, Pektoris, Bachelor, Ronal, Richt, Disias (探讨心肌缺血患

者应用通心络胶囊联合曲美他嗪治疗的临床效果). World Latest Medicine Information, 2020, 20(9): 184, 186.

[29] Xu JB, Shao LF. Clinical study of Tongxinluo Capsules combined with trimotaziaine in treatment of myocardial ischemia in elderly (通心络胶囊联合曲美他嗪治疗老年心肌缺血的临床研究). Drug Evaluation Research, 2020, 43(10): 2065-2068.

[30] Gu YC, Sun FZ. Discussion on the efficacy of Tongxinluo capsule combined with rosuvastatin in the treatment of elderly patients with coronary heart disease (通心络胶囊联合瑞舒伐他汀治疗老年冠心病患者的疗效探讨). Medical Diet and Health, 2020, 18(05): 78-79.

[31] Zhu Y, Cao JC, Yang D. Observation of the efficacy of Tongxinluo Capsule combined with atorvastatin in the treatment of angina pectoris in coronary heart disease and its effect on the expression of plasma matrix metalloproteinase 9, endothelin 1 and hypersensitive C-reactive protein in patients (通心络胶囊联合阿托伐他汀治疗冠心病心绞痛疗效观察及对患者血浆基质金属蛋白酶 9、内皮素 1 和超敏 C 反应蛋白表达的影响). Chinese Journal of Primary Medicine and Pharmacy, 2020, 27(22): 2781-2785.

[32] Zhang ZX, Feng Q, Qi NN. Effect of trimetazidine combined with Tongxinluo capsule on ventricular remodeling in patients with coronary heart disease and heart failure (曲美他嗪联合通心络胶囊对冠心病心力衰竭患者心室重构的影响). Cardiovascular Disease Journal Of integrated traditional Chinese and Western Medicine, 2020, 8(17): 50-51.

[33] Wang C, Dong DF. Effect of Tongxinluo Capsule combined with Trimetazidine on cardiac ejection function in patients with coronary heart disease angina (通心络胶囊联合曲美他嗪对冠心病心绞痛患者心脏射血功能的影响). Modern Practical Medicine, 2020, 32(02): 258-260.

[34] Yan GD, Zhang ZD. To investigate the clinical effect of atorvastatin combined with Tongxinluo capsule in the treatment of unstable angina pectoris pectoris (探讨冠心病不稳定型心绞痛采用阿托伐他汀联合通心络胶囊治疗的临床效果). Journal of North Pharmacy, 2020, 17(02): 91-92.

[35] Chen FX, Liang YH, Huang AZ. Effect of the treatment of elderly patients with arrhythmias with Tongxinluo capsules and metoprolol tartrate tablets (用通心络胶囊和酒石酸美托洛尔片对老年冠心病心律失常患者进行治疗的效果). Contemporary Medicine Forum, 2019, 17(19): 131-132.

[36] Zhang SW, Wang BZ, Wang CH. A clinical comparative study of Tongxinluo capsule in the treatment of coronary heart disease (通心络胶囊治疗冠心病的临床对比研究). Heilongjiang Journal of Traditional Chinese Medicine, 2019, 48(05): 89-90.

[37] Mao YN, Liu G. Clinical observation of tongxinluo capsules in treatment of angina pectoris in the patients (通心络胶囊治疗心绞痛患者的临床观察). World Journal of Integrated Traditional and Western Medicine, 2019, 14(7): 970-973.

[38] Wang XW, Wang JP. Clinical Analysis of the Combination of Betaloc and Tongxinluo in the Treatment Of Angina Pectoris (倍他乐克、通心络联合治疗冠心病心绞痛的临床效果分析). World Latest Medicine Information, 2019, 19(24): 15-16.

[39] Dai WY, Gao WF, Chen P. Clinical Study of Metoprolol Combined with Tongxinluo Capsules for Coronary Heart Disease (美托洛尔联合通心络胶囊治疗冠心病临床研究). Journal of New Chinese Medicine, 2019, 51(5): 116-118.

[40] Wu HD, Hu YR. Effect of Tongxinluo and Betaloc on serum inflammatory factor in patients with coronary heart disease and angina pectoris (通心络联合倍他乐克对冠心病心绞痛患者血炎症因子的影响分析). Journal of Frontiers of Medicine, 2019, 9(12): 160-161.

[41] Liu LJ, Zhu J, Bao J, et al. Clinical study of Tongxinluo Capsules combined with Amlodipine Besylate and Atorvastatin Calcium Tablets in the treatment of angina pectoris of coronary heart disease (通心络胶囊联合氨氯地平阿托伐他汀钙片治疗冠心病心绞痛的临床研究). China Medical Herald, 2019, 16(32): 135-139.

[42] Wang Y, Liu XL, Shi YT, et al. Analysis of the effect of Tongxinluo capsule in the treatment of elderly patients with coronary heart disease (通心络胶囊治疗老年冠心病患者的效果分析). Cardiovascular Disease

Journal Of integrated traditional Chinese and Western Medicine, 2019, 7(20): 54.

[43] Yuan JW, Gao ZQ, Li S, et al. Analysis of Clinical Effects of Tongxinluo Combined With Aspirin in the Treatment of Unstable Angina Pectoris (通心络联合阿司匹林治疗不稳定型心绞痛疗效分析). Continuing Medical Education, 2019, 33(01): 148-150.

[44] Yang JD, Zhu SG, Ma Y. Effect of Tongxinluo combined with western medicine on elderly patients with unstable angina pectoris (通心络联合西药治疗老年不稳定性心绞痛的效果). Clinical Research and Practice, 2019,4(2):122-123.

[45] Ge Q, Zhou JY, Wu WF. Efficacy of Tongxinluo Capsule combined with rosuvastatin in the treatment of elderly coronary heart disease complicated with heart failure (通心络胶囊联合瑞舒伐他汀治疗老年冠心病合并心力衰竭的疗效). Anhui Medical Journal, 2018, 39(10): 1264-1267.

[46] Huang DJ, Ni SF. Effect of metoprolol succinate combined with Tongxinluo capsule on serum hs-CRP and BNP levels in patients with coronary heart disease (琥珀酸美托洛尔联合通心络胶囊对冠心病患者血清 hs-CRP 及 BNP 水平的影响). Strait Pharmaceutical Journal, 2018,30(12):78-80.

[47] Li YM, Wang XG. Evaluation of the clinical effect of metoprolol tartrate tablets in patients with angina pectoris pectoris (冠心病心绞痛患者应用酒石酸美托洛尔片通心络联合治疗的临床效果评价). Electronic Journal of Clinical Pharmaceutical Literature, 2018, 5(92): 77.

[48] Zhao QQ, Li DF, Zhang XC. Clinical study of Tongxinluo capsule combined with trimetazidine in the treatment of myocardial ischemia in the elderly patients (通心络胶囊联合曲美他嗪治疗老年心肌缺血的临床研究). Practical Geriatrics, 2018,32(4):348-352.

[49] Huang FR, Li YH, Chu QM. A study on the efficacy of conventional treatment of western medicine combined with Tongxinluo capsule in the treatment of angina pectoris of coronary heart disease (西药常规治疗联用通心络胶囊治疗冠心病心绞痛疗效的研究). Journal of Electrocardiography, 2018, 7(04): 53-54.

[50] Jiang L, Liu Y. Observation of the clinical effect of Tongxinluo capsule in the treatment of angina pectoris of coronary heart disease (通心络胶囊治疗冠心病心绞痛的临床效果观察). Health and Wellness Guide, 2018(22): 61.

[51] Shi XJ, li XN. Observation of the efficacy of Tongxinluo capsule in patients with angina pectoris with small vessel lesions (通心络胶囊在小血管病变心绞痛患者中的应用疗效观察). New Medicine, 2018, 28(A01): 652.

[52] Yang JF, Qiao J. Observation of the effect of metoprolol succinate combined with Tongxinluo capsule in the treatment of angina pectoris of coronary heart disease (琥珀酸美托洛尔联合通心络胶囊治疗冠心病心绞痛的效果观察). Journal of Frontiers of Medicine, 2018, 8(27): 94-95.

[53] Sun CX, Sun SW, Li SL, et al. Observation of the efficacy of Western medicine in combination with Tongxinluo capsule in the treatment of angina pectoris of coronary heart disease (西药常规联合通心络胶囊治疗冠心病心绞痛疗效观察). Journal of Dietary Health, 2018, 5(13): 76.

[54] Qiao RF, Xu CY. Evaluation of the effect of Tongxinluo combined with clopidogrel in the treatment of angina pectoris in coronary heart disease (通心络联合氯吡格雷治疗冠心病心绞痛的效果评价). China Health Care Nutrition, 2018, 28(31): 11.

[55] Dong X, Shao MX. Study on the Effect of Tongxinluo on Endothelial Cell Function in Patients With Coronary Heart Disease (通心络改善冠心病患者内皮细胞功能的相关研究). China Health Standard Management, 2018, 9(03): 97-99.

[56] Guo PB, Dong L, Shen XQ, et al. Effect of Tongxinluo Capsule on vascular endothelial function in patients with coronary heart disease angina (通心络胶囊对冠心病心绞痛患者血管内皮功能的影响). Chinese Journal of Rural Medicine and Pharmacy, 2018, 25(15): 18-19.

[57] Cai X, Dan XC. Clinical Evaluation on Tongxinluo Capsules Combined with Benidipine Hydrochloride Tablets for Treating Coronary Spastic Angina in 164 Cases (通心络胶囊联合盐酸贝尼地平片治疗冠状动脉痉挛性心绞痛 164 例临床评价). China Pharmaceuticals, 2018,27(8):52-55.

[58] Liu XJ, Zeng CY. The efficacy of Tongxinluo capsule in the treatment of unstable angina pectoris and the effect of blood rheology (通心络胶囊治疗不稳定型心绞痛疗效及血液流变学的影响). Strait Pharmaceutical Journal, 2018, 30(04): 117-118.

[59] Liu YP, Li SJ, Ye L. Observation of the short-term effect of diltiazem combined with Tongxinluo capsule in the treatment of patients with unstable angina (地尔硫卓联合通心络胶囊治疗不稳定型心绞痛患者的短期效果观察). Henan Medical Research, 2018, 27(20): 3703-3705.

[60] Nan MH, Jiao XM, Li S, et al. Efficacy and Safety Analysis of Tongxinluo Capsule for Qi Deficiency and Blood Stasis Type Unstable Angina Pectoris (通心络胶囊治疗气虚络瘀型不稳定型心绞痛有效性和安全性分析). Journal of Liaoning University of Traditional Chinese Medicine, 2018, 20(06): 156-159.

[61] Ren JJ, Guo P, Li XL. Analysis of the effect of aspirin combined with Tongxinluo in the treatment of unstable angina (阿司匹林联合通心络治疗不稳定型心绞痛的效果分析). Electronic Journal of Clinical Pharmaceutical Literature, 2018, 5(31): 72.

[62] Ding T, Zhou L. To explore the clinical efficacy of Tongxinluo Capsule in the treatment of unstable angina pectoris of coronary heart disease (探讨通心络胶囊治疗冠心病不稳定型心绞痛的临床疗效). World Latest Medicine Information, 2018, 18(A1): 212-213.

[63] Chen H, Wu LY, Zheng DG, et al. Clinical Observation of Tongxinluo Capsules Combined with Clopidogrel Hydrogensulfate Tablets for Unstable Angina Pectoris (通心络胶囊联合硫酸氯吡格雷片治疗不稳定型心绞痛临床观察). Journal of New Chinese Medicine, 2018, 50(02): 9-12.

[64] Hui H, Zhang M, Peng ZG, et al. Effect of Tongxinluo Capsule on cardiac enzymes and electrocardiogram in patients with unstable angina (通心络胶囊对不稳定性心绞痛患者心肌酶及心电图的影响). Chinese Journal of Gerontology, 2018, 38(09): 2080-2082.

[65] Chang GD, Chen YW, Xu XS, et al. Curative effect and mechanism of Tongxinluo Capsule in treatment of coronary heart disease (通心络胶囊治疗冠心病的疗效及机制研究). Chinese Journal of Evidence-Based Cardiovascular Medicine, 2018, 10(06): 729-732.

[66] Yang LG, Wen Y, Yang WX. Observation of the clinical effect of Tongxinluo capsule in the treatment of female patients with microvascular angina (通心络胶囊治疗女性微血管性心绞痛患者的临床效果观察). Chinese Journal of Primary Medicine and Pharmacy, 2018, 25(4): 413-415.

[67] Deng YJ, Zhang WJ, Tan XH. Effect of Tongxinluo capsule on myocardial enzyme and electrocardiography in the treatment of myocardial ischemia of coronary heart disease (通心络胶囊对冠心病心肌缺血的影响). Journal of Clinical Eletrocardiology, 2017, 26(3): 192-194.

[68] Xue XC, Wang YM. Clinical effect of betaloc combined with Tongxinluo capsule in treatment of angina pectoris of coronary heart disease (倍他乐克联合通心络胶囊治疗冠心病心绞痛的临床效果). Clinical Research and Practice, 2017,2(16):26-27.

[69] Pan LL, Zhang Y. Clinical study of betaloc and Tongxinluo capsule in treating coronary heart disease with angina pectoris (倍他乐克联合通心络胶囊治疗冠心病心绞痛临床研究). Electronic Journal of Clinical Pharmaceutical Literature, 2017, 4(54): 10637-10638.

[70] Sun WH, Lai SM, Bi XM, et al. The efficacy of Tongxinluo combined with metoprolol in the treatment of elderly coronary heart disease and its effect on vascular endothelial function (通心络联合美托洛尔治疗老年冠心病的疗效及对血管内皮功能的影响). World Latest Medicine Information, 2017,17(47):77-81.

[71] Ji LF, Wan XF. Clinical analysis of Tongxinluo capsule in the treatment of chest paralysis, qi deficiency and blood stasis syndrome (通心络胶囊治疗胸痹气虚血淤证的临床分析). The Journal of Medical Theory and Practice, 2017,30(21):3168-3169.

[72] Liu XL, Qin JW. The Clinical Effect of Tongxinluo Combined with Western Medicine in Treatment of Coronary Heart Disease Angina Pectoris (冠心病心绞痛采取通心络联合西药治疗的临床效果). Smart Health, 2017, 3(13): 7-9.

[73] Yang W, Sun F, Xia XH, et al. Effect of combined treatment with betalactam and Tongxinluo on

inflammatory factors, cardiac function and quality of life in patients with angina pectoris (倍他乐克与通心络联合治疗对心绞痛患者炎性因子、心功能和生活质量的影响). Chinese Journal of Practical Medicine, 2017, 44(24): 119-121.
[74] Pan JX, Zhang MX, Zheng JK, et al. Effect of Tongxinluo capsule combined atorvastatin on blood lipids and inflammatory factor in patient with coronary heart disease (通心络胶囊联合阿托伐他汀与单用阿托伐他汀对冠心病患者血脂、炎症因子水平的影响比较). Chinese Journal of General Practice, 2017, 15(3): 452-453.
[75] Yang JX, Yu XY. The safety and efficacy of the Tongxinluo capsule plus trimetazidine on myocardial ischemia (通心络胶囊联合曲美他嗪治疗心肌缺血的疗效及安全性观察). Clinical Journal of Chinese Medicine, 2017, 9(35): 91-92.
[76] Li J, Wei F, Tao JS, et al. Clinical efficacy analysis of rosuvastatin combined with Tongxinluo capsule in the treatment of hypertension complicated with coronary heart disease (瑞舒伐他汀联合通心络胶囊治疗高血压合并冠心病的临床疗效分析). Guizhou Medical Journal, 2017, 41(09): 951-953.
[77] Shi XY, Zhang JX. The curative effect and the influence on patient's hemorheology of Metoprolol and Tongxinluo Capsule in the treatment of Heart Failure of Coronary Heart Disease (美托洛尔与通心络胶囊联合治疗冠心病心力衰竭疗效及对患者血液流变学影响). Journal of Diseases Monitor & Control, 2016, 10(10): 793-795.
[78] Yang MW, He G, Wang D, et al. Effect of Tongxinluo Capsule and Isosorbide Mononitrate in Treatment of Angina Pectoris of Coronary Heart Disease (通心络胶囊与硝酸异山梨醇酯治疗冠心病心绞痛疗效体会). Journal of Liaoning University of Traditional Chinese Medicine, 2016, 18(12): 198-200.
[79] Deng LL, Cheng XX. Pharmacological Analysis of Influence of Tongxinluo Capsule Combined with Atorvastatin on Blood Lipid and Inflammatory Factor in Patients with Coronary Heart Disease (通心络胶囊联合阿托伐他汀钙片对冠心病患者血脂及炎症因子影响药理分析). Journal of Liaoning University of Traditional Chinese Medicine, 2016, 18(09): 190-192.
[80] Chen ZY, Liu H, Liu CL, et al. Observation of the efficacy of Tongxinluo capsule combined with clopidogrel in the treatment of angina pectoris in coronary heart disease (通心络胶囊联合氯吡格雷治疗冠心病心绞痛疗效观察). Journal of clinical rational drug use, 2016, 9(34): 38-39.
[81] Li XS, Zhu XM. Clinical study of Tongxinluo capsule in the treatment of coronary heart disease angina pectoris with hyperhomocysteinemia (通心络胶囊治疗冠心病心绞痛伴高同型半胱氨酸血症临床研究). clinical medicine, 2016, 36(12): 124-125.
[82] Liu DJ, Liu XF, Wang P, et al. Clinical pharmacist analysis of the efficacy of Tongxinluo Capsule in the treatment of angina pectoris of coronary heart disease (临床药师对通心络胶囊治疗冠心病心绞痛疗效分析). Nei Mongol Journal of Traditional Chinese Medicine, 2016, 35(13): 14-15.
[83] Wang SH, Wang ZL. Observation of the efficacy of trimetazidine combined with Tongxinluo capsule in the treatment of angina pectoris of coronary heart disease (曲美他嗪联合通心络胶囊治疗冠心病心绞痛疗效观察). Shaanxi Journal of Traditional Chinese Medicine, 2016, 37(02): 169-170.
[84] Zhao SY, Huang ZS, Wen J, et al. Clinical Effect of Tongxinluo Capsule Combined with Atorvastatin on Angina Pectoris in Patients with Coronary Heart Disease (通心络胶囊联合阿托伐他汀治疗冠心病心绞痛的临床疗效观察). Practical Journal of Cardiac Cerebral Pneumal and Vascular Disease, 2016, 24(8): 96-99.
[85] Li L, Cao XB, Wang JP, et al. Clinical observation of Tongxinluo Capsules combined with esmolol in treatment of coronary heart disease with myocardial ischemia (通心络胶囊联合艾司洛尔治疗冠心病心肌缺血的疗效观察). Drugs & Clinic, 2016, 31(07): 1012-1015.
[86] Zhong WJ, Feng XQ, Xie QX. Feasibility and safety study of metoprolol combined with Tongxinluo in the treatment of coronary heart disease and heart failure (美托洛尔联合通心络治疗冠心病心力衰竭的可行性及安全性研究). Modern Diagnosis and Treatment, 2016, 27(02): 222-223.
[87] Guo YQ, Zhen YF, Shi Y. Observation of the efficacy of Tongxinluo capsule combined with statins in the treatment of coronary heart disease angina (通心络胶囊联合他汀类药物治疗冠心病心绞痛疗效观察).

Modern Journal of Integrated Traditional Chinese and Western Medicine, 2016, 25(10): 1115-1117.
[88] Liu L, Han Y, Wang XH. Observation of the clinical efficacy of Tongxinluo capsule in the treatment of angina pectoris of coronary heart disease (通心络胶囊治疗冠心病心绞痛临床疗效观察). Health and Wellness Guide, 2016(29): 132.
[89] Wang SM, Tian XL. Analysis of the efficacy of Tongxinluo Capsule combined with Western medicine in the treatment of angina pectoris of coronary heart disease (通心络胶囊配合西药治疗冠心病心绞痛的疗效分析). Chinese Journal of Critical Care Medicine, 2016, 36(z1).
[90] Yuan F, Luo XP. Effect of Tongxinluo Capsule Combined with Metoprolol Sustained-release Tablets on Vascular Endothelial Function and Cardiac Function Indexes of Coronary Heart Disease (通心络胶囊联合美托洛尔缓释片对冠心病血管内皮功能和心功能指标的影响). Chinese Journal of Prevention and Control of Chronic Diseases, 2016, 24(04): 300-302.
[91] Hang L, Tian HJ, Li J. Effect of atorvastatin combined with Tongxinluo Capsule on cTnI, HbA1c and clinical efficacy in patients with coronary heart disease (阿托伐他汀联合通心络胶囊对冠心病病人 cTnI、HbA1c 及临床疗效的影响). Chinese Journal of Integrative Medicine on Cardio-/Cerebrovascular Disease, 2016, 14(15): 1765-1767.
[92] Liu SG, An J. Clinical effect of Atorvastatin Combined with Tong Xin Luo capsule in treatment of unstable angina pectoris of coronary heart disease (阿托伐他汀联合通心络胶囊治疗冠心病不稳定型心绞痛的临床效果观察). Journal of clinical rational drug use, 2016, 9(05): 3-4.
[93] Liu B, Yang N. Clinical observation of Tongxinluo Capsules combined with indapamide in treatment of hypertension with coronary heart disease (通心络胶囊联合吲达帕胺治疗高血压合并冠心病的疗效观察). Drugs & Clinic, 2016, 31(08): 1160-1163.
[94] Tian SC, Wang YF, Zhou JP. Observation of the clinical efficacy of Tongxinluo combined with nitroglycerin in the treatment of angina pectoris in coronary heart disease (通心络联合硝酸甘油治疗冠心病心绞痛的临床疗效观察). Healthy People, 2015, 9(02): 139-140.
[95] Ding ML, Chen L, Gong YY. Tongxinluo up-regulates CXCR4 signaling pathway and enhances the function of vascular endothelial progenitor cells in patients with coronary heart disease (通心络上调 CXCR4 信号通路增强冠心病患者血管内皮祖细胞功能). Guangdong Medical Journal, 2015, 36(24): 3862-3865.
[96] He BQ, Fan CY, Chen LF. Observation of the clinical effect of Tongxinluo capsule in the treatment of angina pectoris of coronary heart disease (通心络胶囊治疗冠心病心绞痛的临床效果观察). Strait Pharmaceutical Journal, 2015, 27(12): 184-185.
[97] Zhang P, Cao YJ, Zhang MD. Effect of Tongxinluo combined with clopidogrel on the clinical symptoms and hemodynamics of angina pectoris in coronary heart disease (通心络联合氯吡格雷治疗方案对冠心病心绞痛临床症状及血流动力学的影响). World Clinical Medicine, 2015(10): 162-163.
[98] Miao H, Huang HS. Clinical observation of Tongxinluo capsule in the treatment of angina pectoris in elderly patients with coronary heart disease (通心络胶囊治疗老年冠心病心绞痛 90 例临床观察). World Latest Medicine Information, 2015, 15(18): 70-71.
[99] Liang Y, You D, Ao XJ. Observation of the clinical efficacy of Tongxinluo in the treatment of angina pectoris in 42 cases of coronary heart disease (通心络治疗 42 例冠心病心绞痛的临床疗效观察). Modern Diagnosis and Treatment, 2015, 26(03): 660-661.
[100] Jian LP, Wang L, Wu D. Clinical control observation of the effect of Tongxinluo capsule on the treatment of coronary heart disease (通心络胶囊对冠心病治疗效果的临床对照观察). China Health Care Nutrition, 2015,25(17):286.
[101] Lin W, Yang HY. Effect of metoprolol combined with Tongxinluo on serum hypersensitive C-reactive protein and plasma N-terminal B-type natriuretic peptidinogen levels in patients with coronary heart disease cardiac insufficiency (美托洛尔联合通心络对冠心病心功能不全患者血清超敏 C 反应蛋白及血浆 N 末端 B 型钠尿肽原水平的影响). Chinese Journal of Primary Medicine and Pharmacy, 2015, 0(21): 3280-3283.

[102] Ma WJ, Wang HY. Effect of Tongxinluo combined with clopidogrel on the clinical symptoms and hemodynamics of angina pectoris of coronary heart disease (通心络联合氯吡格雷治疗对冠心病心绞痛临床症状及血流动力学的影响). Chinese Journal of Gerontology, 2015, 35(05): 1197-1199.

[103] Zhou XH, Cai JP, Guo LS, et al. Study on the therapeutic effect of Tongxinluo capsule on coronary microvascular angina (通心络胶囊对冠状微血管性心绞痛的治疗效果研究). Chinese Journal of Practical Medicine, 2015, 42(19): 69-70.

[104] Li XJ, Hou JJ. Analysis of the efficacy of metoprolol combined with Tongxinluo capsule in the treatment of premature beats in coronary heart disease (美托洛尔联合通心络胶囊治疗冠心病早搏的疗效分析). China Practical Medicine, 2015, 10(30): 117-118.

[105] Wu ZH, Chen YP, Lei CJ. Analysis on Efficacy an d Safety of Metoprolol Combined with Nicorandil for Heart Failure of Coronary Heart Disease (美托洛尔联合通心络治疗冠心病心力衰竭患者的可行性及安全性). China Journal of Pharmaceutical Economics, 2015, 10(7): 69-70.

[106] Zhang ZW, Ye HY. Tongxinluo combined with clopidogrel in the treatment of coronary heart disease angina pectoris 45 cases of efficacy observation (通心络联合氯吡格雷治疗冠心病心绞痛 45 例疗效观察). Guiding Journal of Traditional Chinese Medicine and Pharmacy, 2015, 21(05): 69-71.

[107] Huang C, Ekbar A. TXL combined with Western Intervention in Coronary Heart Randomized Controlled Study (通心络联合西药治疗冠心病血脂随机平行对照研究). Journal of Practical Traditional Chinese Internal Medicine, 2015, 29(06): 121-123.

[108] Jin D, Xia Y. Clinical analysis of Tongxinluo capsule in the treatment of angina pectoris in coronary heart disease (通心络胶囊治疗冠心病心绞痛的临床分析). Biotech World, 2015(01): 65.

[109] He LX, Li WD. The Effect of Tongxinluo with Atorvastatin on Blood Lipids in Patients with Coronary Heart Disease (通心络胶囊联合阿托伐他汀对冠心病患者血脂的影响). World Chinese Medicine, 2015, 10(09): 1344-1346.

[110] Fu R, Wu J. Analysis of lipid-lowering efficacy of Chinese medicine Tongxinluo on patients with angina pectoris of hyperlipidemic coronary heart disease (中药通心络对高血脂性冠心病心绞痛患者降脂疗效分析). China Health Care Nutrition, 2015, 25(17): 377.

[111] Sun SJ, Zhang P, Li WZ, et al. Clinical efficacy of Tongxinluo combined with aspirin in the treatment of unstable angina pectoris (通心络联合阿司匹林治疗不稳定型心绞痛的临床疗效观察). Acta Chinese Medicine and Pharmacology, 2015, 43(03): 126-128.

[112] Li XC, Yan PX, Deng FJ, et al. Evaluation on the Efficacy and Safety of Tongxinluo capsule in the Treatment of Coronary Heart Disease (通心络胶囊治疗冠心病 60 例疗效及安全性评价). Clinical Medicine & Engineering, 2014, 21(09): 1149-1150.

[113] Cheng ZY, Wu ZG. Clinical efficacy observation of 52 cases of Tongxinluo capsule in the treatment of angina pectoris of coronary heart disease (通心络胶囊治疗冠心病心绞痛的 52 例临床疗效观察). Nei Mongol Journal of Traditional Chinese Medicine, 2014, 33(21): 2-3.

[114] Wang LN, Wang YY. Observation on the efficacy of Tongxinluo capsule in the treatment of angina pectoris of coronary heart disease (通心络胶囊治疗冠心病心绞痛疗效观察). Shaanxi Journal of Traditional Chinese Medicine, 2014, 35(04): 424-425.

[115] Chen XZ, Zhang YY. Observation on the efficacy of Tongxinluo capsule with western medicine in the treatment of angina pectoris of coronary heart disease (通心络胶囊配合西药治疗冠心病心绞痛疗效观察). Shaanxi Journal of Traditional Chinese Medicine, 2014, 35(08): 980-982.

[116] Shu LL, Feng DQ, Li L. Western medicine supplemented with Tongxinluo capsule in the treatment of 80 cases of angina pectoris of coronary heart disease in the elderly (西药辅以通心络胶囊治疗老年冠心病心绞痛 80 例). Medical Information, 2014, 0(29): 78-79.

[117] Li N, Gu XJ, Zheng XY, et al. Clinical observation the therapeutic effect of Tongxinluo capsule on patients with angina pectoris and depression (通心络辅治冠心病心绞痛伴抑郁症状患者的临床观察).

Chinese Journal of Difficult and Complicated Cases, 2014, 13(11): 1168-1170.

[118] Xie ZH, Fang DL, Liu QW. Comparison of the efficacy of metoprolol alone and in combination with Tongxinluo in the treatment of coronary heart failure (美托洛尔单用和联合通心络治疗冠心病心力衰竭疗效比较). Chinese Journal of Practical Medicine, 2014, 41(7): 107-108.

[119] Meng TY, Liu HF, Liu L, et al. Tongxinluo combined with clopidogrel for the treatment of angina pectoris in coronary heart disease (通心络联合氯吡格雷治疗冠心病心绞痛疗效观察). China Health Industry, 2014, 11(03): 110-112.

[120] Cui WH, Ma JF. Tongxinluo capsule combined isosorbide mononitrate tablets in treatment of elderly patients with angina pectoris (通心络联合单硝酸异山梨酯治疗老年冠心病心绞痛的临床研究). Modern Chinese Doctor, 2014, 52(12): 64-65.

[121] Yu HF, Cheng ZF. Effect of Metoprolol Combined with Tongxinluo Capsule on Heart Function of Patients with Coronary Heart Disease and Heart Failure (美托洛尔联合通心络胶囊对冠心病心力衰竭患者心功能的影响). Chinese Archives of Traditional Chinese Medicine, 2014, 32(09): 2286-2288.

[122] Hao DJ, Li JH. Efficacy of Tongxinluo capsule in the treatment of angina pectoris of coronary heart disease and its effect on blood lipids (通心络胶囊治疗冠心病心绞痛的疗效及对血脂的影响). Chinese Journal of Integrative Medicine on Cardio-/Cerebrovascular Disease, 2014, 12(04): 408-409.

[123] Zheng L, Liu DY, Lu H. Tongxinluo combined with clopidogrel for the treatment of angina pectoris in coronary heart disease (通心络联合氯吡格雷治疗冠心病心绞痛疗效观察). Chinese Journal of Information on Traditional Chinese Medicine, 2014, 31(06): 90-92.

[124] Guo J, Li ZM. Tongxinluo on Coronary Heart Disease with Abnormal Blood Pressure Morning Surge Associated Myocardial Ischemia (通心络对冠心病合并高血压晨峰异常心肌缺血的影响). Heilongjiang Medicine Journal, 2014, 27(02): 287-290.

[125] Shen YZ, Gao P. Observation on the clinical efficacy of Tongxinluo capsule on elderly patients with coronary heart disease angina pectoris (通心络胶囊对老年冠心病心绞痛患者的临床疗效观察). Prevention and Treatment of Cardio-Cerebral-Vascular Disease, 2014, 14(06): 523-525.

[126] Hong ZZ, Huang LR. The effect of Tongxinluo combined with clopidogrel therapy on angina pectoris of coronary heart disease and the effect on blood rheology (通心络联合氯吡格雷治疗对冠心病心绞痛的效果及对血液流变学的影响). Journal of Bethune Military Medical College, 2014, 12(03): 297-298.

[127] Zhang XS, Liu ZS, Zhou JM. Effects of Tongxinluo capsule on lipid metabolism and blood rheology in patients with unstable angina pectoris (通心络胶囊对不稳定型心绞痛患者脂代谢及血液流变性的影响). Journal of New Chinese Medicine, 2014, 46(03): 45-47.

[128] Cui CB, Cheng H. The effect of Tongxinluo capsule combined with copidogrel hdrogen slfate on vascular endothelial function and inflammatory factors in the patients with unstable angina pectoris (通心络联合氯吡格雷对不稳定型心绞痛患者血管内皮功能和炎性因子的影响). Chinese Journal of Difficult and Complicated Cases, 2014, 13(09): 936-938.

[129] Zhou Q, Shi WL, Zhang YY, et al. Efficacy and safety of Tongxinluo capsule on angina pectoris of coronary heart disease in the elderly (通心络胶囊对老年冠心病心绞痛的疗效及安全性). Medical Aesthetics and Beauty, 2013, 21(1): 29-30.

[130] Zhang H, Gao YC. The efficacy of metoprolol combined with Tongxinluo in the treatment of coronary heart failure (美托洛尔联合通心络治疗冠心病心力衰竭的疗效观察). China Health Care Nutrition, 2013, 23(12): 7516.

[131] Shi CJ, Cai SL, Zhang JD. Treatment of coronary heart disease and angina pectoris with Tongxinluojiaonang (通心络胶囊治疗冠心病心绞痛疗效观察). Chinese Journal of New Clinical Medicine, 2013, 6(06): 544-546.

[132] Liang XD, Wang QX. To observe the curative effect of Tongxinluo capsule combined with amiodarone in the treatment of coronary heart disease complicated with frequent ventricular premature

beat (通心络胶囊联合胺碘酮治疗冠心病并发频发室性早博的疗效观察). China Healthcare Innovation, 2013, 8(21): 14-15.

[133] Ma HR, Zhang Y, Jia Y. Tongxinluo capsule combined with trimetazidine in the treatment of coronary heart disease in 30 cases (通心络胶囊联合曲美他嗪治疗冠心病 30 例). Chinese Journal of Gerontology, 2013, 33(12): 2873-2874.

[134] Luo LH, Long JL. Clinical efficacy of atorvastatin and Tongxinluo capsule in the treatment of coronary heart disease (阿托伐他汀和通心络胶囊治疗冠心病的临床疗效). Contemporary Medicine, 2013, 19(07): 147-148.

[135] Ding B, Fang ZG, Ren AL. Therapeutic effect of Tongxinluo capsule on unstable angina pectoris (通心络胶囊治疗不稳定型心绞痛疗效观察). Journal of Medical Forum, 2013, 34(09): 25-27.

[136] Arziguri K, Gurinisha W. Clinical analysis of Tongxinluo capsule in treating 100 cases of unstable angina combined with hyperlipidemia (通心络胶囊治疗 100 例不稳定型心绞痛合并高脂血症的临床分析). Chinese Community Physician, 2013, 15(07): 216.

[137] Wang G, Li JP. Effect of Tongxinluo capsule on serum IL-18 and hs-CRP in elderly patients with coronary heart disease angina pectoris (通心络胶囊对老年冠心病心绞痛患者血清 IL-18 和 hs-CRP 的影响). Guangdong Medical Journal, 2012, 33(02): 276-278.

[138] Kuai ZP, Gong QY, Yan JJ, et al. Effects of Tongxinluo capsule on inflammatory markers and carotid arterial plaque in patients with coronary artery disease (通心络胶囊对冠心病患者血清 Visfatin 和 IL-6 表达及颈动脉粥样斑块的影响). Jiangsu Medical Journal, 2012, 38(08): 904-906.

[139] Fan J, Yan WL, Gao YM, et al. Clinical observation of Tongxinluo capsule in the treatment of angina pectoris of coronary heart disease (通心络胶囊治疗冠心病心绞痛的临床观察). Nei Mongol Journal of Traditional Chinese Medicine, 2012, 31(05): 11.

[140] Zhao YD, Ni Y. Clinical efficacy observation of Tongxinluo capsule in treating 45 cases of coronary heart disease (通心络胶囊治疗冠心病 45 例临床疗效观察). Shandong Journal of Traditional Chinese Medicine, 2012, 31(05): 321-323.

[141] Tian J, Xi LY, Chen DF. The efficacy of metoprolol combined with Tongxinluo capsule in the treatment of premature beats in coronary heart disease (美托洛尔联合通心络胶囊治疗冠心病早搏疗效观察). Modern Journal of Integrated Traditional Chinese and Western Medicine, 2012, 21(11): 1214-1217.

[142] Huang YM, Tang SQ, Huang YH. Tongxinluo capsule treatment of coronary heart disease angina pectoris 40 cases efficacy observation (通心络胶囊治疗冠心病心绞痛 40 例疗效观察). Medical Journal of Chinese People's Health, 2012, 24(16): 1977-1978.

[143] Shi Q, Sun ZG. Tongxinluo capsule treatment of angina pectoris clinical analysis (通心络胶囊治疗冠心病心绞痛临床疗效分析). China Medicine and Pharmacy, 2012, 2(24): 75-76.

[144] Zhu HY, Zhou LY. Tongxinluo in the treatment of angina pectoris of coronary heart disease, 46 cases (通心络治疗冠心病心绞痛 46 例). Chinese Medicine Modern Distance Education of China, 2012, 10(18): 21-22.

[145] Song YM, Ding YQ. Clinical effects of atorvastatin plus Tong Xin Luo capsule on treating coronary heart disease (阿托伐他汀和通心络胶囊治疗冠心病的临床疗效). Clinical Journal of Chinese Medicine, 2012, 4(06): 59-60.

[146] Sun L, Liu XJ, Zhang P. Effect observation on treating 26 cases of angina pectoris patients by Tongxinluo capsule (通心络胶囊治疗冠心病心绞痛 26 例疗效观察). China Medicine and Pharmacy, 2012, 2(14): 47-48.

[147] Yao MC, Zhou JH. Effect of Tongxinluo capsule on hemodynamics in patients with unstable angina pectoris (通心络胶囊对不稳定型心绞痛患者血流动力学的影响). Clinical Medicine, 2012, 32(10): 118-119.

[148] Wang Q, Du B. The effect of Tongxinluo capsule on matrix metalloproteinase-9(MMP-9)and high sensitivity C-reactive protein(hs-CRP)in patients with unstable angina (通心络胶囊对不稳定心绞痛患者血

清基质金属蛋白酶-9 和高敏 C-反应蛋白的影响). Journal of Community Medicine, 2012, 10(12): 41-43.

[149] Yu YS, Hu N. Analysis of the efficacy of betalactam combined with Tongxinluo capsule in the treatment of unstable angina pectoris (倍他乐克联合通心络胶囊治疗不稳定性心绞痛疗效分析). Chinese Medical Innovations, 2012, 9(35): 17-18.

[150] Chen YB, Li HP, Cai ZL, et al. A research on curative effect of Tongxinluo capsule on patients with unstable angina and its Mechanism (通心络胶囊对不稳定型心绞痛患者的疗效及其机制研究). Chinese Journal of Integrated Traditional and Western Medicine in Intensive and Critical Care, 2012, 19(6): 336-339.

[151] Liang YM, Zhang SC, Chen LS. Effect of Tongxinluo capsule on serum inflammatory factors in patients with coronary heart disease (通心络胶囊对冠心病患者血清炎性因子的影响). Hebei Medicine, 2011, 17(02): 217-219.

[152] Wang FL, Guo YH. Effect observation of Tongxinluo capsule in the treatment of angina pectoris of coronary heart disease (通心络胶囊治疗冠心病心绞痛疗效观察). Journal of clinical rational drug use, 2011, 4(12): 8-9.

[153] Xu J, Song XR. Effect and care of Tongxinluo capsule in the treatment of angina pectoris of coronary heart disease in 60 cases of elderly people (通心络胶囊治疗 60 例老年冠心病心绞痛的效果及护理). Chinese General Practice Nursing, 2011, 9(02): 132-133.

[154] Jiang FY, Zhuo LA, Zhang LC, et al. Effects of Tongxinluo on serum level of adiponectin and blood lipid in patients with coronary heart disease (通心络对冠心病患者血清脂联素及血脂的影响). Chinese Journal of Difficult and Complicated Cases, 2011, 10(01): 10-12.

[155] Li SY, Dong CY, Dong CM. Observation on the effect of Tongxinluo in the treatment of angina pectoris of coronary heart disease (通心络治疗冠心病心绞痛的效果观察). China Foreign Medical Treatment, 2011, 30(10): 117.

[156] Li J, Li XY, Zhang L. Analysis of the efficacy of Tongxinluo capsule in the treatment of coronary heart disease chronic heart failure (通心络胶囊治疗冠心病慢性心力衰竭疗效分析). Hebei medical journal, 2011, 33(12): 1892-1893.

[157] Qin JH, Zhang GQ. Efficacy observation of Tongxinluo Capsule in treatment of angina pectoris (通心络胶囊治疗冠心病心绞痛疗效观察). Journal of Modern Medicine & Health, 2011, 27(03): 329-330.

[158] Li XD, Yao HB. Observation on the effect of Tongxinluo capsule in treating angina pectoris of coronary heart disease to improve blood lipid and blood rheology (通心络胶囊治疗冠心病心绞痛改善血脂、血流变作用观察). Chinese Community Physician, 2011, 27(04): 11.

[159] Gao YC, Guo JL, Wang S. Observations on 92 cases of coronary heart disease angina pectoris treated with Tongxinluo capsule (通心络胶囊治疗冠心病心绞痛 92 例观察). Chinese Community Physician, 2011, 27(05): 11.

[160] Qi JQ, Ma JE, Bai RL. Observation on the efficacy of Tongxinluo capsule in the treatment of angina pectoris of coronary heart disease (通心络胶囊治疗冠心病心绞痛疗效观察). Chinese And Foreign Medical Research, 2011, 9(12): 22.

[161] Ma JY, Liu LL, Ru C. Clinical discussion on the treatment of unstable angina pectoris with Tongxinluo capsule (通心络胶囊治疗不稳定性心绞痛的临床探讨). Jilin Medical Journal, 2011, 32(11): 2154-2155.

[162] Ma YL, Yu YH. Clinical observation on the treatment of unstable angina pectoris with Tongxinluo capsule (通心络胶囊治疗不稳定型心绞痛的临床观察). Qinghai Medicine Magazine, 2011, 41(07): 71-72.

[163] Chen JF, Xu HC. Clinical observation of Tongxinluo combined with isosorbide mononitrate in the treatment of unstable angina pectoris (通心络联合单硝酸异山梨酯治疗不稳定型心绞痛的临床观察). Capital Medicine, 2011, 18(12): 39.

[164] Zhan YM, Zhu F, Xu XP. Clinical observation of Tongxinluo in the treatment of unstable angina pectoris (通心络治疗不稳定型心绞痛临床观察). Modern Journal of Integrated Traditional Chinese and Western Medicine, 2011, 20(11): 1331-1332.

[165] Ge XJ, Guan ZL. EFFECTS OF TONG-XIN-LUO ON VASCULAR ENDOTHELIAL IN PATIENTS WITH CORONARY ARTERY DISEASE (通心络胶囊对冠心病患者血管内皮功能的影响). Chinese Journal of Coal Industry Medicine, 2011, 14(11): 1583-1586.

[166] Chen YH, Xu Y, Cui YT. Clinical Value of Tongxinluo Capsule for Treating Unstable Angina Complicating Diabetes in 60 Elderly Cases (通心络胶囊治疗不稳定型心绞痛合并糖尿病老年患者 60 例). China Pharmaceuticals, 2011, 20(24): 84-85.

[167] Lu YG, Fu CH, Yan H, et al. Effect of Tongxinluo on endothelial micro—particles in patients with unstable angina (通心络对不稳定型心绞痛患者循环内皮微粒的影响). Chinese Journal of Integrated Traditional and Western Medicine in Intensive and Critical Care, 2011, 18(1): 41-43.

[168] Kou JH, Ren HY, Wu X, et al. Effects of the Short-term High-dose Tongxinluo on QT Dispersion in Patients with Coronary Heart Disease (短期大剂量通心络对冠心病患者 QT 离散度的影响). Hebei Medicine, 2010, 16(09): 1055-1057.

[169] Zhou Q, Qian BW. Clinical efficacy observation of Tongxinluo auxiliary treatment of angina pectoris in coronary heart disease (通心络辅助治疗冠心病心绞痛的临床疗效观察). Chinese Journal of Difficult and Complicated Cases, 2010, 9(05): 355-356.

[170] Qi W, Qi H. Clinical observation on 64 cases of coronary heart disease angina treated with Tongxinluo capsule (通心络胶囊治疗冠心病心绞痛 64 例临床观察). Chinese Journal of Ethnomedicine and Ethnopharmacy, 2010, 19(24): 127-128.

[171] Gu JX, Yang F, Shao ZB, et al. Clinical observation on 41 cases of myocardial ischemia with gas deficiency and blood stasis treated with Tongxinluo capsule (通心络胶囊治疗心肌缺血气虚血瘀证 41 例临床观察). Clinical Journal of Traditional Chinese Medicine, 2010, 22(12): 1047-1048.

[172] Wang SX, Mou YG, Liu TX, et al. Efficacy of Tong-Xin-Lo capsule in the treatment of unstable angina pectoris serum and its effect on serum matrix metalloproteinases-2 (通心络胶囊治疗不稳定型心绞痛患者疗效观察及其对血清 MMP-2 的影响). Hainan Medical Journal, 2010, 21(15): 34-35.

[173] Zhao YW, Niu YG. Observation on the efficacy of Tongxinluo capsule in the treatment of unstable angina pectoris (通心络胶囊治疗不稳定性心绞痛的疗效观察). Chinese Community Physician, 2010, 12(20): 141-142.

[174] Tao P, Liu EX. Analysis of 36 cases of unstable angina pectoris treated with Tongxinluo capsule (通心络胶囊治疗不稳定型心绞痛 36 例分析). Modern Chinese Doctor, 2010, 48(27): 116-122.

[175] Wang XQ. Yang NR. Analysis of clinical efficacy of Tongxinluo capsule in the treatment of unstable angina pectoris (通心络胶囊治疗不稳定型心绞痛临床疗效分析). China Foreign Medical Treatment, 2010, 29(24): 114.

[176] Song YX, Cai Q. Effect of Tongxinluo on C-reactive protein and blood rheology indexes in patients with coronary heart disease (通心络对冠心病患者 C 反应蛋白及血液流变学指标的影响). Gansu Journal of Traditional Chinese Medicine, 2009, 22(02): 30-31.

[177] Niu YJ, Dai GW, Zheng FY. Effect of Tongxinluo capsule on ankle-arm index in patients with coronary heart disease (通心络胶囊对冠心病患者踝臂指数的影响). Nei Mongol Journal of Traditional Chinese Medicine, 2009, 28(04): 2-3.

[178] Wang WZ, Luo YC, Fang LJ. Clinical efficacy observation of Tongxinluo capsule in the treatment of angina pectoris of coronary heart disease (通心络胶囊治疗冠心病心绞痛临床疗效观察). Journal of Community Medicine, 2009, 7(23): 15.

[179] Zhang LX, Li CX. Clinical observation on 90 cases of coronary heart disease angina treated by Tongxinluo capsule (通心络胶囊治疗冠心病心绞痛 90 例临床观察). Journal of Hebei Medical College for Continuing Education, 2009, 26(02): 62-63.

[180] Qi YX, Huang P. Observation on the efficacy of Tongxinluo in the treatment of coronary heart disease (通心络治疗冠心病疗效观察). Chronic Pathematology Journal, 2009, 11(01): 31-32.

[181] Sun XJ, Pan W. Effect of Tongxinluo capsule on cardiac function in patients with coronary heart disease (通心络胶囊对冠心病患者心功能的影响). Chinese Community Physician, 2009, 11(20): 144.

[182] Chen MY, Xu JX. Efficiency and feasibmty of tongxinluo capsule treatment wim angina caused by coronary heart disease. Clinical efficacy observation of Tongxinluo capsule in the treatment of angina pectoris of coronary heart disease (通心络胶囊治疗冠心病心绞痛临床疗效观察). Chinese Journal of Modern Drug Application, 2009, 3(01): 9-10.

[183] Wu LY, Zhang WX. Observation of TongXinLuo Capsule on 184 Patients with Coronary Heart Disease (通心络胶囊治疗老年冠心病心绞痛 184 例观察). Guide of China Medicine, 2009, 7(10): 321-322.

[184] Suo L, Sun CH. Comparison of Tongxinluo and Lunanxinkang in the treatment of angina pectoris alone or in combination (通心络和鲁南欣康单用或合用治疗心绞痛的比较). Guide of China Medicine, 2009, 7(12): 305-306.

[185] Sun LD, Leng J. Tongxinluo treatment of coronary heart disease heart failure efficacy observation (通心络治疗冠心病心力衰竭疗效观察). China Foreign Medical Treatment, 2009, 28(26): 71-73.

[186] Ha XM, Zhang WH. Observation on the efficacy of Tongxinluo capsule in the treatment of angina pectoris of coronary heart disease (通心络胶囊治疗冠心病心绞痛疗效观察). Jilin Medical Journal, 2009, 30(22): 2798-2799.

[187] Guo X, Wang L, Yuan JF. Effects of Naoxintong Capsule and Tongxinluo Capsule on Silent Myocardial Ischemia (脑心通和通心络对无症状心肌缺血的影响). Lishizhen Medicine and Materia Medica Research, 2009,20(02):466-467.

[188] Yi SH, Zhang H, Song JM. Effect of Tongxinluo capsule on thromboxane B_2 and lipid changes in coronary heart failure (通心络胶囊对冠心病心力衰竭血栓素 B_2 及脂质变化的影响). Chinese Journal of Misdiagnostics, 2009, 9(30): 7378-7379.

[189] Wu G, Li SR. Analysis of the efficacy of Tongxinluo capsule in the treatment of angina pectoris (通心络胶囊治疗心绞痛疗效分析). Chinese Journal of Misdiagnostics, 2009, 9(28): 6889-6890.

[190] Wang X, Li J. Clinical efficacy observation of Tongxinluo capsule in the treatment of coronary heart disease (通心络胶囊治疗冠心病的临床疗效观察). Chinese Journal of Modern Drug Application, 2009, 3(22): 105-106.

[191] Li YS, Wu XQ. Tongxinluo and isosorbide mononitrate combination for the treatment of coronary heart disease angina pectoris in the elderly 60 cases efficacy observation (通心络与单硝酸异山梨酯合用治疗老年冠心病心绞痛 60 例疗效观察). Guide of China Medicine, 2009, 7(12): 278-279.

[192] Long M, Long Y, Chen XL. Effect of Tongxinluo capsule on blood lipids and serum high-sensitivity C-reactive protein in patients with unstable angina pectoris (通心络胶囊对不稳定性心绞痛患者血脂和血清高敏 C 反应蛋白的影响). Journal of the Fourth Military Medical University, 2009, 30(16): 1522.

[193] Wang XP, Gao MY, Deng W. The Efficacy of Tongxinluo Capsule on the Unstable Angina with Hyperlipidemia (通心络胶囊治疗不稳定型心绞痛合并高脂血症的疗效观察). Journal of Gannan Medical University, 2009, 29(03): 397-398.

[194] Kong XQ, Wang FL. Tongxinluo Cooperate Atorvastatin Unstable Angina Lipid-Lowering Effects and Endothelial Function (通心络与阿托伐他汀并用对不稳定型心绞痛降脂疗效及内皮功能的影响). Journal of Practical Traditional Chinese Internal Medicine, 2009, 23(11): 51-52.

[195] Zhang J, Li GW. The effects of Tongxinluo capsule in the treatment of 86 patients with unstable angina (通心络胶囊治疗不稳定性心绞痛的疗效观察). Medical Journal of West China, 2009, 21(08): 1352-1353.

[196] Liu T, Cao CY, Li W, et al. Effect of Tongxinluo on high-sensitivity C-reactive protein and P-selectin in elderly patients with unstable angina pectoris (通心络对高龄不稳定型心绞痛患者高敏 C 反应蛋白和 P 选择素的影响). Modern Journal of Integrated Traditional Chinese and Western Medicine, 2009, 18(33): 4062-4063.

[197] Yu N, Chen LF. Effect of Tongxinluo capsule on high-sensitivity C-reactive protein and P-selectin in senile patients with unstable angina (通心络胶囊治疗不稳定性心绞痛 68 例分析). China Modern Medicine, 2009, 16(13): 104-105.

[198] Guo J, Chen F, Guo YF, et al. Effcet of Tongxinluo capsul on hs-CRP、TNF-a in patients with angina pectoris (通心络胶囊对不稳定性心绞痛患者血清高敏 C 反应蛋白、肿瘤坏死因子-α 的影响). Journal of Chinese Microcirculation, 2009, 13(06): 535-537.

[199] Yao W, Yuan YM, Sun MJ. Tongxinluo capsule for the treatment of coronary heart disease angina combined with hyperlipidemia in 36 cases (通心络胶囊治疗冠心病心绞痛合并高脂血症 36 例). Shandong Journal of Traditional Chinese Medicine, 2009, 28(02): 99-100.

[200] Hou RT, Li SQ, Gao SM, et al. Effect of Tongxinluo on exercise tolerance and myocardial ischemia in patients with coronary heart disease (通心络对冠心病患者运动耐量和心肌缺血的影响). Chinese Journal of Difficult and Complicated Cases, 2008, 7(12): 731-732.

[201] Hu MQ, Peng ZQ, Huang AY. Clinical understand of Tongxinluo capsule in the treatment of angina pectoris (通心络胶囊治疗冠心病心绞痛 36 例观察). Chinese Journal of Modern Drug Application, 2008(05): 31-32.

[202] Su XY, Han ZX, Liang YM, et al. RCT study of Tongxinluo capsules on left ventricular function and angina of patients with primary hypertrophic cardiomyopathy (通心络胶囊对原发性肥厚型心肌病左室功能和心绞痛疗效影响的随机对照研究). Chinese Journal of Integrated Traditional and Western Medicine in Intensive and Critical Care, 2008(05): 286-288.

[203] Kang Y, Guo XY, Zhang TS, et al. Clinical observation on 32 cases of coronary heart disease treated with Tongxinluo capsule (通心络胶囊治疗冠心病 32 例临床观察). Shanxi Medical Journal, 2008(07): 598-599.

[204] Li LT, Lv J. Effect of Tongxinluo capsule on hyperlipidemia and hyperviscosity in patients with coronary heart disease (通心络胶囊对冠心病患者高血脂和高血粘度的影响). Chinese Journal of Microcirculation, 2008(03): 72.

[205] Wang X, Hu D Y, Sha O. Effect of compound paeonol dripping pill on levels of plasma inflammatory mediators in patients with unstable angina). Zhongguo Zhong Xi Yi Jie He Za Zhi, 2008, 28(5): 395-398.

[206] He YH, Yao CJ, Li G, et al. Effect of Tongxinluo on intercellular adhesion molecule-I in patients with unstable angina pectorls (通心络对不稳定性心绞痛患者 sICAM-1 水平的影响). Hainan Medical Journal, 2008(02): 10-11.

[207] Wang XG, Zhang HG. Clinical observation on 104 cases of unstable angina pectoris treated with Tongxinluo capsule combined with low molecular heparin (通心络胶囊联合低分子肝素治疗不稳定型心绞痛 104 例临床观察). Journal of Community Medicine, 2008(08): 20-21.

[208] Xin L, li JY, Wang Y, et al. Clinical observation on the treatment of unstable angina pectoris with Tongxinluo capsule (通心络胶囊治疗不稳定心绞痛临床观察). Practical Journal of Cardiac Cerebral Pneumal and Vascular Disease, 2008, 16(4): 256-259.

[209] Zhang LC, Zhang JJ. Clinical observation of Tongxinluo capsule in the treatment of unstable angina in coronary heart disease (通心络胶囊治疗冠心病不稳定型心绞痛临床观察). Practical Clinical Journal of Integrated Traditional Chinese and Western Medicine, 2008(03): 19.

[210] Lin YC, Liu H, Huang P, et al. The efficacy of Tongxinluo capsule combined with trimetazidine in the treatment of unstable angina pectoris in elderly people (通心络胶囊联合曲美他嗪治疗老年不稳定型心绞痛疗效观察). Journal of New Chinese Medicine, 2008(06): 33-34.

[211] Song K, Li XP, Xu GY. Therapeutic efficacy of Tongxinluo in treating unstable angina pectoris in the elderly (通心络治疗老年不稳定型心绞痛疗效观察). Journal of Changzhi Medical College, 2008(02): 109-110.

[212] Chao WN, Wan YH, Liu YP. Effect of Tongxinluo Capsule on C-reactive Protein in Patients with

Unstable Angina (通心络胶囊对不稳定性心绞痛患者 C 反应蛋白的影响). Chinese Journal of Hemorheology, 2008(03): 357-358.

[213] Jia Y, Sun X, Ge H. Effect of Tongxinluo Capsules on the Function of Vascular Endothelium in Patients with Unstable Angina Pectoris (通心络胶囊对不稳定型心绞痛血管内皮功能的影响). Journal of China Medical University, 2008(02): 285.

[214] Luo Y, Yin YZ. Effect of Tongxinluo Capsul on C-reactive protein in patients with unstable angina pectoris (通心络胶囊对不稳定型心绞痛的治疗作用和对 C 反应蛋白的影响). Chinese Medical Innovations, 2008,5(35): 18-19.

[215] Fan YH, Sun GR, Fan YQ. Clinical observation of Tongxinluo in the treatment of unstable angina pectoris (通心络治疗不稳定型心绞痛的临床观察). China Medical Herald, 2008(06): 73-74.

[216] Zhang YX, Chen GL. Effect of Tongxinluo on plasma endothelin in unstable angina pectoris and its therapeutic effects (通心络对不稳定性心绞痛血浆内皮素的影响及疗效观察). Chinese Medicine Modern Distance Education of China, 2008(05): 441-442.

[217] Guo WJ, Lai CL, Qin ZS, et al. Association of High Sensitivity C-reactive Protein,Lipoprotein (a) and Angina Pectoris and Effects of Tongxinluo Capsule (高敏 C 反应蛋白、脂蛋白(a)与心绞痛的关系及通心络干预对其的影响). Chinese Journal of Integrative Medicine on Cardio-/Cerebrovascular Disease, 2008(10): 1147-1149.

[218] Mo YQ, Wu SJ, Zhang C, et al. Effects of Tongxinluo capsule on heart rate variability in patients with angina pectoris in coronary heart disease (通心络胶囊对冠心病心绞痛患者心率变异性的影响). Chinese Journal of Difficult and Complicated Cases, 2007(03): 154-155.

[219] Huang YC, Tan XM, Liu E, et al. Clinical analysis and research on 88 cases of coronary heart disease treated with Tongxinluo capsule (通心络胶囊治疗冠心病 88 例临床分析研究). Modern Chinese Doctor, 2007(21): 78-97.

[220] Li CX, Bao J. Observation on the efficacy of Tongxinluo in the treatment of coronary heart disease (通心络治疗冠心病的疗效观察). Zhongyuan Medical Journal, 2007, 34(20): 91-92.

[221] Jin FB, Hou RT, Liu XY, et al. A study of the effect of Tongxinluo on endothelial function in coronary heart disease with type 2 diabetes mellitus (通心络对冠心病伴 2 型糖尿病的内皮功能影响研究). Shaanxi Journal of Traditional Chinese Medicine, 2007(02): 138-140.

[222] Luo XC, Liang XW, Chen JG. Therapeutic effect of Tongxinluo capsule in the treatment of angina pectoris with hyperlipidemia in coronary heart disease observed (通心络胶囊治疗冠心病心绞痛伴高脂血症疗效观察). Chinese Journal of Misdiagnostics, 2007(04): 741.

[223] Zhu XD, Liu WJ, Xu CR, et al. Effect of Tongxinluo Capsule on Serum CRP and Blood Lipids in Patients with Unstable Angina (通心络对不稳定型心绞痛患者血脂和血清 C-反应蛋白的影响). Heilongjiang Medical Journal, 2007(06): 414-415.

[224] Jin LJ, Zhou SC. Effect of Tongxinluo capsule on the concentration of high-sensitivity C-reactive protein in patients with angina pectoris (通心络胶囊对心绞痛患者高敏 C 反应蛋白浓度的影响). Hunan Journal of Traditional Chinese Medicine, 2007(02): 22-33.

[225] Zheng LW, Duan YC, Chen LZ. Therapeutic effect of Tongxinluo capsule in the treatment of unstable angina pectoris in 37 cases (通心络胶囊治疗不稳定性心绞痛 37 例疗效观察). Jilin Journal of Traditional Chinese Medicine, 2007(04): 18.

[226] Li JY, Lan ZH. Effect of Tongxinluo on the Serous Fibrinogen and C-reactive Protein in the Patients of Unstable Angina Pectoris (通心络对不稳定型心绞痛患者 FBG 和 CRP 的影响). 实用全科医学, 2007(04): 316-317.

[227] Ye DH, Xie YQ. Effect of Tongxinluo capsule on intercellular adhesion molecule-1 in patients with unstable angina pectoris (通心络对不稳定型心绞痛患者可溶性细胞间黏附分子-1 水平的影响). Chinese Journal of Difficult and Complicated Cases, 2007(01): 4-6.

[228] Tian FX, Wu KJ, Zhu HC. Tongxinluo capsule treatment of coronary heart disease unstable angina pectoris 60 cases efficacy observation (通心络胶囊治疗冠心病不稳定型心绞痛 60 例疗效观察). China Practical Medicine, 2007(36): 158-159.
[229] Liu JL, Liu YL. Clinical observation of Tongxinluo and amlodipine in the treatment of unstable angina pectoris (通心络、氨氯地平治疗不稳定心绞痛临床观察). China Practical Medicine, 2007(17): 70.
[230] Shi HY, Liu DJ. Tongxinluo Capsule for Unstable Angina with Hyperlipemia (通心络胶囊治疗不稳定型心绞痛伴高脂血症病人的临床研究). Chinese Journal of Integrative Medicine on Cardio-/Cerebrovascular Disease, 2007(01): 62-63.
[231] Yang M, Sun M, Liu JF, et al. Effect of Tongxinluo on TXB2/6-Keto-PGF1a in patients with angina pectoris of coronary artery disease (通心络对冠心病心绞痛患者 TXB2／6-Keto-PGF1a 的影响). Journal of Chinese Physician, 2007, 9(1): 134-135.
[232] Shi JF, Wang CY. The effect of Tongxinluo capsule on the blood rheology of patients with coronary heart disease angina pectoris and the therapeutic effect observation (通心络胶囊对冠心病心绞痛患者血液流变学的影响及疗效观察). Strait Pharmaceutical Journal, 2006(03): 120-121.
[233] Zou ST, Zhang HL. Clinical study on 126 cases of coronary heart disease treated by Tongxinluo (通心络治疗冠心病 126 例临床研究). Lishizhen Medicine and Materia Medica Research, 2006(06): 1044.
[234] Tang ZM, Cai CY. Observation on the efficacy of Tongxinluo capsule in the treatment of angina pectoris (通心络胶囊治疗心绞痛的疗效观察). Modern Journal of Integrated Traditional Chinese and Western Medicine, 2006(13): 1780-1781.
[235] Wang ZL, Lu JP, Zhang HJ. Clinical observation of Tongxinluo in the treatment of angina pectoris of coronary heart disease (通心络治疗冠心病心绞痛的临床观察). Journal of Medical Research, 2006(03): 81-82.
[236] Cao ZD, Mao JL, Bao ZY, et al. Clinical observation on the therapeutic effect of Tongxinluo capsule on depression in patients with coronary heart disease (通心络胶囊治疗冠心病患者抑郁症状的临床观察). Chinese Journal of Difficult and Complicated Cases, 2006(04): 251-253.
[237] Zhang QD, Wei ZD. Effect of tongxinluo capsule on blood lipid and plasma endothelin in patients with coronary artery disease (通心络胶囊对冠心病患者血脂及内皮素的影响). Chinese Journal of Cardiovascular Medicine, 2006(01): 20-23.
[238] Xu YM, Zhu Y. Clinical observation of Tongxinluo in the treatment of angina pectoris of coronary heart disease in the elderly (通心络治疗老年冠心病心绞痛的临床观察). China Medical Herald, 2006(27): 113-114.
[239] Zhang SR, Chen ZG, Lv FH, et al. Effect of Tongxinluo capsule on microcirculatory changes of nail bed and hemorrheology in patients with coronary artery disease and angina pectoris (通心络胶囊对冠心病心绞痛甲襞微循环和血液流变学变化的影响). Chinese Journal of Integrated Traditional and Western Medicine in Intensive and Critical Care, 2006(04): 237-239.
[240] Wang YH, Ying MZ. Clinical efficacy observation of Tongxinluo capsule in treating patients with coronary heart disease combined with premature beats (通心络胶囊治疗冠心病合并早搏患者临床疗效观察). Pharmaceutical Journal of Chinese People's Liberation Army, 2006(03): 239-242.
[241] Shen JJ, Wang M, Huang JY, et al. Observation on the efficacy of Tongxinluo capsule in the treatment of angina pectoris of coronary heart disease (通心络胶囊治疗冠心病心绞痛疗效观察). Zhejiang Journal of Integrated Traditional Chinese and Western Medicine, 2006(08): 487-488.
[242] Zhang M, Chen JP, Du YQ, et al. Effect of Tongxinluo capsule on QT interval dispersion in patients with coronary heart disease (通心络胶囊对冠心病患者 QT 间期离散度的影响). China Journal of Chinese Materia Medica, 2006(22): 1906-1907.
[243] Qi XY, Yang GL. Effect of Tongxinluo on serum C-reactive protein P-selectin in patients with unstable angina pectoris (通心络对不稳定心绞痛患者血清 C-反应蛋白 P-选择素的影响). Journal of Liaoning College

of Traditional Chinese Medicine, 2006(02): 81-82.

[244] Liu XJ, Wang LH. Clinical efficacy of Tongxinluo capsule in the treatment of unstable angina pectoris) (通心络胶囊治疗不稳定心绞痛临床疗效观察). Medical Journal of Chinese People's Health, 2006(15): 701-702.

[245] Huang ZG, Guo JH, Tang XX. Analysis of 43 cases of unstable angina pectoris treated with the combination of Tongxinluo and isosorbide mononitrate (通心络与单硝酸异山梨酯联合治疗不稳定型心绞痛 43 例分析). Modern Medicine Journal of China, 2006(02): 60-61.

[246] Chen PY, Chen WL, Li G, et al. Clinical reseach of Tongxinluo capsule on ET, MDA, CRP in treatment of angina pectoris (通心络胶囊对冠心病心绞痛内皮素、丙二醛、C-反应蛋白影响的临床研究). Hebei Journal of Traditional Chinese Medicine, 2005, 27(9): 695-697.

[247] Zhang QD, Zhang YQ, Wang XL. Effect of Tongxinluo Capsule on Blood Lipid in Patients with Coronary Disease (通心络胶囊对冠心病患者 Lp(a)等血脂的影响). Journal of Heze Medical College, 2005(04): 15-17.

[248] Zhang WL, Zhu WH. Tongxinluo capsule for the treatment of angina pectoris in coronary heart disease in 60 cases (通心络胶囊治疗冠心病心绞痛 60 例). Shaanxi Journal of Traditional Chinese Medicine, 2005, 26(11): 1142.

[249] Wei YY, Zhou MY. Tongxinluo capsule treatment of coronary heart disease angina pectoris 60 cases efficacy observation (通心络胶囊治疗冠心病心绞痛 60 例疗效观察). Journal of Chinese Practical Diagnosis and Therapy, 2005, 19(3): 206-207.

[250] Zhang XF, Xiao CJ, You SF, et al. Effect of Tongxinluo capsule on thyroid hormone levels in the treatment of heart failure caused by coronary heart disease (通心络胶囊对冠心病心力衰竭患者甲状腺激素水平影响的临床研究). Chinese Journal of Difficult and Complicated Cases, 2005, 4(3): 129-131.

[251] Huang YM, Huang HQ, Chen DM, et al. Observation on the efficacy of Tongxinluo capsule in the treatment of coronary heart disease (通心络胶囊治疗冠心病疗效观察). Chinese Journal of Clinical Medicine, 2005, 2(1): 20-21.

[252] Zhang YQ, Zhang X. Clinical observation on 60 cases of coronary heart disease angina with hyperlipidemia treated with Tongxinluo capsule (通心络胶囊治疗冠心病心绞痛伴高脂血症 60 例临床观察). Chinese Journal of Integrative Medicine on Cardio-/Cerebrovascular Disease, 2005, 3(11): 1008-1009.

[253] Yang GW, Li Y. Tongxinluo capsule for the treatment of angina pectoris of coronary heart disease in 32 cases (通心络胶囊治疗冠心病心绞痛 32 例). Chinese Journal of Integrative Medicine on Cardio-/Cerebrovascular Disease, 2005, 3(5): 455-456.

[254] Ran GX, Rong JY. 120 cases of chest paralysis treated with Tongxinluo capsule (通心络胶囊治疗胸痹 120 例). Henan Traditional Chinese Medicine, 2005(12): 44-45.

[255] Hong MY, Xia ZW. Tongxinluo capsule treatment of coronary heart disease angina pectoris 86 cases efficacy observation (通心络胶囊治疗冠心病心绞痛 86 例疗效观察). Zhejiang Journal of Integrated Traditional Chinese and Western Medicine, 2005(12): 757-758.

[256] Zhao YX, Liu YF, Yu HM, et al. Comparative observations on the intervention of Tongxinluo capsule and Bay Aspirin on inflammatory factors in patients with angina pectoris (通心络胶囊和拜阿司匹林对心绞痛患者炎性因子干预的对比观察). Chinese Journal of Integrated Traditional and Western Medicine, 2005(11): 1011.

[257] Li XQ, Kuang YD, Wang YG. Effects of Tongxingluo on Serum Soluble Cell Adhesion Molecules in Patients with Unstable Angina Pectoris (通心络胶囊对冠心病患者可溶性细胞间粘附分子 1 和血管细胞粘附分子 1 的影响). Chinese Journal of Arteriosclerosis, 2005(01): 99-100.

[258] Zhou HL, Wei ZD, Zhao H, et al. Effect of Tongxinluo Capsule in the Prethrombotic State on Patients with Unstable Angina Pectoris (通心络胶囊对不稳定型心绞痛病人血栓前状态的影响). Chinese Journal of Integrative Medicine on Cardio-/Cerebrovascular Disease, 2005(02): 102-103.

[259] Tian CX, Xu YJ. Effect of Tongxinluo capsule on C-reactive protein in patients with unstable angina pectoris (通心络胶囊对不稳定心绞痛患者 C 反应蛋白的影响). Journal of Bethune Military Medical College, 2005(02): 93-94.

[260] Yang G, Xuan CH, Cui L. Clinical observation of Tongxinluo capsule in the treatment of angina pectoris of coronary heart disease in the elderly (通心络胶囊治疗老年冠心病心绞痛的临床观察). Lishizhen Medicine and Materia Medica Research, 2005, 16(11): 1138-1139.

[261] Chen YL, Zhang J, Yang Y. Effect of Tongxinluo in treating patients with unstable angina pectoris (通心络治疗不稳定型心绞痛的临床观察). Journal of Logistics University of CAPF, 2005,14(3): 198-199.

[262] Luo HM, Fu DY, Ren MZ, et al. Clinical study on Tongxinluo Capsule affecting activity of platelet's GP Ⅱb/Ⅲa receptor in patients with coronary heart disease (通心络胶囊对冠心病人血小板GPⅡb/Ⅲa复合物活性影响的临床研究). Chinese Traditional Patent Medicine, 2005, 27(2): 181-183.

[263] Huang YM, Huang HQ, Chen DM, et al. Curative Effect Observation of Tongxiluo in Treatment of Coronary Heart Disease (通心络胶囊治疗冠心病(附 90 例疗效观察)). Heilongjiang Medical Journal, 2004, 28(9): 652-654.

[264] Lin HY, Li K, Li HY. Analysis of the efficacy of Tongxinluo capsule in the treatment of angina pectoris in coronary heart disease (通心络胶囊治疗冠心病心绞痛疗效分析). Journal of Guangxi University of Chinese Medicine, 2004(01): 19-20.

[265] Meng GX, Meng RQ, Zhou CB. Observations on the efficacy of Tongxinluo capsule in treating 60 cases of coronary heart disease (通心络胶囊佐治冠心病 60 例疗效观察). Shandong Medical Journal, 2004(19): 45-46.

[266] Zhang Q, Yang LH, Qiao P, et al. Clinical observation of the effects of Tongxinluo capsule injection on 68 patients with unstable angina pectoris (通心络治疗不稳定型心绞痛 68 例临床观察). Chinese Journal of Primary Medicine and Pharmacy, 2004(09): 60-61.

[267] Zhao XL, Han SB, Dong GX. Comparison of Tongxinluo and LuNanXinKang Alone or Combined in Treating Angina Pectoris (通心络和鲁南欣康单用或合用治疗心绞痛的比较). Journal of Practical Medical Techniques, 2004(15): 1971-1972.

[268] Zhao YX, Wen DF, Sun SW. Effects of “Tongxinluo Capsule” on PAG and Adhensive Molecule in Patients with Unstable Angina Pectoris (通心络胶囊对不稳定性心绞痛患者 PAG 及黏附分子水平的影响). Shanghai Journal of Traditional Chinese Medicine, 2004(10): 14-15.

[269] Sun SW, Zhao YX, Liang JL. Effect of Tongxinluo capsule on the function of platelet activation in patients with unstable angina pectoris (通心络胶囊对不稳定型心绞痛患者血小板活化功能的影响). Chinese Journal of Difficult and Complicated Cases, 2004(05): 260-262.

[270] Xiao YQ, Li WK. The Effect of Tongxinluo in Treating Patients with Unstable Angina Pectoris (通心络治疗不稳定型心绞痛的临床观察). Acta Medicinae Sinica, 2004,17(2): 152-153.

[271] Han L, Wang LW, Tang JR, et al. Tongxinluo treatment of unstable angina pectoris 57 cases efficacy observation (通心络治疗不稳定型心绞痛 57 例疗效观察). Shandong Medical Journal, 2004(25): 52.

[272] Sun YA, DingGF, Zhang LN, et al. Tongxinluo and isosorbide mononitrate combination for the treatment of angina pectoris of coronary heart disease in the elderly: efficacy observation (通心络与单硝酸异山梨酯合用治疗老年冠心病心绞痛疗效观察). Proceeding of Clinical Medicine, 2003(09): 673-674.

[273] Li J, Wang YZ. Tongxinluo capsule for the treatment of coronary heart disease angina pectoris in 52 cases (通心络胶囊治疗冠心病心绞痛 52 例). Shaanxi Journal of Traditional Chinese Medicine, 2003(02): 103-104.

[274] Zhang ZK, Zhang ZL. Tongxinluo in the treatment of angina pectoris, 36 cases (通心络治疗心绞痛 36 例). Practical Journal of Medicine & Pharmacy, 2003(10): 727.

[275] Meng QH, Guo H. Tongxinluo combined with elimination of cardiac pain in the treatment of angina pectoris of coronary heart disease efficacy observation (通心络联合消心痛治疗冠心病心绞痛疗效观察).

Chinese Journal of Urban and Rural Industrial Hygiene, 2003(01): 46-47.

[276] Guan YZ, Li RH. Effect of Tongxinluo on vascular endothelial function in patients with coronary heart disease combined with congestive heart failure (通心络对冠心病合并充血性心力衰竭患者血管内皮功能的影响). Chinese Remedies & Clinics, 2003(06): 510-511.

[277] Li ZJ, Xia RT, Wang DJ, et al. Observation on the efficacy of Tongxinluo on patients with angina pectoris of coronary heart disease (通心络对冠心病心绞痛病人的疗效观察). Chinese Journal of Celiopathy, 2003, 3(3): 210.

[278] Wang HJ, Huang YW, Sun J. Effect of tongxinluo capsule on function of vascular endothelium in patients with unstable angina pectoris. Zhongguo Zhong Xi Yi Jie He Za Zhi, 2003, 23(8): 587-589.

[279] Chen WS, Zhang CW. Tongxinluo capsule combined with isosorbide mononitrate tablets in the treatment of angina pectoris of coronary heart disease in 98 cases (通心络胶囊联合单硝酸异山梨酯片治疗冠心病心绞痛 98 例). Clinical Journal of Traditional Chinese Medicine, 2003, 15(5): 395.

[280] Liu J, Zhang LP. Clinical observation on the treatment of unstable angina pectoris with Tongxinluo capsule (通心络胶囊治疗不稳定型心绞痛临床观察). The Journal of Medical Theory and Practice, 2003(07): 785-786.

[281] Cai AH, Ma J, Zhang YJ, et al. Clinical observation of Tongxinluo capsule in the treatment of unstable angina pectoris of coronary heart disease (通心络胶囊治疗冠心病不稳定型心绞痛的临床观察). China Medical Journal Today, 2003, 3(4): 5-6.

[282] Feng P, Zeng H, Li P. Observation on the efficacy of Tongxinluo capsule in the treatment of angina pectoris of coronary heart disease (通心络胶囊治疗冠心病心绞痛疗效观察). Journal of Emergency in Traditional Chinese Medicine, 2003(06): 534.

[283] He JR, Gao XJ, Zhang L. Clinical observation on the treatment of coronary heart disease by Tongxinluo capsule (通心络胶囊治疗冠心病临床观察). Shanghai Medical & Pharmaceutical Journal, 2002(05): 203-204.

[284] Zhong BJ, Yang ML, Jia XX. Observation on the efficacy of Tongxinluo capsule in the treatment of angina pectoris of coronary heart disease (通心络胶囊治疗冠心病心绞痛疗效观察). Journal of Zhangjiakou Medical College, 2002(03): 11-12.

[285] Song B, Xu GX. Therapeutic efficacy of Tongxinluo in the treatment of unstable angina pectoris observed (通心络治疗不稳定性心绞痛疗效观察). Journal of Modern Medicine & Health, 2002(12): 1077.

[286] Xiao WL, Dai H, Jiang ZA, et al. Study on the protective effect of Tongxinluo capsule on vascular endothelial cells in patients with unstable angina pectoris (通心络胶囊对不稳定性心绞痛患者血管内皮细胞保护作用的研究). Chinese Journal of Cardiology, 2002(05): 15.

[287] Li GZ, Zhou YH, Wu LX. Effect of Tongxinluo capsule on thrombin activity and fibrinogen content in patients with unstable angina pectoris (通心络胶囊对不稳定性心绞痛患者凝血酶活性及纤维蛋白原含量的影响). Handan Medical College Journal, 2002(02): 137.

[288] Wang JC, Sun JY, Li HB, et al. Comparative Study of Tongxinluo Capsules and Sordi in Treatment of Patients with Silent Myocardial Ischemia (通心络胶囊和硝酸异山梨醇治疗无症状心肌缺血的对照研究). Chinese Journal of Basic Medicine in Traditional Chinese Medicine, 2001(01): 49-51.

[289] Xu GC, Liu ZL, Yin YJ. CLINICAL OBSERVATION OF THE DOSE AND TIME EFFECT OF TONGXINLUO CAPSULE IN TREATMENT OF ANGINA PECTORIS CAUSED BY CORONARY HEART DISEASE (通心络胶囊治疗冠心病心绞痛量效时效的临床观察). Chinese Journal of New Drugs, 2000, 9(3): 200-202.

[290] Zhang XQ, Pan ZH, Xu YL, et al. Clinical observation of Tongxinluo capsule in the treatment of angina pectoris of coronary heart disease in the elderly (通心络胶囊治疗老年冠心病心绞痛的临床观察). Geriatrics & Health CARE, 2000(02): 102-103.

[291] Lv SC, Jiang J, Yuan LF. Tongxinluo treatment of coronary heart disease angina pectoris 49 cases efficacy observation (通心络治疗冠心病心绞痛 49 例疗效观察). Clinical Focus, 2000(23): 1077-1078.

[292] Ma CS, Wu XP. Therapeutic efficacy of Tongxinluo capsule in the treatment of progressive angina pectoris observed (通心络胶囊治疗进行性心绞痛疗效观察). Tianjin Journal of Traditional Chinese Medicine, 2000(03): 10.

[293] Fu XH, Zhang ZM, Li YY, et al. Effect of Tongxinluo capsule on QT interval dispersion in patients with coronary heart disease (通心络胶囊对冠心病患者 QT 间期离散度的影响). Heilongjiang Medical Journal, 1999(05): 45.

[294] Wang Y, Li Z. Observation on the efficacy of Tongxinluo glue in the treatment of coronary heart disease with hyperlipidemia (通心络胶襄治疗冠心病并高脂血症的疗效观察). Modern Journal of Integrated Traditional Chinese and Western Medicine, 1999(09): 1427-1428.

[295] Li XD, Yao HB. Clinical observation on the treatment of coronary heart disease angina pectoris with blood stasis by Tongxinluo capsule (通心络胶囊治疗冠心病心绞痛血瘀证的临床观察). Chinese Journal of Basic Medicine in Traditional Chinese Medicine, 1999, 5(8): 41-43.

[296] Gao YC, Guo JL, Wang S. Observations on 92 cases of coronary heart disease angina pectoris treated with Tongxinluo capsule (通心络胶囊治疗冠心病心绞痛 92 例观察). Chinese Medicine Correspondence Newsletter, 1998(01): 26-27.

2 Non-RCTs (n = 3)

[1] Han YH, Li XF. Clinical observation of Tongxinluo in the treatment of coronary heart disease combined with cerebral infarction (通心络治疗冠心病合并脑梗死的临床观察). China Practical Medicine, 2008(10): 137-138.

[2] Guo DL, Wang KQ. Clinical observation on the treatment of angina pectoris in coronary heart disease by Tongxinluo capsule (通心络胶囊治疗冠心病心绞痛临床观察). Journal of Baotou Medical College, 2008(01): 58-59.

[3] Zhang Y, Zhou H, Wang E. A clinical investigation on tong xin luo capsule in treatment of coronary heart disease with silent myocardial ischemia. J Tradit Chin Med, 2000, 20(2): 93-95.

3 Ineligible interventions (n = 26)

[1] Sun YH, Hu JJ, Liu YL. Exploring the efficacy of using Tongxinluo in the treatment of stable angina pectoris and its effect on endothelial function in patients (使用通心络治疗稳定型心绞痛的疗效及对患者内皮功能的影响探析). Contemporary Medicine Forum, 2015, 13(15): 182-183.

[2] Fu DH, Song XL, Feng CH. Observation on the efficacy of Tongxinluo capsule on angina pectoris after coronary intervention (通心络胶囊对冠心病介入术后心绞痛的疗效观察). Journal of Guiyang College of Traditional Chinese Medicine, 2012, 34(03): 171-172.

[3] Kang SP, Huang JL, Zhou YX. Effect of Tongxinluo capsule on the quality of life of elderly patients with stable angina pectoris (通心络胶囊对老年稳定性心绞痛患者生活质量影响). Fujian Journal of Traditional Chinese Medicine, 2012, 43(05): 10-11.

[4] Meng T, Bao YJ. Tongxinluo Qumei Trimetazidine Treatment of Asymptomatic Myocardial Ischemia Randomized Controlled Clinical Study (通心络与曲美他嗪治疗无症状心肌缺血随机对照临床研究). Journal of Practical Traditional Chinese Internal Medicine, 2012, 26(13): 17-18.

[5] Du LJ, Du LK, Sun P. Study on the effect of Tongxinluo capsule on clinical efficacy and MMP-9 content in patients with angina pectoris (通心络胶囊对心绞痛患者临床疗效及 MMP-9 含量影响的研究). Chinese Journal of Information on Traditional Chinese Medicine, 2012, 29(04): 107-108.

[6] Gong JP, Fang HT. Effect of Tongxinluo capsule on serum C-reactive protein and blood lipids in patients with asymptomatic myocardial ischemia of coronary heart disease (通心络胶囊对冠心病无症状型心肌缺血患者血清 C 反应蛋白和血脂的影响). Journal of Emergency in Traditional Chinese Medicine, 2011, 20(12): 2007-2008.

[7] Zhang HM, Du TM. Clinical observation of Tongxinluo combined with trimetazidine in the treatment of unstable angina pectoris (通心络联合曲美他嗪治疗不稳定型心绞痛临床观察). Chinese Community

Physician, 2011, 13(15): 24.
[8] Xu HM, Sun YZ. Tongxinluo capsule treatment of angina pectoris 66 cases efficacy observation (通心络胶囊治疗心绞痛 66 例疗效观察). Zhejiang Journal of Traditional Chinese Medicine, 2010, 45(07): 545.
[9] Li XH, Ni Q. 128 cases of stable angina pectoris in coronary heart disease treated with Tongxinluo capsule (通心络胶囊治疗冠心病稳定型心绞痛 128 例). Guangming Journal of Chinese Medicine, 2010, 25(10): 1819-1821.
[10] Wang JF, Wang QR. Tongxinluo capsule for the treatment of coronary heart disease stable angina pectoris in 45 cases (通心络胶囊治疗冠心病稳定性心绞痛 45 例). Journal of Shaanxi University of Chinese Medicine, 2009, 32(05): 16-39.
[11] Sun LP, Zhang LC. Clinical observation on the treatment of stable angina pectoris with trimetazidine and Tongxinluo capsule (曲美他嗪和通心络胶囊治疗稳定型心绞痛临床观察). Journal of Inner Mongolia Medical University, 2008(03): 205-206.
[12] Kang JH, Guo LN, Li LM, et al. Effect of TongXinLuo capsule in treating stable angina pectoris (通心络胶囊治疗冠心病心绞痛临床疗效观察). Chinese Journal of Practical Internal Medicine, 2008, 28(S2): 59-60.
[13] Wang DW, Zheng JF, Ma BW. Tongxinluo capsule treatment of coronary heart disease angina pectoris 66 cases observation (通心络胶囊治疗冠心病心绞痛 66 例观察). Zhejiang Journal of Traditional Chinese Medicine, 2007(07): 431.
[14] Guo L, Ning HH, Yan HX, et al. Ridit analysis of Tongxinluo capsule in the treatment of coronary angina in elderly people (通心络胶囊治疗老年人冠心病心绞痛的 Ridit 分析). Chinese Journal of Health Statistics, 2006(04): 338.
[15] Hou Y, Zhang L. Observation on the efficacy of Tongxinluo capsule in the treatment of occult coronary heart disease (通心络胶囊治疗隐匿型冠心病疗效观察). Modern Journal of Integrated Traditional Chinese and Western Medicine, 2006(08): 1025-1026.
[16] Liu X, Liao YH, Zhu YG, et al. Clinical Study on Stable Angina Pectoris by Tongxinluo Capsules and Xinaole (通心络胶囊合用欣奥乐注射液治疗稳定型心绞痛的临床研究). Chronic Pathematology Journal, 2006(11): 11-12.
[17] Li R, Zhang X. Clinical observation of Tongxinluo capsule in the treatment of stable angina pectoris (通心络胶囊治疗稳定型心绞痛临床观察). Jilin Journal of Traditional Chinese Medicine, 2005(01): 20.
[18] Zhang H, Sun B, Zhang B. Observation on the efficacy of Tongxinluo capsule in the treatment of angina pectoris in 43 cases (通心络胶囊治疗心绞痛 43 例疗效观察). Journal of Bethune Military Medical College, 2005(04): 220.
[19] Li JM, Li ZZ, Chu QF. Tongxinluo capsule for the treatment of angina pectoris in coronary heart disease (通心络胶囊治疗冠心病心绞痛). Journal of Medical Forum, 2005(24): 69-70.
[20] Zhang H, Li YP. Evaluation of the efficacy of Tongxinluo in the treatment of angina pectoris in coronary heart disease (通心络治疗冠心病心绞痛的疗效评价). Journal of Modern Medicine & Health, 2004, 20(15): 1480.
[21] Luan J, Li WF, Huang HZ. Tongxinluo capsule for the treatment of angina pectoris in coronary heart disease in 30 cases (通心络胶囊治疗冠心病心绞痛 30 例). Chinese Journal of Integrated Traditional and Western Medicine, 2004(03): 277.
[22] Fu J, Liu XD. Observation on the efficacy of Tongxinluo capsule in the treatment of angina pectoris of coronary heart disease (通心络胶囊治疗冠心病心绞痛疗效观察). Journal of Jiangxi Medical College, 2002(06): 55.
[23] Xu GC, Gao RL, Liu JL, et al. DOUBLE BLIND CONTROL OBSERVATION OF TONGXINLUO CAPSULE ON TREATMENT OF PATIENTS WITH ANGINA PECTORIS CAUSED BY CORONARY HEART DISEASE (通心络胶囊治疗冠心病心绞痛双盲对照研究). Chinese Journal of New Drugs, 2000, 9(2): 109-111.
[24] Zhang B, Wang H, Niu JH, et al. Clinical observation of Tongxinluo in the treatment of asymptomatic

myocardial ischemia in coronary heart disease (通心络治疗冠心病无症状心肌缺血的临床观察). Chinese Journal of Basic Medicine in Traditional Chinese Medicine, 2000, 6(12): 33-34.

[25] Wu Q, Wu XC, Wu G. Tongxinluo capsule treatment of coronary heart disease angina pectoris 30 cases efficacy observation (通心络胶囊治疗冠心病心绞痛 30 例疗效观察). Modern Journal of Integrated Traditional Chinese and Western Medicine, 1998(11): 1770-1771.

[26] Fu J, Liu HC, Cao LB. Clinical efficacy observation of Tongxinluo capsule in the treatment of angina pectoris of coronary heart disease (通心络胶囊治疗冠心病心绞痛临床疗效观察). Acta Chinese Medicine and Pharmacology, 1998(04): 11.

4 Ineligible patients (including patients with recent revascularization) (n = 59)

[1] Meng XX, Wang J, Duan PH, et al. Effects of Tongxinluo capsules combined with Nicorandil on angina pectoris after PCI in patients with single-vessel coronary heart disease (通心络胶囊联合尼可地尔片治疗单支血管病变冠心病患者 PCI 术后心绞痛的效果). Medical Journal of Chinese People's Health, 2023, 35(10): 109-112.

[2] Meng XX, Wang J, Wang ZQ, et al. Observation on the effect of Tongxinluo capsule combined with nicorandil on vascular endothelial cell function and myocardial enzyme profile in patients after PCI for acute myocardial infarction (通心络胶囊联合尼可地尔对急性心肌梗死 PCI 术后患者血管内皮细胞功能及心肌酶谱的影响观察). Harbin Medical Journal, 2023, 43(01): 122-123.

[3] Liang HY, Feng XZ. Effect of Tongxinluo capsule on plasma endothelin-1, vascular endothelial growth factor and micro RNA-155 levels after percutaneous coronary intervention in patients with acute coronary syndrome (通心络胶囊对急性冠状动脉综合征患者经皮冠状动脉介入术后血浆内皮素-1、血管内皮生长因子及微小 RNA-155 含量的影响). Prevention and Treatment of Cardio-Cerebral-Vascular Disease, 2023, 23(4): 57-59.

[4] Sun J, Liu X. Effects of Tongxinluo capsule combined with rosuvastatin calcium on ET-1,NO, cTnI and vWF levels in patients with coronary heart disease and angina pectoris (通心络胶囊联合瑞舒伐他汀钙对冠心病心绞痛患者 ET-1、NO、cTnI、vWF 水平的影响). Clinical Research and Practice, 2022, 7(03): 134-137.

[5] Zhang YY, Yao HY. Effect of Tongxinluo capsule on cardiovascular clinical events in patients with coronary heart disease (通心络胶囊治疗冠心病的临床疗效及其安全性). Journal of clinical rational drug use, 2021, 14(09): 7-9.

[6] Pan QQ, Lu JZ, Zhang B, et al. Analysis of the Curative Effects of Tongxinluo Capsules on Patients After Coronary Interventional Therapy (通心络胶囊对冠心病介入治疗手术后患者的疗效). World Chinese Medicine, 2021, 16(10): 1572-1575.

[7] Wang WW, Yuan GQ, Hong H, et al. Effects of Tongxinluo Capsule Combined with Clopidogrel Bisulfate after PCI in Patients with Acute Myocardial Infarction (通心络胶囊联合硫酸氢氯吡格雷对急性心肌梗死经皮冠状动脉介入治疗(PCI)术后患者的临床疗效). World Chinese Medicine, 2021, 16(11): 1649-1653.

[8] Hong CZ, Zheng WB, Chen WJ, et al. Clinical effect of Tongxinluo capsule adjuvant to aspirin and isosorbide mononitrate in the treatment of acute myocardial infarction patients with PCI after surgery (通心络胶囊辅助阿司匹林、单硝酸异山梨酯治疗急性心肌梗死患者 PCI 术后的临床效果). Journal of clinical rational drug use, 2021, 14(28): 37-39.

[9] Chen Z, Feng J, Jia ZS, et al. Clinical Study of Tongxinluo Combined with Ticagrelor in the Treatment of Acute Coronary Syndrome Undergoing PCI (通心络联合替格瑞洛治疗急性冠脉综合征经皮冠状动脉介入治疗患者的临床研究). World Chinese Medicine, 2020, 15(20): 3084-3087.

[10] Guan LS, Yin WT. Clinical therapeutic observation of Tongxinluo in the prevention of restenosis after stenting in coronary heart disease (预防冠心病支架术后再狭窄中通心络的临床治疗观察). China Health Care Nutrition, 2019, 29(15): 96.

[11] Wang TH, Cen YG, Tian XL, et al. Effects of Tongxinluo capsule combined with atorvastatin on serum vWF, hs-CRP and VEGF levels in patients with acute coronary syndrome after PCI (通心络胶囊联合阿托伐他汀对

急性冠脉综合征行 PCI 术后血清 vWF、hs-CRP、VEGF 水平影响). Chinese Journal of Clinical Research, 2019, 32(8): 1095-1098.

[12] Zhang YL, Bai J. To investigate the effect of tongxinluo combined with tigrillo in the treatment of patients with acute coronary syndrome who received PCI (为接受 PCI 的急性冠状动脉综合征患者用通心络联合替格瑞洛进行治疗的效果探究). Contemporary Medicine Forum, 2019, 17(20): 125-128.

[13] Sun ZR, Lang ZL. Effect of Tongxinluo capsule on NF-κB, IL-6 and TNF-α expression after coronary stenting in patients with coronary artery heart disease (通心络胶囊对冠心病冠状动脉支架植入术后患者 NF-κB、IL-6、TNF-α 表达的影响及治疗效果分析). Chinese Journal of the Frontiers of Medical Science (Electronic Version), 2018, 10(8): 43-45.

[14] Xiang YM, Zhang T, Wang L, et al. "Clinical efficacy of Tongxinluo Capsules in the treatment of angina pectoris (“通心络胶囊”治疗心绞痛的临床疗效的观察). Journal of Frontiers of Medicine, 2017, 7(25): 328-329.

[15] Zhang L, Xu TF. Clinical efficacy observation of Tongxinluo capsule in the treatment of angina pectoris of coronary heart disease (通心络胶囊治疗冠心病心绞痛的临床疗效观察). Cardiovascular Disease Journal Of integrated traditional Chinese and Western Medicine, 2017, 5(02): 79-80.

[16] Zhu XS, Yang F, Ma KZ, et al. Effect of Tongxinluo on cardiovascular clinical events in patients with coronary artery disease diagnosed by coronary angiography (通心络对冠状动脉造影确诊的冠心病患者心血管临床事件的影响). Chongqing Medicine, 2017, 46(A03): 34-35.

[17] Chen XW, Huang ZW, Tian LH, et al. Therapeutic effect and influence of Tongxinluo capsules on inflammatory responses and vascular endothelial function in patients with coronary heart disease suffered from angina pectoris after percutaneous coronary intervention (通心络胶囊对冠心病 PCI 术后心绞痛患者的疗效及炎性反应和血管内皮功能的影响). Chinese Journal of New Drugs, 2017, 26(20): 2459-2462.

[18] Chen GY, Qian YF, Fang CM. Effects of Tongxinluo Capsules on Serum Cy PA and MMP-9 Levels in Patients with Coronary Heart Disease after PCI (通心络胶囊对冠心病患者 PCI 术后血清亲环素 A 和基质金属蛋白酶 9 的影响). China Pharmacy, 2017, 28(24): 3365-3367.

[19] Peng ZP, Yu Y, Zhu WW. Effect of Tongxinluo capsule on serum EMPs and MMP-9 after PCI in patients with acute myocardial infarction (通心络胶囊对急性心肌梗死患者 PCI 术后血清 EMPs 及 MMP-9 的影响). Modern Journal of Integrated Traditional Chinese and Western Medicine, 2017, 26(28): 3117-3119.

[20] Duan BJ, Lin DZ, Xiong FQ, et al. Efficacy and mechanism of Tongxinluo capsule in treating angina pectoris of coronary heart disease with Qi deficiency and blood stasis (通心络胶囊治疗气虚血瘀型冠心病心绞痛的疗效及作用机制). Chinese Journal of Difficult and Complicated Cases, 2016, 15(10): 1065-1068.

[21] Hao JH, Ning XH. Observation of Tongxinluo capsule on angina in aged patients with coronary heart disease after percutaneous coronary intervention (通心络胶囊治疗老年冠心病经皮冠状动脉介入治疗术后心绞痛临床观察). Hebei Journal of Traditional Chinese Medicine, 2015, 37(06): 897-900.

[22] Li Y, Han L, Cui LF, et al. Clinical study on Tongxinluo Capsules combined with atorvastatin in treatment of acute myocardial infarction with PCI postoperative inflammatory reaction (通心络胶囊联合阿托伐他汀治疗急性心肌梗死 PCI 术后炎性反应的临床研究). Drugs & Clinic, 2015, 30(01): 36-39.

[23] Geng LM, Li KF. Tongxinluo Intervention of Patients with Acute Coronary Syndrome after PIC Surgery (通心络对急性冠脉综合征患者 PCI 术后的干预作用). Clinical Journal of Chinese Medicine, 2015, 7(07): 20-22.

[24] Tian ZT, Li HL, Li K. Tongxinluo Capsule on Acute Myocardial Infarction after Percutaneous Coronary Artery Interventional Therapy after Operation in 30 Cases (通心络胶囊干预急性心肌梗死经皮冠状动脉介入治疗术后 30 例). Chinese Journal of Experimental Traditional Medical Formulae, 2014, 20(02): 196-200.

[25] Meng X, Liu DK, Zhang SY. Clinical Observation of Tongxinluo Capsule in Treatment of 186 Patients with Angina Caused by Coronary Heart Disease (通心络胶囊治疗冠心病心绞痛 186 例临床观察). JOURNAL OF MEDICAL INFORMATION, 2013(18): 282.

[26] Li YS, Li HP, Yuan Y, et al. Clinical Observation of Tongxinluo Capsule in Treating Coronary Heart Disease

and Angina Pectoris Patient after PCI (通心络胶囊对冠心病心绞痛患者介入治疗后的影响). World Chinese Medicine, 2013, 8(10): 1251-1252.

[27] Tao WG, Zhu KY. Curative effect of Tongxinluo in treatment of angina after percutaneous coronary intervention(PCI) operation (通心络治疗 PCI 术后心绞痛的疗效观察). Chinese Medical Innovations, 2012, 9(05): 9-10.

[28] Dong SQ, Dong YW. Effect of Tongxinluo capsule on inflammatory factors and vascular endothelial function after percutaneous coronary intervention in patients with acute myocardial infarction (通心络胶囊对急性心肌梗死患者经皮冠状动脉介入治疗术后炎症因子及血管内皮功能的影响). Chinese Journal of Clinicians (Electronic Edition), 2012, 6(07): 1865-1867.

[29] Bao LX, Xu YL. Effects of "Tongxinluo Capsule" and conventional western medicine on serum hypersensitive C-reactive protein in coronary heart disease (通心络胶囊结合西医常规疗法对冠心病患者血清超敏 C 反应蛋白的影响). Shanghai Journal of Traditional Chinese Medicine, 2011, 45(03): 32-33.

[30] Dai GW, Yang SJ. Clinical observation of Tongxinluo capsule intervention in restenosis after PCI for coronary heart disease (通心络胶囊干预冠心病 PCI 术后再狭窄临床观察). Guangming Journal of Chinese Medicine, 2011, 26(09): 1823-1824.

[31] Han XT, Cui XJ, Li YR, et al. Effect of Tongxinluo Capsules on Lipids, Plasma High-Sensitivity C-Reactive Protein, and Matrix Metalloproteinase-9 after PCI in Patients with Unstable Angina Pectoris (通心络胶囊对不稳定型心绞痛患者 PCI 术后血脂、血浆高敏 C 反应蛋白、基质金属蛋白酶-9 的影响). Journal of Emergency in Traditional Chinese Medicine, 2011, 20(06): 873-874.

[32] Chen ZQ, Hong L, Wang H, et al. Effect of Tongxinluo Capsule on Platelet Activities and Vascular Endothelial Functions as well as Prognosis in Patients with Acute Coronary Syndrome Undergoing Percutaneous Coronary Intervention (通心络胶囊对急性冠状动脉综合征患者介入治疗后血小板活化和血管内皮功能及预后的影响). Chinese Journal of Integrated Traditional and Western Medicine, 2011, 31(04): 487-491.

[33] Yin CH, Bi DP, Du M. Effect of tongxinluo capsule on platelet aggregation function in patients with aspirin resistance. Zhongguo Zhong Xi Yi Jie He Za Zhi, 2010, 30(4): 380-382.

[34] Zhao H, Tian JH, Hao XY, et al. EFFECTS OF TONGXINLUO ON INFLAMMATORY FACTORS IN PATIENTS WITH CORONARY HEART DISEASE AFTER PERCUTANEOUS CORONARY INTERVENTION (通心络对冠心病病人 PCI 术后炎症因子的影响). Journal of Qingdao University Medical College, 2010, 46(04): 349-351.

[35] Liu YJ, Yang ZE. Effect of danshen tongxinluo combination therapy on ultrasensitive C-reactive protein in elderly patients with coronary heart disease (丹参通心络联用治疗对老年冠心病患者超敏 C 反应蛋白的影响). Chinese Community Physician, 2010, 12(16): 156.

[36] He ZW, Peng MY, Lu BF. Clinical observation of pravastatin combined with Tongxinluo capsule to improve pulse pressure in patients with coronary heart disease combined with hypertension (普伐他汀联合通心络胶囊改善冠心病合并高血压患者脉压临床观察). Chinese Journal of Information on Traditional Chinese Medicine, 2010, 17(02): 72-73.

[37] Liao CL, Liang D, Pan GZ, et al. Effect of Tongxinluo capsule on CRP and ventricular remodeling after acute myocardial infarction (通心络胶囊对急性心肌梗死后 CRP 及心室重构的影响). Clinical Journal of Traditional Chinese Medicine, 2010, 22(04): 304-305.

[38] Liang YM, Wang ZJ, Su XY. The effect of Tongxinluo capsules on coronary restennosis of patients with acute myocardial infarction after percutaneoas coronary intervention (通心络胶囊对心肌梗死冠状动脉介入治疗患者术后再狭窄的疗效评价). Chinese Journal of Integrated Traditional and Western Medicine in Intensive and Critical Care, 2010, 17(3): 175-176.

[39] Zhang H T, Jia Z H, Zhang J, et al. No-reflow protection and long-term efficacy for acute myocardial infarction with Tongxinluo: a randomized double-blind placebo-controlled multicenter clinical trial (ENLEAT Trial)). Chin Med J (Engl), 2010, 123(20): 2858-2864.

[40] Wu ZY, Yang DC, Hou B, et al. Effects of Tongxinluo Capsules on interventional therapy of patients with unstable angina (通心络胶囊在不稳定型心绞痛患者介入治疗中的作用). China Medical Herald, 2010, 7(20): 17-20.
[41] Zheng H, Teng ZH, Ma LQ. The effect of Tongxinluo capsule on level of serum CRP and blood lipid in patients with ACS post PCI (通心络胶囊对急性冠状动脉综合征介入术后患者 C-反应蛋白、血脂的影响). Chinese Journal of Difficult and Complicated Cases, 2010, 9(07): 487-488.
[42] Wu JH, Li SJ. Clinical observation on non-ST-segment elevation myocardial infarction treated with the aid of Tongxinluo capsule (通心络胶囊辅助治疗非 ST 段抬高心肌梗死临床观察). Chinese Community Physician, 2009, 11(11): 111-112.
[43] Yu Q, Li YJ. The Clinical Study of Treating 32 cases Coronary Heart Disease with Tongxinluo Capsule (通心络治疗冠心病 32 例临床研究). Hebei Medicine, 2008(06): 692-693.
[44] Qian B, Zhao L. Clinical efficacy observation of Tongxinluo capsule in the treatment of angina pectoris of coronary heart disease (通心络胶囊治疗冠心病心绞痛临床疗效观察). Chinese Primary Health Care, 2008(08): 101.
[45] Xiao HB, Zhang DD, Gu J. Near- and long-term efficacy of Tongxinluo on coronary heart disease after interventional therapy (通心络对冠心病介入治疗后近远期疗效观察). Chinese Journal of Postgraduates of Medicine, 2007,30(5): 47-48.
[46] Xiao HB, Zhang DD, Gu J. Effects of tongxinluo on C-reactive protein and clinical prognosis in patients after coronary stenting (通心络对冠心病支架术后 C 反应蛋白及预后的影响). Journal of Interventional Radiology, 2007(08): 520-522.
[47] Chen WM, Yu CZ. CLINICAL STUDY OF CORONARY HEART DISEASE TREATED WITH TONGXINLUO CAPSULE (通心络胶囊治疗冠心病心绞痛临床疗效观察). Modern Hospitals, 2006(06): 57-58.
[48] Yao FM, Liu N, Ge GY. Clinical study of Tongxinluo capsule intervention in restenosis after PCI in patients with coronary artery disease (通心络胶囊干预冠心病患者 PCI 术后再狭窄的临床研究). Chinese Journal of Difficult and Complicated Cases, 2006(03): 191-192.
[49] Lu H, Hu HM, Zhao HF. Clinical observation on 100 cases of angina pectoris treated with Tongxinluo capsule plus elimination of cardiac pain (通心络胶囊加消心痛治疗心绞痛 100 例临床观察). Xinjiang Journal of Traditional Chinese Medicine, 2005(06): 13-14.
[50] Wu YF, Li QH. Clinical observation on 123 cases of angina pectoris in coronary heart disease treated with Tongxinluo capsule (通心络胶囊治疗冠心病心绞痛 123 例临床观察). Handan Medical College Journal, 2005(02): 114-115.
[51] Qi YX, Yan SL. Clinical observation on 82 cases of angina pectoris treated with Tongxinluo capsule (通心络胶囊治疗心绞痛 82 例临床观察). The Journal of Medical Theory and Practice, 2005(07): 790-791.
[52] Wei J, Zhang HC, Guo XM. Clinical efficacy analysis of Tongxinluo in the treatment of angina pectoris of coronary heart disease (通心络治疗冠心病心绞痛的临床疗效分析). Chinese Journal of Misdiagnostics, 2005(12): 2264-2265.
[53] Li SR, Qi XY, Zhao YJ, et al. Effect of Tongxinluo on endothelium-dependent vasodilatation in coronary artery disease (通心络和阿伐他汀对冠心病患者内皮功能的对比研究). Clinical Focus, 2004, 19(15): 845-847.
[54] Huang YQ, Li FC, Fu B. Clinical study on 37 cases of coronary heart disease with hyperlipidemia treated by Tongxinluo in the elderly (通心络治疗老年冠心病并高脂血症 37 例临床研究). Chinese Journal of Traditional Medical Science and Technology, 2004, 11(4): 215.
[55] Fan Y, Zhang YN, Jiang LH, et al. Effects of Tongxinluo capsule on patients with post-infarction angina pectoris (通心络对陈旧性心肌梗塞再发心绞痛疗效分析). Journal of Harbin Medical University, 2003, 37(5): 439-440.
[56] Ma LP, Wang FL, Wang ML. Effect of Tongxinluo on cardiac function and exercise tolerance in patients

with coronary heart disease and heart failure (通心络对冠心病心力衰竭患者心功能和运动耐量的影响). China Clinical Rehabilitation, 2003, 7(21): 2975.

[57] Feng P, Zeng H, Li P. Observation on the efficacy of Tongxinluo capsule in the treatment of angina pectoris of coronary heart disease (通心络胶囊治疗冠心病心绞痛疗效观察). Journal of Emergency in Traditional Chinese Medicine, 2003, 12(6): 534.

[58] Yang HS, Li C. Clinical analysis of 60 cases of coronary heart disease treated with Tongxinluo plus Ceville (通心络加西维尔治疗冠心病 60 例临床分析). Fujian Medical Journal, 2003(05): 106.

[59] Jia Z, Gu FS, Xue YF. Effect of Tongxinluo Capsule in Treating Variant Angina Pectoris Patients and Its Influence on Endothelial Function (通心络胶囊治疗冠心病变异性心绞痛临床疗效及对内皮功能的影响). Chinese Journal of Integrated Traditional and Western Medicine, 1999, 19(11): 651-652.

5 Duplicated reports from the same trial (n = 1)

Qian XX, Chen YM, Liu Y, et al. Effect of tongxinluo capsule on endothelial function in stable angina pectoris patients (通心络治疗稳定型心绞痛的临床疗效及对内皮功能的影响). Chinese Journal of Pathophysiology, 2006(09): 1698-1701.

6 Studies with misdescribed design elements of randomized controlled trials (n = 12)

[1] Bao XY, Wang H, Li SS, et al. Effect of Tongxinluo capsule combined with clopidogrel in the treatment of coronary heart disease (通心络胶囊联合氯吡格雷治疗冠心病的效果). Henan Medical Research, 2024,33(07):1297-1301.

[2] Wang ML, Yang XH, Zhang HL. Randomized,Double-Blind,Placebo Controlled,Multi-Center Clinical Trial of Tongxinluo Capsules for Angina Pectoris (通心络胶囊对冠心病心绞痛随机、双盲、安慰剂平行对照、多中心临床试验). World Chinese Medicine, 2019, 14(11): 2973-2977.

[3] Li QQ, Qiu HM. Analyzing the effects of Tongxinluo capsule on the efficacy and endothelial function of coronary heart disease variant angina pectoris (分析通心络胶囊对冠心病变异性心绞痛疗效及内皮功能的影响). Medical Diet and Health, 2019, 0(1): 197-198.

[4] Yu DH, Liu XD. Efficacy of Tongxinluo capsule in the treatment of stable angina pectoris in coronary heart disease (通心络胶囊治疗冠心病稳定型心绞痛的疗效). Journal of Dietary Health, 2018, 5(19): 70.

[5] Yang Y, Ye JH. Evaluation of the effect of Tongxinluo capsule combined with conventional treatment of coronary angina with hyperhomocysteinemia (通心络胶囊联合常规治疗冠心病心绞痛伴高同型半胱氨酸血症的效果评价). Journal of North Pharmacy, 2017, 14(10): 133.

[6] Zhao LX, Wang HL. Observation on the efficacy of Tongxinluo capsule with western medicine in the treatment of stable angina pectoris (通心络胶囊配合西药治疗稳定型心绞痛疗效观察). China's Naturopathy, 2014, 22(02): 50.

[7] Huang L, Quan Y. Effect of Tongxinluo on vascular endothelial function in patients with stable angina pectoris of coronary heart disease (通心络对冠心病稳定型心绞痛患者血管内皮功能的影响). Medical Journal of Chinese People's Health, 2009, 21(9): 985.

[8] Lin H, Liu Y. Effect of Tongxinluo capsule on asymptomatic myocardial ischemia after myocardial infarction (通心络胶囊对心肌梗死后无症状心肌缺血的影响). Contemporary Medicine, 2008, 14(20): 168-169.

[9] Ding HL, Wang XL. Tongxinluo capsule for the treatment of coronary heart disease stable angina pectoris in 30 cases (通心络胶囊治疗冠心病稳定型心绞痛 30 例). Journal of Practical Traditional Chinese Internal Medicine, 2004(01): 67.

[10] Xie QF, Ye AL, Zhao HS. Clinical observation on the treatment of angina pectoris of coronary heart disease with Tongxinluo and Meiluxin (通心络与美乐心治疗冠心病心绞痛临床观察). Occupation and Health, 2003(12): 153-154.

[11] Yuan XM, Yang F, Zhang ZZ, et al. Clinical study on the treatment of coronary heart disease in the elderly by Tongxinluo capsule (通心络胶囊治疗老年人冠心病的临床研究). China Medical Journal Today, 2003, 3(19): 11-12.

[12] Wang JC, Sun XY, Li HB, et al. Comparative Study of Tongxinluo Capsules and Sordi in Treatment of Patients with Silent Myocardial Ischemia (通心络胶囊和硝酸异山梨醇治疗无症状心肌缺血的对照研究). Chinese Journal of Basic Medicine in Traditional Chinese Medicine, 2001, 7(1): 49-51.

7 Outcomes of interest not reported or data not available (n = 38)

[1] Gu WJ, Zhang R, Zhao TT. Therapeutic effect of Tongxinluo capsule combined atorvastatin on coronary heart disease and its influence on serum levels of cTnI, cTnT and hsCRP (通心络胶囊联合阿托伐他汀治疗冠心病的疗效及其对血清 cTnI、cTnT、hs-CRP 水平的影响). Chinese Journal of cardiovascular Rehabilitation Medicine, 2020, 29(04): 471-474.

[2] Liu H, Lv Z. Analysis Of The Improvement Of Tongxinluo Capsule On Myocardial Microcirculation And Left Ventricular Remodeling After PCI In Patients With Acute Myocardial Infarction (通心络胶囊辅助治疗对急性心肌梗死患者 PCI 术后心肌微循环和左室重构的改善分析). World Journal of Integrated Traditional and Western Medicine, 2020, 15(10): 1926-1930.

[3] Chen C, Zhong W, Kuang CQ. Clinical efficacy of Tongxinluo capsule in the adjuvant treatment of stable angina pectoris and its effect on endothelial cell function (通心络胶囊辅助治疗稳定型心绞痛临床疗效及对内皮细胞功能的影响). Journal of clinical rational drug use, 2019, 12(31): 53-54.

[4] Liang B, Che JJ, Dou S. Clinical study of Tongxinluo capsule combined with Rosuvastatin in the treatment of elderly patients with coronary heart disease (通心络胶囊联合瑞舒伐他汀治疗老年冠心病患者的临床研究). The Journal of Medical Theory and Practice, 2019, 32(19): 3076-3077.

[5] Li JY, Guo ZG. Observation of the efficacy of Tongxinluo capsule on patients after coronary intervention and its effect on vascular endothelial function and inflammatory factors (通心络胶囊对冠心病介入术后患者疗效观察及对血管内皮功能和炎症因子影响). Chinese Traditional Patent Medicine, 2019, 41(05): 1202-1204.

[6] Xu YC, He T, Hu W, et al. Therapeutic Effect of Pravastatin Combined With Tongxinluo On Coronary Heart Disease Complicated With Hypertension (普伐他汀联合通心络治疗冠心病合并高血压疗效观察). China Continuing Medical Education, 2019, 11(16): 141-143.

[7] Wei YT, Song DQ. Effect of Tongxinluo capsule on stable angina pectoris induced by coronary heart disease (通心络胶囊治疗冠心病稳定性心绞痛效果分析). Journal of Qiqihar Medical College, 2018, 39(22): 2651-2653.

[8] Zou Y, Sun YR. Effect of fasudil combined with tongxinluo on serum inflammatory factors in hypertensive acute myocardial infarction in post-PCI patients (法舒地尔联合通心络对高血压急性心肌梗死 PCI 术后患者血清炎性因子的影响). Modern Journal of Integrated Traditional Chinese and Western Medicine, 2018, 27(19): 2105-2108.

[9] Xiao P, Ma C, Wang WW, et al. Clinical efficacy of Tongxinluo capsule in stable angina pectoris and its effect on endothelial cell function (通心络胶囊对稳定型心绞痛的临床疗效及其对内皮细胞功能的作用研究). Modern Diagnosis and Treatment, 2017, 28(09): 1596-1597.

[10] Liu XW, Zhang XZ, Zhang FL. Effect of Oral Tongxinluo Capsule on Endothelial Microparticles and Matrix Metalloproteinase-9 in Serum of Patients with Acute Myocardial Infarction after Percutaneous Coronary Intervention (急性心肌梗死患者介入治疗后口服通心络胶囊对血清内皮细胞微粒及基质金属蛋白酶 9 水平的影响). Chinese Journal of Pharmacovigilance, 2017, 14(03): 142-144.

[11] Wang YL, Cheng JL, Li JR, et al. Protective effect of myocardial and microvascular of tongxinluo capsule on patients of acute myocardial infarction after percutaneous coronary intervention (通心络胶囊对急性心肌梗死患者经皮冠状动脉介入治疗术后心肌和微血管的保护作用研究). Practical Journal of Cardiac Cerebral Pneumal and Vascular Disease, 2016, 24(02): 150-152.

[12] Li HK, Lu YG, Yan H, et al. Influence of tongxinluo on blood endothelial microparticles and MMP-9 in patients with acute myocardial infarction after percutaneous coronary intervention (通心络对急性心肌梗死患者 PCI 术后血清 EMPs 及 MMP-9 的影响). Chongqing Medicine, 2016, 45(03): 354-355.

[13] Wu L, Li L. Observation on the efficacy of Tongxinluo capsule in the treatment of stable angina pectoris of coronary heart disease and its effect on blood lipids (通心络胶囊治疗冠心病稳定型心绞痛的疗效观察及对血脂的影响). Journal of Shanxi Medical College for Continuing Education, 2015, 25(06): 34-35.
[14] Chen CL, Ma MK, Chen N. Clinical efficacy observation of Tongxinluo capsule in the treatment of stable angina pectoris (通心络胶囊治疗稳定型心绞痛临床疗效观察). Medical Aesthetics and Beauty, 2014(6): 226.
[15] Zhang XY, Liu XC, Wang HH, et al. Effect of Tongxinluo capsule on Lp-PLA2 and vascular endothelial function after PCI in patients with acute coronary syndrome (通心络胶囊对急性冠脉综合征患者 PCI 术后 Lp-PLA2 及血管内皮功能的影响). Chinese Journal of Integrative Medicine on Cardio-/Cerebrovascular Disease, 2014, 12(01): 30-31.
[16] Liu JL, He YJ, Wu S, et al. Clinical observation of Tongxinluo capsule on acute myocardial infarction after coronary stents with no-reflow (通心络胶囊对急性心肌梗死患者急诊支架置入术中无复流的临床观察). Chinese Journal of Difficult and Complicated Cases, 2014, 13(01): 80-82.
[17] Lu Y, Zhao XJ, Liao XC. Effect of Tongxinluo capsule on blood rheology after PCI in patients with coronary artery disease (通心络胶囊对冠心病患者 PCI 术后血液流变学的影响). Nei Mongol Journal of Traditional Chinese Medicine, 2012, 31(08): 1-2.
[18] Yang W, Xu XH, Wang S, et al. Effect of Tongxinluo capsule on serum type III procollagen amino-terminal peptide and left ventricular remodeling in patients after emergency PCI for acute myocardial infarction (通心络胶囊对急性心肌梗死急诊 PCI 术后患者血清Ⅲ型前胶原氨基端肽及左室重构的影响). Chinese Journal of Difficult and Complicated Cases, 2012, 11(06): 452-453.
[19] Yang W, Wang S, Yu XH, et al. Effect of Tongxinluo capsule on left ventricular remodeling after emergency PCI for acute myocardial infarction (通心络胶囊对急性心肌梗死急诊 PCI 术后左室重构的影响). Chinese Community Physician, 2012, 28(30): 8.
[20] Chen ZQ, Hong L, Wang H, et al. Effects of Tongxinluo Capsule on Platelet Activity and Vascular Endothelium Function at Different Stages in Patients with Acute Coronary Syndrome Undergoing Percutaneous Coronary Intervention (通心络胶囊对急性冠状动脉综合征患者介入治疗后不同时期血小板活化及血管内皮功能的影响). Chinese Pharmaceutical Journal, 2012, 47(04): 311-315.
[21] Qian XX, Chen YM, Liu Y, et al. Clinical efficacy of Tongxinluo in the treatment of stable angina pectoris and its effect on endothelial function (通心络治疗稳定型心绞痛的临床疗效及对内皮功能的影响). Chinese Community Physician, 2011, 27(35): 8.
[22] Chen ZQ, Hong L, Yin QL, et al. Effect of Tongxinluo on platelet activation and vascular endothelial function after intervention in patients with acute coronary syndrome (通心络对急性冠脉综合征患者介入术后血小板活化及血管内皮功能的影响). Chinese Community Physician, 2011, 27(24): 11.
[23] Li YX, Li AR, Zhang ZH, et al. Tongxinluo auxiliary treatment for angina pectoris after coronary heart disease stenting: clinical observation of 35 cases (通心络辅治冠心病支架术后心绞痛 35 例临床观察). Chinese Journal of Difficult and Complicated Cases, 2010, 9(03): 205-206.
[24] Li KH, Li JH. Curative Effect Observing of Tongxinluo Capsule Plus Routine Western Medicine Therapy for Angina Pectoris (通心络配合西药综合治疗冠心病心绞痛疗效观察). Journal of Liaoning University of Traditional Chinese Medicine, 2009, 11(07): 101-102.
[25] Wang XG, Xu ZF, Xu ZX. Effect of Tongxinluo Capusle on the Pre-thrombotic State in Patients with Coronary Heart Disease and Stable Angina Pectoris (通心络胶囊对冠心病稳定型心绞痛患者血栓前状态的影响). Practical Clinical Medicine, 2009, 10(07): 25-27.
[26] Ma QL, Zhang SD, Ning YG, et al. Effect of Tongxinluo on endothelial function and hypersensitive C-reactive protein in acute coronary syndrome patients undergoing percutaneous coronary intervention (通心络对急性冠脉综合征介入治疗术患者内皮功能和高敏 C 反应蛋白的影响). Journal of Central South University (Medical Sciences), 2009, 34(6): 550-554.

[27] Zhang Y, Chang YP, Zhang Y, et al. Observation of the chronic stable angina treated by Tongxinluo capsule (通心络胶囊治疗慢性稳定性心绞痛的临床观察). Hebei Journal of Traditional Chinese Medicine, 2008, 30(11): 1203-1204.

[28] Han HY, Li MJ, Wan WG, et al. Effects of Tongxinluo capsules on inflammatory reaction and vascular endothelium function after PCI (通心络胶囊对冠心病介入术后炎性反应和血管内皮功能的影响). Chinese Journal of Difficult and Complicated Cases, 2008(11): 663-665.

[29] Jiang WQ, Lai JX. Tongxinluo capsule treatment of geriatric coronary heart disease 50 cases efficacy observation (通心络胶囊治疗老年性冠心病 50 例疗效观察). Nei Mongol Journal of Traditional Chinese Medicine, 2008(13): 67-68.

[30] Luo Q, Wang GG. Therapeutic effect of Tongxinluo capsule on patients with acute myocardial infarction after hemodilution reconstruction (通心络胶囊对急性心肌梗死患者血运重建后疗效观察). Nei Mongol Journal of Traditional Chinese Medicine, 2008(05): 7.

[31] Tao Y, Dai XH, Dong M, et al. Observation on the efficacy of Tongxinluo capsule in the treatment of angina pectoris of coronary heart disease (通心络胶囊治疗冠心病心绞痛疗效观察). Clinical Journal of Traditional Chinese Medicine, 2007(04): 370-371.

[32] Li HR, Pan Y. Clinical observation of Tongxinluo capsule in the treatment of stable angina pectoris in coronary heart disease (通心络胶囊治疗冠心病稳定型心绞痛临床观察). Modern Journal of Integrated Traditional Chinese and Western Medicine, 2007(03): 297-298.

[33] Chen ZQ, Lai YY, Hong L, et al. The effects of Tongxinluo capsule on platelet activity and vascular endothelium function in patients with ACS after PCI (通心络对急性冠状动脉综合征患者介入术后血小板活化及血管内皮功能的影响). Chinese Journal of Geriatric Heart Brain and Vessel Diseases, 2007,9(7):460-463.

[34] Guo ZH, Jin ZH, Tang HY. Effect of Tongxinluo capsule on vascular endothelial function in patients with stable angina pectoris (通心络胶囊对稳定型心绞痛患者血管内皮功能的影响). Journal of Shandong University of Traditional Chinese Medicine, 2006(04): 297-298.

[35] You SJ, Chen KJ, Yang YJ, et al. Clinical Study on Spontaneous Improvement after Blood Flow Reconstruction Interfered by Tongxinluo Capsule in Patients with Early Stage Acute Myocardial Infarction (通心络胶囊干预急性心肌梗死早期血运重建后自发性改善的临床研究). Chinese Journal of Integrated Traditional and Western Medicine, 2005, 25(7): 604-607.

[36] You SJ, Yang YJ, Chen KJ, et al. Efficacy and safety of Tongxinluo capsule in patients with acute myocardial infarction after reperfusion (通心络胶囊在急性心肌梗死血运重建后的有效性和安全性研究). Chinese Journal of Difficult and Complicated Cases, 2004, 3(4): 193-196.

[37] You SJ, Yang YJ, Chen KJ, et al. Efficacy and safety study of Tongxinluo capsule after hemodilution reconstruction in acute myocardial infarction (通心络胶囊在急性心肌梗死血运重建后的有效性和安全性研究). Chinese Journal of Cardiology, 2004, 32(z1): 132-138.

[38] Xing PY, Jia LW, Du YY, Zhang JL. Therapeutic effect of Tongxinluo capsule on myocardial ischemia in elderly coronary heart disease (通心络胶囊对老年冠心病心肌缺血的治疗作用). Zhejiang Journal of Integrated Traditional Chinese and Western Medicine, 2003(04): 37-38.

8 The duration of treatment was unclear, or the duration of treatment was inconsistent between the treatment and control groups (n = 2)

[1] Chen WG, Yang QB, Chen J, et al. Far and near clinical effect of tongxinluo after interventional treatment of coronary heart disease(CHD) (通心络在冠心病介入治疗后远近期的临床效果体会). China Medicine and Pharmacy, 2016, 6(13): 96-98.

[2] Zhang XP, Zhang Y. Effect of Tongxinluo on the long-term outcome of patients with acute myocardial infarction after stent placement (通心络对急性心肌梗死患者置入支架后远期疗效的影响). Chinese Remedies & Clinics, 2009, 9(03): 243-244.
