# Supplementary material for: Chinese patent medicine tongxinluo capsule as a supplement to treat chronic coronary syndromes: a GRADE-assessed systematic review and meta-analysis of randomized controlled trials
Source: Front Cardiovasc Med. 2025 Jan 7;11:1499585. doi: 10.3389/fcvm.2024.1499585 (PMC11753206; doi:10.3389/fcvm.2024.1499585)
Supplement: Supplementary file 3 [file Datasheet3.pdf]

Supplementary Material 3 Summary of drugs used in the control group

Drugs	References	
Anticoagulant drugs	(1) Aspirin (Cui S2023; Zhao XJ2023; Cheng L2023; Zhu YC2020; Zhang CC2019; Liu J2018; Wei ZH2009; Li L2009; Ye HL2009; Zhao JL2008; Chen Q2007)<br>(2) low-molecular heparin (Low-molecular heparin calcium [Cheng L2023]; Low-molecular heparin sodium (LMS) [Cui S2023; Yang CJ2020])	
Nitrate drugs	(1) Nitroglycerine (Cui S2023; Zhao XJ2023; Cheng L2023; Zhang CC2019; Xing XH2015#; Li L2009*; Ye HL2009; Chen Q2007*; Wang JZ2003) [#: Intravenous nitroglycerine 20-80 μ g/min; *: Sublingually during angina attacks]<br>(2) Isosorbide mononitrate (Zhao XJ2023; Yang CJ2020; Xing XH2015)<br>(3) Isosorbide dinitrate (Ye HL2009; Liu HM2006; Wang JZ2003; Jin JJ2003)	No specific medication reported (Wang XP2021; Yang CJ2020; Liu J2018; Wei ZH2009; Li L2009; Zhao JL2008; Chen Q2007)
β- receptor blockers	Metoprolol (Zhao XJ2023; Wang JC2022; Wang F2021; Wang XP2021; Yang CJ2020; Zhu YC2020; Liu HM2006)	No specific medication reported (Cui S2023; Cheng L2023; Zhang CC2019; Liu J2018; Xing XH2015; Wei ZH2009; Ye HL2009; Zhao JL2008; Chen Q2007)
Vasodilator drugs	(1) Angiotensin converting enzyme inhibitors (ACEI) (Cui S2023; Cheng L2023; Zhang CC2019; Wei ZH2009; Chen Q2007)<br>(2) Angiotensin II receptor antagonists (ARBs) (Lv WZ2012)<br>(3) Trimetazidine dihydrochloride (Zhang CC2019)	No specific medication reported (Wang F2021)
Antiplatelet aggregating agents	(1) Clopidogrel (Cui S2023; Cheng L2023; Yang CJ2020; Wei ZH2009)<br>(2) Tirofiban (Cui S2023)	No specific medication reported (Wang JC2022; Wang F2021; Wang XP2021)
Lipid-lowering drugs	Statins (Cui S2023; Zhao XJ2023; Yang CJ2020; Zhu YC2020; Wei ZH2009; Zhao JL2008; Chen Q2007)	No specific medication reported (Cheng L2023; Wang F2021; Wang XP2021; Li L2009; Ye HL2009)
Calcium channel blockers	(1) Amlodipine besylate tablets (Zhu YC2020)<br>(2) Nifedipine tablets (Liu HM2006)	No specific medication reported (Wang XP2021; Wei ZH2009; Ye HL2009; Chen Q2007)
Other drugs	Hypoglycaemic drugs (Cheng L2023; Zhao XJ2023)	
