# Supplementary material for: Chinese patent medicine tongxinluo capsule as a supplement to treat chronic coronary syndromes: a GRADE-assessed systematic review and meta-analysis of randomized controlled trials
Source: Front Cardiovasc Med. 2025 Jan 7;11:1499585. doi: 10.3389/fcvm.2024.1499585 (PMC11753206; doi:10.3389/fcvm.2024.1499585)
Supplement: Supplementary file 4 [file Datasheet4.pdf]

Supplementary Material 4 Figures for baseline analyses, sensitivity analyses and subgroup analyses

1. Forest plot of MI occurrence - outcome (fixed effects; sensitivity analysis)

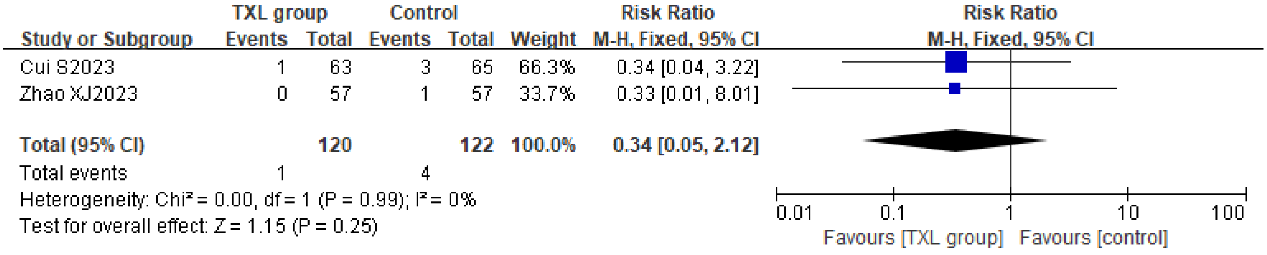

Supplementary Material 4--fig. 1

2. Forest plot of angina frequency (baseline analysis)

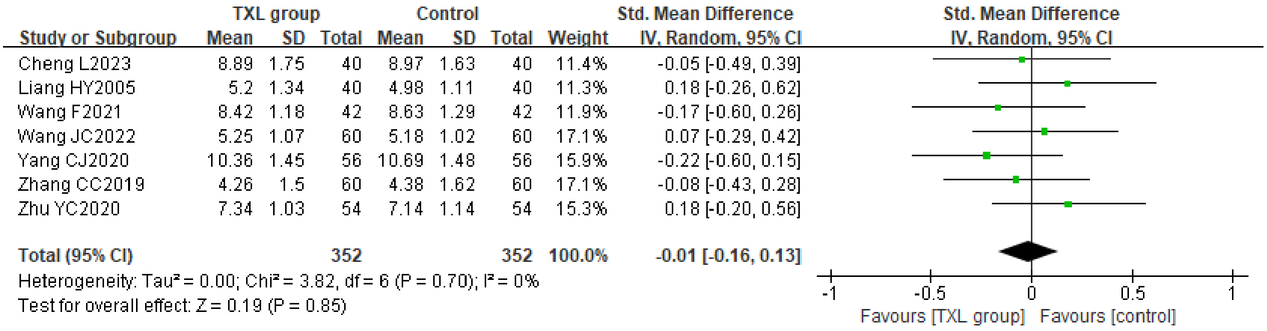

Supplementary Material 4--fig. 2

3. Forest plot of angina frequency - outcome (fixed effects; sensitivity analysis)

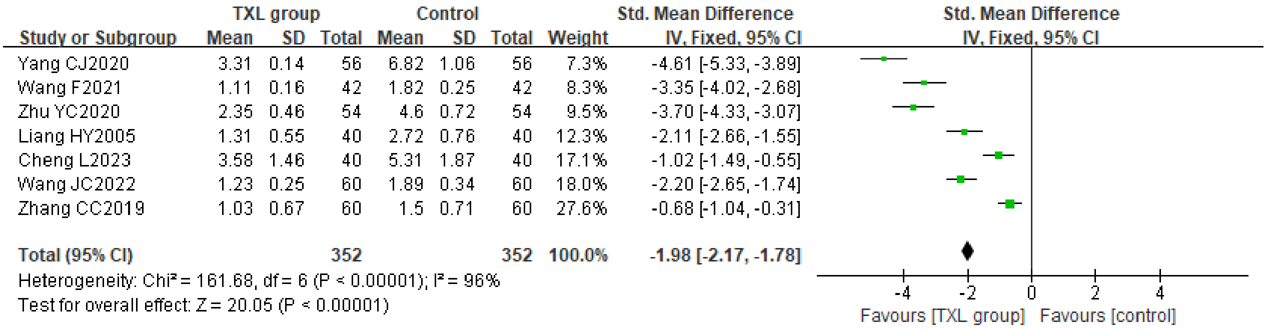

Supplementary Material 4--fig. 3

4. Forest plot of angina frequency (subgroup analysis)

Study or Subgroup	TXL group Mean	SD	Total	Control Mean	SD	Total	Weight	Mean Difference IV, Random, 95% CI
**1.7.1 4 weeks treatment (2-4 capsules; bid/tid)**								
Yang CJ2020	3.31	0.14	56	6.82	1.06	56	20.4%	-3.51 [-3.79, -3.23]
Zhang CC2019	1.03	0.67	60	1.5	0.71	60	20.5%	-0.47 [-0.72, -0.22]
**Subtotal (95% CI)**			**116**			**116**	**40.9%**	**-1.99 [-4.97, 0.99]**
Heterogeneity: Tau² = 4.60; Chi² = 254.61, df = 1 (P < 0.00001); I² = 100%								
Test for overall effect: Z = 1.31 (P = 0.19)								
**1.7.2 12 weeks (3 months) treatment (4 capsules; tid)**								
Cheng L2023	3.58	1.46	40	5.31	1.87	40	17.4%	-1.73 [-2.47, -0.99]
Wang F2021	1.11	0.16	42	1.82	0.25	42	20.9%	-0.71 [-0.80, -0.62]
Wang JC2022	1.23	0.25	60	1.89	0.34	60	20.9%	-0.66 [-0.77, -0.55]
**Subtotal (95% CI)**			**142**			**142**	**59.1%**	**-0.74 [-0.92, -0.56]**
Heterogeneity: Tau² = 0.02; Chi² = 8.12, df = 2 (P = 0.02); I² = 75%								
Test for overall effect: Z = 8.12 (P < 0.00001)								
**Total (95% CI)**			**258**			**258**	**100.0%**	**-1.40 [-2.13, -0.66]**
Heterogeneity: Tau² = 0.68; Chi² = 382.35, df = 4 (P < 0.00001); I² = 99%								
Test for overall effect: Z = 3.71 (P = 0.0002)								
Test for subgroup differences: Chi² = 0.67, df = 1 (P = 0.41), I² = 0%								

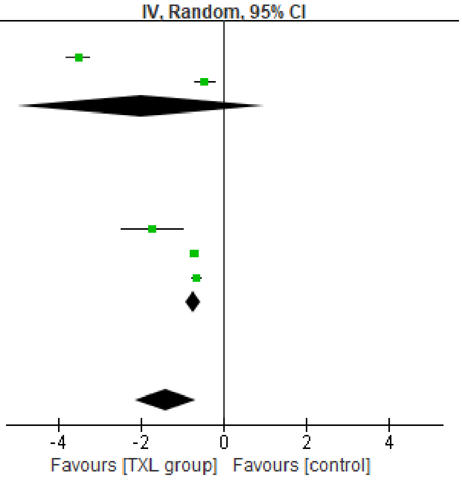

Supplementary Material 4--fig. 4

5. Forest plot of angina duration (baseline analysis)

Study or Subgroup	TXL group Mean	SD	Total	Control Mean	SD	Total	Weight	Mean Difference IV, Random, 95% CI
Cheng L2023	7.41	0.79	40	7.15	0.97	40	23.4%	0.26 [-0.13, 0.65]
Liang HY2005	5.78	2.67	40	5.35	2.11	40	3.2%	0.43 [-0.62, 1.48]
Wang F2021	6.74	0.88	42	6.85	1.03	42	20.9%	-0.11 [-0.52, 0.30]
Wang JC2022	5.79	1.13	60	5.82	1.16	60	20.9%	-0.03 [-0.44, 0.38]
Yang CJ2020	8.67	1.15	56	8.61	1.44	56	15.1%	0.06 [-0.42, 0.54]
Zhang CC2019	13.5	4.02	60	12.87	43.89	60	0.0%	0.63 [-10.52, 11.78]
Zhu YC2020	8.86	1.22	54	8.61	1.22	54	16.6%	0.25 [-0.21, 0.71]
**Total (95% CI)**			**352**			**352**	**100.0%**	**0.10 [-0.09, 0.28]**
Heterogeneity: Tau² = 0.00; Chi² = 2.87, df = 6 (P = 0.83); I² = 0%								
Test for overall effect: Z = 1.00 (P = 0.32)								

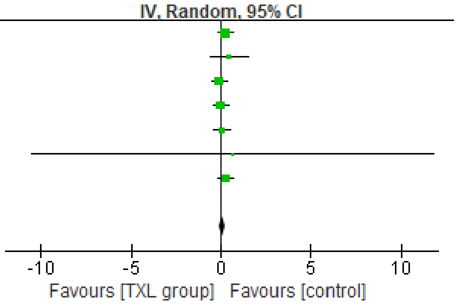

Supplementary Material 4--fig. 5

6. Forest plot of angina duration - outcome (fixed effects; sensitivity analysis)

Study or Subgroup	TXL group Mean	SD	Total	Control Mean	SD	Total	Weight	Mean Difference IV, Fixed, 95% CI
Cheng L2023	3.28	0.42	40	5.61	0.57	40	15.5%	-2.33 [-2.55, -2.11]
Liang HY2005	2.73	0.78	40	2.98	1.56	40	2.6%	-0.25 [-0.79, 0.29]
Wang F2021	2.32	0.26	42	3.56	0.5	42	25.7%	-1.24 [-1.41, -1.07]
Wang JC2022	2.14	0.38	60	3.05	0.42	60	36.4%	-0.91 [-1.05, -0.77]
Yang CJ2020	3.64	0.21	56	5.38	1.12	56	8.4%	-1.74 [-2.04, -1.44]
Zhang CC2019	2.31	0.92	60	4.1	1.27	60	4.7%	-1.79 [-2.19, -1.39]
Zhu YC2020	4.27	0.71	54	6.38	1.03	54	6.7%	-2.11 [-2.44, -1.78]
**Total (95% CI)**			**352**			**352**	**100.0%**	**-1.39 [-1.48, -1.30]**
Heterogeneity: Chi² = 160.73, df = 6 (P < 0.00001); I² = 96%								
Test for overall effect: Z = 31.53 (P < 0.00001)								

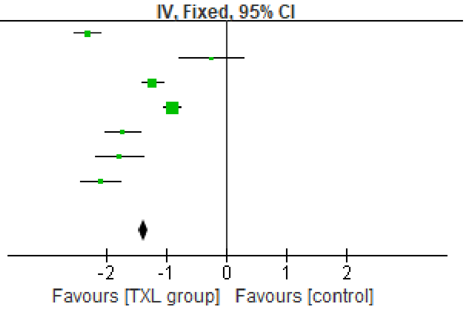

Supplementary Material 4--fig. 6

7. Forest plot of angina duration (sensitivity analysis for the source of heterogeneity)

Study or Subgroup	TXL group Mean	SD	Total	Control Mean	SD	Total	Weight	Mean Difference IV, Random, 95% CI
Cheng L2023	3.28	0.42	40	5.61	0.57	40	28.8%	-2.33 [-2.55, -2.11]
Liang HY2005	2.73	0.78	40	2.98	1.56	40	0.0%	-0.25 [-0.79, 0.29]
Wang F2021	2.32	0.26	42	3.56	0.5	42	0.0%	-1.24 [-1.41, -1.07]
Wang JC2022	2.14	0.38	60	3.05	0.42	60	0.0%	-0.91 [-1.05, -0.77]
Yang CJ2020	3.64	0.21	56	5.38	1.12	56	25.6%	-1.74 [-2.04, -1.44]
Zhang CC2019	2.31	0.92	60	4.1	1.27	60	21.5%	-1.79 [-2.19, -1.39]
Zhu YC2020	4.27	0.71	54	6.38	1.03	54	24.1%	-2.11 [-2.44, -1.78]
**Total (95% CI)**			**210**			**210**	**100.0%**	**-2.01 [-2.31, -1.71]**
Heterogeneity: Tau² = 0.07; Chi² = 12.05, df = 3 (P = 0.007); I² = 75%								
Test for overall effect: Z = 12.92 (P < 0.00001)								

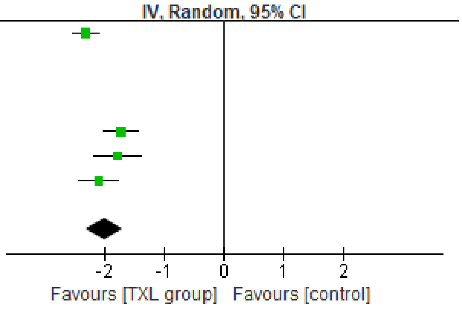

Supplementary Material 4--fig. 7

8. Forest plot of angina duration (subgroup analysis)

Study or Subgroup	TXL group Mean	SD	Total	Control Mean	SD	Total	Weight	Mean Difference IV, Random, 95% CI
**1.11.1 4 weeks treatment (2-4 capsules; bid/tid)**								
Liang HY2005	2.73	0.78	40	2.98	1.56	40	12.8%	-0.25 [-0.79, 0.29]
Yang CJ2020	3.64	0.21	56	5.38	1.12	56	14.4%	-1.74 [-2.04, -1.44]
Zhang CC2019	2.31	0.92	60	4.1	1.27	60	13.8%	-1.79 [-2.19, -1.39]
**Subtotal (95% CI)**			**156**			**156**	**41.0%**	**-1.29 [-2.11, -0.47]**
Heterogeneity: Tau² = 0.48; Chi² = 25.07, df = 2 (P < 0.00001); I² = 92%								
Test for overall effect: Z = 3.08 (P = 0.002)								
**1.11.2 8 weeks treatment (3 capsules; tid)**								
Zhu YC2020	4.27	0.71	54	6.38	1.03	54	14.2%	-2.11 [-2.44, -1.78]
**Subtotal (95% CI)**			**54**			**54**	**14.2%**	**-2.11 [-2.44, -1.78]**
Heterogeneity: Not applicable								
Test for overall effect: Z = 12.39 (P < 0.00001)								
**1.11.3 12 weeks (3 months) treatment (4 capsules; tid)**								
Cheng L2023	3.28	0.42	40	5.61	0.57	40	14.8%	-2.33 [-2.55, -2.11]
Wang F2021	2.32	0.26	42	3.56	0.5	42	15.0%	-1.24 [-1.41, -1.07]
Wang JC2022	2.14	0.38	60	3.05	0.42	60	15.0%	-0.91 [-1.05, -0.77]
**Subtotal (95% CI)**			**142**			**142**	**44.8%**	**-1.49 [-2.25, -0.73]**
Heterogeneity: Tau² = 0.44; Chi² = 113.57, df = 2 (P < 0.00001); I² = 98%								
Test for overall effect: Z = 3.84 (P = 0.0001)								
**Total (95% CI)**			**352**			**352**	**100.0%**	**-1.50 [-1.98, -1.02]**
Heterogeneity: Tau² = 0.39; Chi² = 160.73, df = 6 (P < 0.00001); I² = 96%								
Test for overall effect: Z = 6.10 (P < 0.00001)								
Test for subgroup differences: Chi² = 4.76, df = 2 (P = 0.09), I² = 58.0%								

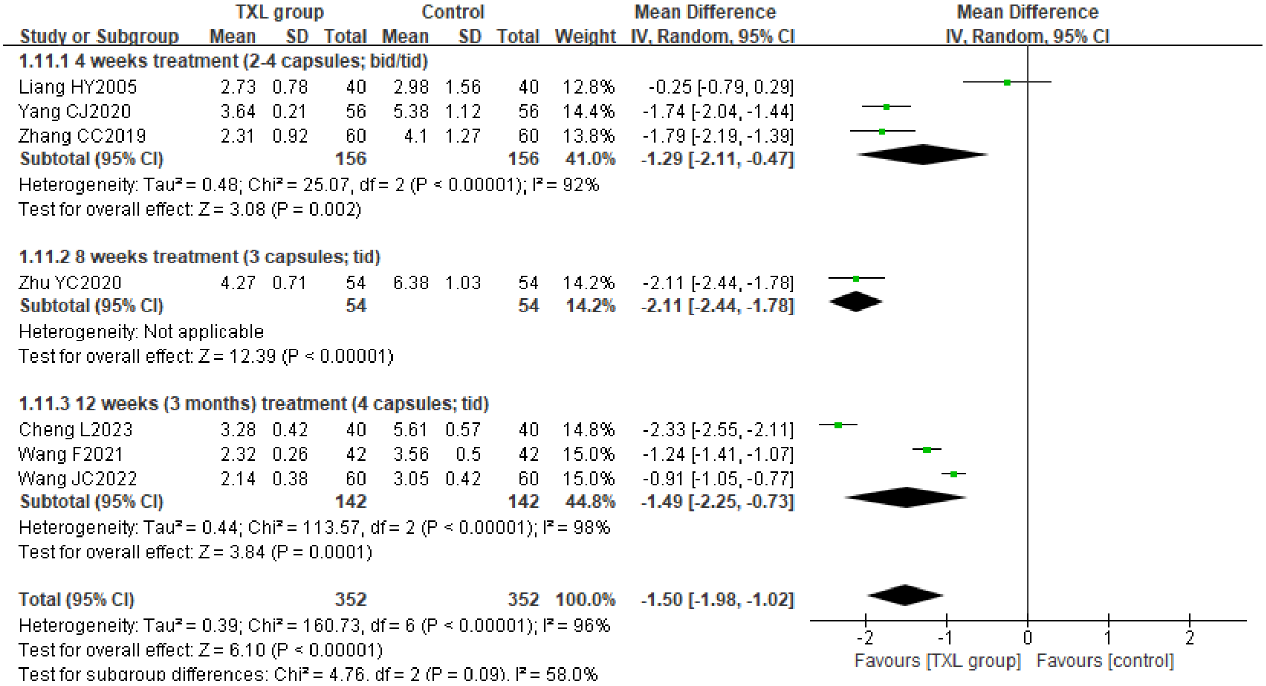

Supplementary Material 4--fig. 8

9. Forest plot of nitroglycerin dosage (baseline analysis)

Study or Subgroup	TXL group Mean	SD	Total	Control Mean	SD	Total	Weight	Std. Mean Difference IV, Random, 95% CI
Cheng L2023	1.82	0.29	40	1.76	0.52	40	21.2%	0.14 [-0.30, 0.58]
Liang HY2005	6.52	1.78	40	5.98	1.11	40	20.9%	0.36 [-0.08, 0.80]
Ye HL2009	8.6	2.4	50	8.7	2.3	50	26.5%	-0.04 [-0.43, 0.35]
Zhang CC2019	26.93	4.22	60	25.5	4.58	60	31.4%	0.32 [-0.04, 0.68]
**Total (95% CI)**			**190**			**190**	**100.0%**	**0.20 [-0.01, 0.40]**
Heterogeneity: Tau² = 0.00; Chi² = 2.49, df = 3 (P = 0.48); I² = 0%								
Test for overall effect: Z = 1.90 (P = 0.06)								

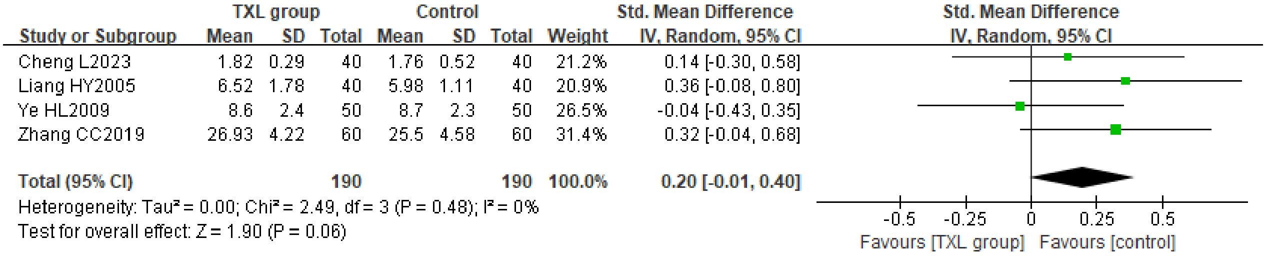

Supplementary Material 4--fig. 9

10. Forest plot of nitroglycerin dosage - outcome (fixed effects; sensitivity analysis)

Study or Subgroup	TXL group Mean	SD	Total	Control Mean	SD	Total	Weight	Std. Mean Difference IV, Fixed, 95% CI
Cheng L2023	0.51	0.13	40	1.14	0.95	40	25.6%	-0.92 [-1.38, -0.46]
Liang HY2005	1.67	0.55	40	2.72	0.49	40	18.7%	-2.00 [-2.54, -1.46]
Ye HL2009	2.8	2.5	50	8.1	1.9	50	20.6%	-2.37 [-2.88, -1.85]
Zhang CC2019	4.16	1.32	60	6.02	1.53	60	35.1%	-1.29 [-1.69, -0.90]
**Total (95% CI)**			**190**			**190**	**100.0%**	**-1.55 [-1.78, -1.32]**
Heterogeneity: Chi² = 21.06, df = 3 (P = 0.0001); I² = 86%								
Test for overall effect: Z = 12.99 (P < 0.00001)								

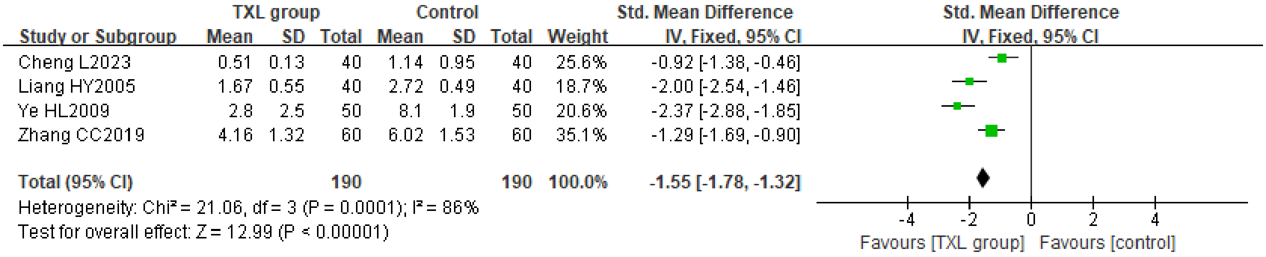

Supplementary Material 4--fig. 10

11. Forest plot of nitroglycerin dosage (sensitivity analysis for the source of heterogeneity)

Study or Subgroup	TXL group Mean	SD	Total	Control Mean	SD	Total	Weight	Std. Mean Difference IV, Random, 95% CI
Cheng L2023	0.51	0.13	40	1.14	0.95	40	0.0%	-0.92 [-1.38, -0.46]
Liang HY2005	1.67	0.55	40	2.72	0.49	40	47.6%	-2.00 [-2.54, -1.46]
Ye HL2009	2.8	2.5	50	8.1	1.9	50	52.4%	-2.37 [-2.88, -1.85]
Zhang CC2019	4.16	1.32	60	6.02	1.53	60	0.0%	-1.29 [-1.69, -0.90]
**Total (95% CI)**			**90**			**90**	**100.0%**	**-2.19 [-2.56, -1.82]**
Heterogeneity: Tau² = 0.00; Chi² = 0.95, df = 1 (P = 0.33); I² = 0%								
Test for overall effect: Z = 11.51 (P < 0.00001)								

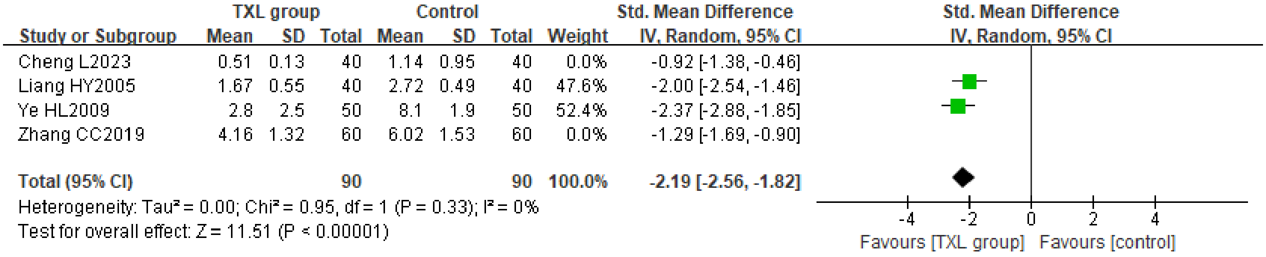

Supplementary Material 4--fig. 11

12. Forest plot of nitroglycerin dosage (sensitivity analysis for the source of heterogeneity)

Study or Subgroup	TXL group Mean	TXL group SD	TXL group Total	Control Mean	Control SD	Control Total	Weight	Std. Mean Difference IV, Random, 95% CI
Cheng L2023	0.51	0.13	40	1.14	0.95	40	44.6%	-0.92 [-1.38, -0.46]
Liang HY2005	1.67	0.55	40	2.72	0.49	40	0.0%	-2.00 [-2.54, -1.46]
Ye HL2009	2.8	2.5	50	8.1	1.9	50	0.0%	-2.37 [-2.88, -1.85]
Zhang CC2019	4.16	1.32	60	6.02	1.53	60	55.4%	-1.29 [-1.69, -0.90]
**Total (95% CI)**			**100**			**100**	**100.0%**	**-1.13 [-1.49, -0.76]**

Heterogeneity: Tau² = 0.02; Chi² = 1.45, df = 1 (P = 0.23); I² = 31%
Test for overall effect: Z = 6.07 (P < 0.00001)

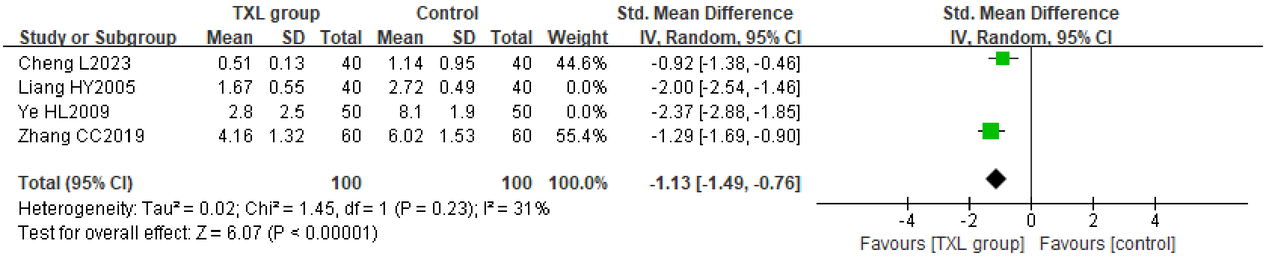

Supplementary Material 4--fig. 12

13. Forest plot of adverse events - outcome (fixed effects; sensitivity analysis)

Study or Subgroup	TXL group Events	TXL group Total	Control Events	Control Total	Weight	Risk Ratio M-H, Fixed, 95% CI
Chen Q2007	8	40	6	40	9.0%	1.33 [0.51, 3.49]
Jin JJ2003	1	30	0	28	0.8%	2.81 [0.12, 66.17]
Liu HM2006	1	44	0	44	0.7%	3.00 [0.13, 71.70]
Liu J2018	11	90	19	90	28.4%	0.58 [0.29, 1.15]
Wang F2021	10	42	8	42	12.0%	1.25 [0.55, 2.85]
Wang JC2022	8	60	6	60	9.0%	1.33 [0.49, 3.61]
Wang JZ2003	0	80	10	80	15.7%	0.05 [0.00, 0.80]
Wang XP2021	2	48	2	48	3.0%	1.00 [0.15, 6.81]
Wei ZH2009	2	50	0	48	0.8%	4.80 [0.24, 97.55]
Yang CJ2020	3	56	2	56	3.0%	1.50 [0.26, 8.64]
Ye HL2009	3	50	0	50	0.7%	7.00 [0.37, 132.10]
Zhao JL2008	8	60	10	56	15.5%	0.75 [0.32, 1.76]
Zhao XJ2023	3	57	1	57	1.5%	3.00 [0.32, 27.99]
**Total (95% CI)**		**707**		**699**	**100.0%**	**0.93 [0.67, 1.28]**
Total events	60		64			

Heterogeneity: Chi² = 13.20, df = 12 (P = 0.35); I² = 9%
Test for overall effect: Z = 0.45 (P = 0.66)

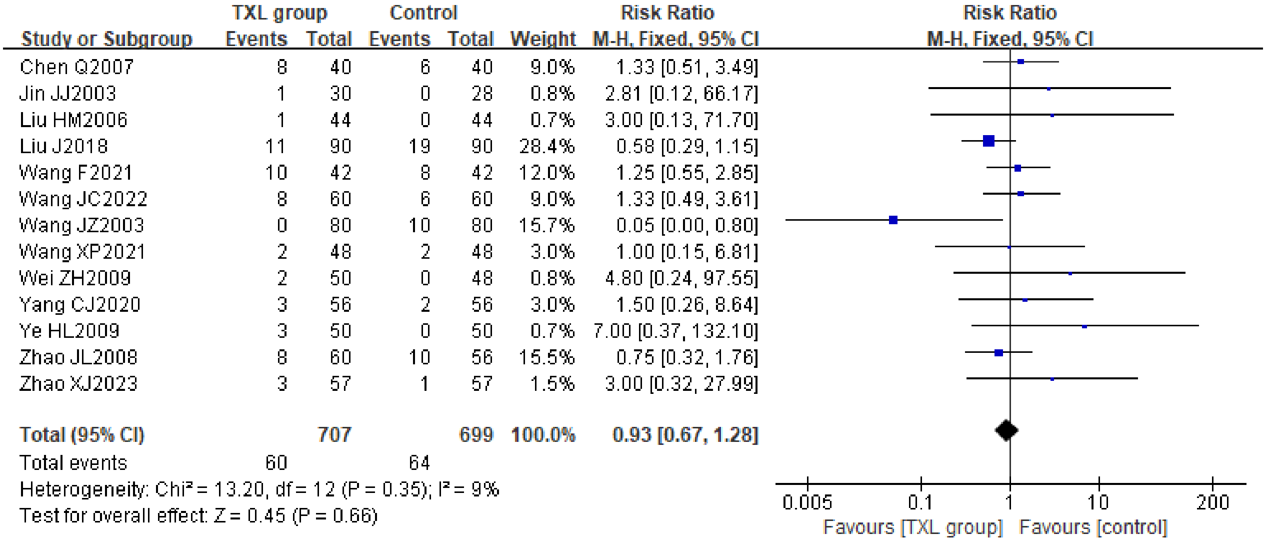

Supplementary Material 4--fig. 13
